# Stepwise Spin Transitions of Spin-Crossover Complexes Based in 3-(2-Pyridyl)-1,2,4-triazole Ligands Associated with Symmetry Change in Hydrogen Bonding Interactions

**DOI:** 10.1021/acs.inorgchem.4c04952

**Published:** 2025-03-25

**Authors:** Yuliia P. Petrenko, José Troya, Víctor García-López, Dmytro M. Khomenko, Roman O. Doroshchuk, Rostyslav D. Lampeka, Miguel Clemente-León, Eugenio Coronado

**Affiliations:** † Instituto de Ciencia Molecular (ICMol), Universidad de Valencia, Catedrático José Beltrán 2, 46980 Paterna, Spain; ‡ Department of Chemistry, Taras Shevchenko National University of Kyiv, 12, Hetman Pavlo Skoropadsky St., 01033 Kyiv, Ukraine

## Abstract

Iron­(II) compounds of a family of 3-(2-pyridyl)-1,2,4-triazole ligands with aliphatic substituents of different lengths and branching have been prepared and characterized together with the benzyl derivative with formula [Fe­(**L**)_3_]­[X]_2_·(solv)_*n*_ (**L** = 3-(2-pyridyl)-5*R*-1,2,4-triazole, *R* = Et (**L1**), *i*-Pr (**L2**), Me (**L3**), Bz (**L4**), c-Pr (**L5**) and C_5_H_9_O (**L6**), X = ClO_4_^−^ or BF_4_^−^, solv = H_2_O, EtOH or Me_2_CO). In the complexes with ethyl substituents [Fe­(**L1**)_3_]­[ClO_4_]_2_ (**1­[ClO**_**4**_ **]**_**2**_) and [Fe­(**L1**)_3_]­[BF_4_]_2_ (**1­[BF**_**4**_ **]**_**2**_), the length and flexibility of the alkyl chain are appropriate to obtain a structure without solvent molecules and strong intermolecular interactions (hydrogen bonds), leading to remarkable spin-crossover properties such as multistep abrupt spin transitions with thermal hysteresis associated with structural phase transitions, light or thermally induced excited spin state trapping effects (LIESST and TIESST, respectively) in **1­[BF**_**4**_ **]**_**2,**_ and LIESST and reverse LIESST in **1­[ClO**_**4**_ **]**_**2**_. The presence of disordered solvent molecules and, subsequently, weaker intermolecular interactions in the other compounds with shorter or bulkier substituents gives rise to gradual and incomplete spin-crossover, as usually found for these types of complexes. In [Fe­(**L2**)_3_]­[BF_4_]_2_·EtOH (**2­[BF**_**4**_ **]**_**2**_ **·EtOH**), removal of the solvent molecules to form the unsolvated compound [Fe­(**L2**)_3_]­[BF_4_]_2_ (**2­[BF**_**4**_ **]**_**2**_) results in more abrupt and complete spin-crossover.

## Graphical abstract

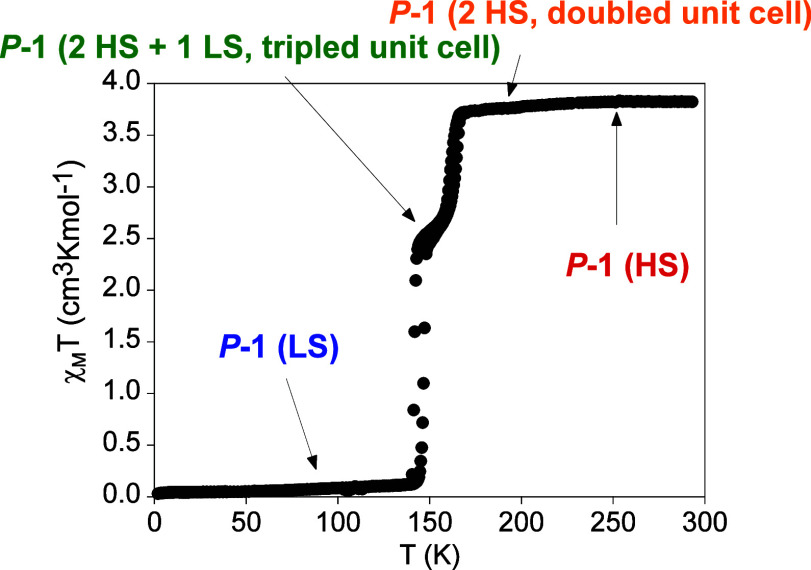

## Introduction

Spin crossover (SCO) complexes undergo an entropy-driven spin state change between the low-spin (LS) and high-spin (HS) states, which can be triggered by a variety of external stimuli (temperature, pressure, analytes, or electric fields).^1,2^ Since this phenomenon induces reversible changes of several physical properties, SCO materials have been proposed for applications in sensing, actuators, data storage, or spintronics.^3–6^ The most studied SCO systems are octahedral Fe­(II) complexes (3d^6^), which switch between the diamagnetic LS state (*S* = 0) and the paramagnetic HS state (*S* = 2). Indeed, the complexes with an FeN_6_ coordination sphere constitute the majority of the reported SCO complexes. Among them, triazole-based ligands have been extensively studied in SCO research since they afford many polynuclear Fe­(II) compounds displaying abrupt SCO with thermal hysteresis in the solid state, which is more convenient for many applications.^7–9^ By contrast, the mononuclear complexes of these ligands often exhibit gradual SCO without hysteresis.^10^ This has been attributed in 2-pyridyl-1,2,4-triazole-based ligands to the poor crystallinity/disorder of the structures, which results in residual HS fractions and gradual SCO with temperature due to the lack of strong intermolecular interactions.^7,8,11–15^

In this work, we have performed a systematic study of the SCO properties of Fe­(II) complexes of a family of ligands with new substituents at the 5 position of the triazole ring of bidentate 3-(2-pyridyl)-5*R*-1,2,4-triazole (see Scheme 1). These ligands have been used previously for the preparation of Cu­(II), Pd­(II), Pt­(II), and UO_2_ complexes for catalytic, fluorescence, and biomedical applications.^16–19^ To our knowledge, only the ligands with *R* = H and Me have been tested for SCO properties, but the use of bulkier or longer aliphatic substituents remains unexplored.^7,8,10–15^ Furthermore, we studied the effect of two different counteranions (ClO_4_^−^ or BF_4_^−^). Our results show that, as reported in the literature, many of these compounds are in the HS state or display gradual and incomplete spin transitions. However, we have found in complexes with substituents of intermediate size (ethyl and isopropyl) abrupt and complete spin transitions with temperature that can be also induced by irradiation at 10 K or by fast cooling in the light or thermally induced excited spin state trapping effects (LIESST and TIESST effects, respectively). Additionally, the spin transition can be reversed by changing the wavelength (reverse LIESST). In particular, the strong intermolecular interactions in the compounds with ethyl substituent together with the flexibility of the aliphatic chains and absence of solvent molecules induce a sharp multistep spin transition accompanied by several structural phase transitions.

**Scheme 1:**
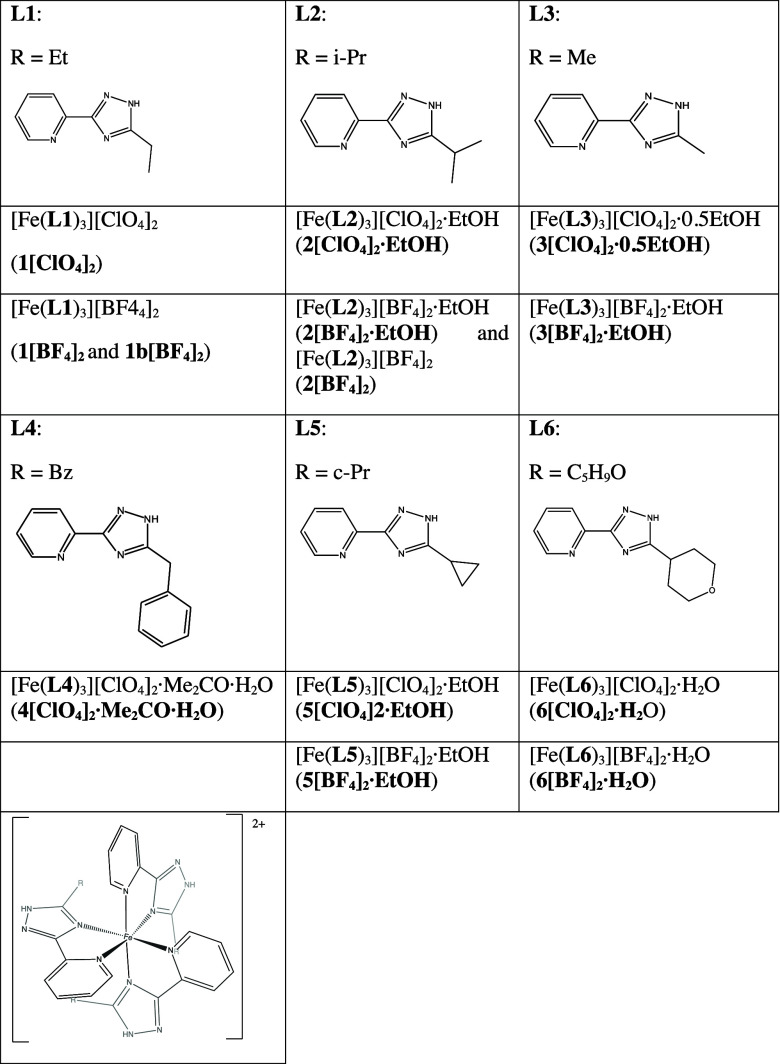
3-(2-Pyridyl)-5*R*-1,2,4-triazole Ligands and Compounds of This Work

The structure and magnetic properties of the compounds displaying abrupt and complete SCO {[Fe­(**L1**)_3_]­[ClO_4_]_2_ (**1­[ClO**_**4**_ **]**_**2**_), [Fe­(**L1**)_3_]­[BF_4_]_2_ (**1­[BF**_**4**_ **]**_**2**_) and [Fe­(**L2**)_3_]­[BF_4_]_2_ (**2­[BF**_**4**_ **]**_**2**_)} or interesting structural features {[Fe­(**L3**)_3_]­[ClO_4_]_2_·0.5EtOH (**3­[ClO**_**4**_ **]**_**2**_ **·0.5EtOH**)} are reported in the main text, while those of the remaining ones are described in the Supporting Information.

## Experimental Section

### Synthesis

The 3-(2-pyridyl)-5*R*-1,2,4-triazole ligands [*R* = Et (**L1**), *i*-Pr (**L2**), Me (**L3**), Bz (**L4**), c-Pr (**L5**), and C_5_H_9_O (**L6**), see Scheme 1] were prepared according to a literature procedure (see Supporting Information).^20^ Iron­(II) perchlorate hydrate, iron­(II) tetrafluoroborate hydrate, ethanol, acetone, and diethyl ether were obtained from commercial sources and used as received. **Caution!** Perchlorate salts are explosive when subjected to heat or friction; they must be handled with care.

Synthesis of complexes [Fe­(**L1**)_3_]­[ClO_4_]_2_ (**1­[ClO**_**4**_ **]**_**2**_), [Fe­(**L1**)_3_]­[BF_4_]_2_ (**1­[BF**_**4**_ **]**_**2**_ and **1b­[BF**_**4**_ **]**_**2**_), [Fe­(**L2**)_3_]­[ClO_4_]_2_·EtOH (**2­[ClO**_**4**_ **]**_**2**_ **·EtOH**), [Fe­(**L2**)_3_]­[BF_4_]_2_·EtOH (**2­[BF**_**4**_ **]**_**2**_ **·EtOH**), [Fe­(**L2**)_3_]­[BF_4_]_2_ (**2­[BF**_**4**_ **]**_**2**_), [Fe­(**L3**)_3_]­[ClO_4_]_2_·0.5EtOH (**3­[ClO**_**4**_ **]**_**2**_ **·0.5EtOH**), [Fe­(**L3**)_3_]­[BF_4_]_2_·EtOH (**3­[BF**_**4**_ **]**_**2**_ **·EtOH**), [Fe­(**L4**)_3_]­[ClO_4_]_2_·Me_2_CO·H_2_O (**4­[ClO**_**4**_ **]**_**2**_ **·Me**_**2**_ **CO·H**_**2**_ **O**), [Fe­(**L5**)_3_]­[ClO_4_]_2_·EtOH (**5­[ClO**_**4**_ **]**_**2**_ **·EtOH**), [Fe­(**L5**)_3_]­[BF_4_]_2_·EtOH (**5­[BF**_**4**_ **]**_**2**_ **·EtOH**), [Fe­(**L6**)_3_]­[ClO_4_]_2_·H_2_O (**6­[ClO**_**4**_ **]**_**2**_ **·H**_**2**_ **O**), and [Fe­(**L6**)_3_]­[BF_4_]_2_·H_2_O (**6­[BF**_**4**_ **]**_**2**_ **·H**_**2**_ **O**).

A solution of Fe­(ClO_4_)_2_·*n*H_2_O or [Fe­(BF_4_)_2_·*n*H_2_O] (0.06 mmol) in EtOH (1 mL) was added to a warm solution of the ligand (0.18 mmol) in 2 mL of EtOH. The resulting mixture was placed in the bottom of the layering tube, which was filled with diethyl ether and left for crystallization. **2­[BF**_**4**_ **]**_**2**_ was obtained by filtering the crystals of **2­[BF**_**4**_ **]**_**2**_ **·EtOH**. For **4­[ClO**_**4**_ **]**_**2**_ **·Me**_**2**_ **CO·H**_**2**_ **O**, the same synthetic method was used replacing EtOH by Me_2_CO. After a week, yellowish prismatic crystals were obtained. In some cases (**1­[ClO**_**4**_ **]**_**2**_, **1­[BF**_**4**_ **]**_**2**_ and **1b­[BF**_**4**_ **]**_**2**_, **2­[ClO**_**4**_ **]**_**2**_ **·EtOH**, **2­[BF**_**4**_ **]**_**2**_ **·EtOH**, **3­[ClO**_**4**_ **]**_**2**_ **·0.5EtOH**, **3­[BF**_**4**_ **]**_**2**_ **·EtOH** and **4­[ClO**_**4**_ **]**_**2**_ **·Me**_**2**_ **CO·H**_**2**_ **O**), inert atmosphere in a glovebox was required to obtain more reproducible and pure samples. On the contrary, the syntheses of **5­[ClO**_**4**_ **]**_**2**_ **·EtOH**, **5­[BF**_**4**_ **]**_**2**_ **·EtOH**, **6­[ClO**_**4**_ **]**_**2**_ **·H**_**2**_ **O,** and **6­[BF**_**4**_ **]**_**2**_ **·H**_**2**_ **O** did not need the use of inert atmosphere or ascorbic acid. For the **L1** ligand with BF_4_^−^, two different phases with the same [Fe­(**L1**)_3_]­(BF_4_)_2_ formula were obtained called **1­[BF**_**4**_ **]**_**2**_ and **1b­[BF**_**4**_ **]**_**2**_ (see below). In most cases, it was not possible to separate them. Due to this, elemental analysis was performed in a mixture of both phases that revealed the absorption of water molecules from air as observed in some of the other compounds. Anal. Calcd for Fe­(N_4_C_9_H_10_)_3_(ClO_4_)_2_ (**1­[ClO**_**4**_ **]**_**2**_): C, 41.72; H, 3.89; N, 21.62%. Found: C, 41.27; H, 3.83; N, 21.33%. Anal. Calcd for Fe­(N_4_C_9_H_10_)_3_(BF_4_)_2_·(H_2_O)_2_ (mixture of **1­[BF**_**4**_ **]**_**2**_ and **1b­[BF**_**4**_ **]**_**2**_): C, 41.15; H, 4.35; N, 21.33%. Found: C, 41.04; H, 4.03; N, 21.28%. Anal. Calcd for Fe­(N_4_C_10_H_12_)_3_(ClO_4_)_2_·H_2_O (**2­[ClO**_**4**_ **]**_**2**_ **·EtOH**): C, 43.03; H, 4.57; N, 20.07%. Found: C, 43.44; H, 4.41; N, 19.98%. Anal. Calcd for Fe­(N_4_C_10_H_12_)_3_(BF_4_)_2_·2H_2_O (**2­[BF**_**4**_ **]**_**2**_): C, 43.40; H, 4.86; N, 20.25%. Found: C, 43.39; H, 4.45; N, 20.12%. Anal. Calcd for Fe­(N_4_C_8_H_8_)_3_(ClO_4_)_2_ (**3­[ClO**_**4**_ **]**_**2**_ **·0.5EtOH**): C, 39.20; H, 3.29; N, 22.86%. Found: C, 39.21; H, 3.57; N, 22.09%. Anal. Calcd for Fe­(N_4_C_8_H_8_)_3_(BF_4_)_2_·1.5H_2_O (**3­[BF**_**4**_ **]**_**2**_ **·EtOH**): C, 39.11; H, 3.69; N, 22.81%. Found: C, 39.44; H, 3.65; N, 22.59%. Anal. Calcd for Fe­(N_4_C_14_H_12_)_3_(ClO_4_)_2_·Me_2_CO·H_2_O (**4­[ClO**_**4**_ **]**_**2**_ **·Me**_**2**_ **CO·H**_**2**_ **O**): C, 51.99; H, 4.27; N, 16.18%. Found: C, 51.85; H, 4.01; N, 16.35%. Anal. Calcd for Fe­(N_4_C_10_H_10_)_3_(ClO_4_)_2_ (**5­[ClO**_**4**_ **]**_**2**_ **·EtOH**): C, 44.30; H, 3.72; N, 20.66%. Found: C, 44.34; H, 3.86; N, 20.36%. Anal. Calcd for Fe­(N_4_C_10_H_10_)_3_(BF_4_)_2_·3H_2_O (**5­[BF**_**4**_ **]**_**2**_ **·EtOH**): C, 42.79; H, 4.31; N, 19.96%. Found: C, 42.91; H, 3.67; N, 19.93%. Anal. Calcd for Fe­(N_4_C_12_OH_14_)_3_(ClO_4_)_2_·H_2_O (**6­[ClO**_**4**_ **]**_**2**_ **·H**_**2**_ **O**): C, 44.87; H, 4.60; N, 17.44%. Found: C, 44.44; H, 4.55; N, 17.08%. Anal. Calcd for Fe­(N_4_C_12_OH_14_)_3_(BF_4_)_2_·H_2_O (**6­[BF**_**4**_ **]**_**2**_ **·H**_**2**_ **O**): C, 46.08; H, 4.73; N, 17.91%. Found: C, 45.93; H, 4.78; N, 17.34%.

Crystallographic data, structure refinements, and physical characterization are listed in the Supporting Information.

## Results and Discussion

### Synthesis

The family of 3-(2-pyridyl)-5*R*-1,2,4-triazole ligands was prepared by acylation of the corresponding hydrazides with picolinic acid iminoester formed in situ from 2-cyano pyridine and CH_3_ONa, followed by reflux of a methanolic solution of the obtained amidrazones (see scheme S1). Fe­(II) complexes of these ligands were prepared by slow diffusion of diethyl ether in EtOH (Me_2_CO in the case of **4­[ClO**_**4**_ **]**_**2**_ **·Me**_**2**_ **CO·H**_**2**_ **O**) solutions containing one equivalent of Fe^2+^ (BF_4_^−^ or ClO_4_^−^ salt) and three equivalents of the ligands. In the absence of an inert atmosphere, oxo-complexes of Fe­(III) were formed (**L2**), while the decomposition of ascorbic acid, which was added to avoid oxidation to Fe­(III), resulted in some cases in the formation of complexes containing oxalate (C_2_O_4_^2−^) bridging ligands (**L4**).^21^ The synthesis of the BF_4_^−^ salt of the [Fe­(**L1**)_3_]^2+^ complex resulted in the formation of a mixture of phases of identical formula [Fe­(**L1**)_3_]­(BF_4_)_2_ called **1­[BF**_**4**_ **]**_**2**_ and **1b­[BF**_**4**_ **]**_**2**_ being the structure of **1­[BF**_**4**_ **]**_**2**_ very similar to that of **1­[ClO**_**4**_ **]**_**2**_ (see below). The pure batches of both phases that could be obtained were identified by PXRD (Figure S10). These batches were used for magnetic characterization.

### Magnetic Properties of **1­[ClO**_**4**_ **]**_**2**_ and **1­[BF**_**4**_ **]**_**2**_

The thermal dependence of the product of the molar magnetic susceptibility with temperature (χ_M_ *T*) of a filtered sample of **1­[ClO**_**4**_ **]**_**2**_ at a scan rate of 2 K min^−1^ is shown in Figure 1. In the cooling mode, the χ_M_ *T* value remains constant at ca. 3.8 cm^3^ K mol^−1^ from 300 to 170 K, which is within the expected values for a HS Fe­(II). At lower temperatures, it shows an abrupt decrease in two steps. In the first one, χ_M_ *T* decreases to 2.7 cm^3^ K mol^−1^ at 157 K, which represents the conversion of around one-third of the Fe­(II) sites. This is followed by a plateau with χ_M_ *T* values in the 2.7–2.5 cm^3^ K mol^−1^ range from 157 to 145 K. At lower temperatures, there is the second abrupt decrease to reach values close to 0 cm^3^ K mol^−1^ at 140 K corresponding to a complete spin conversion. In the warming mode, the same two-step spin transition is maintained with small hysteresis loops in each step (5 and 3 K for the steps at lower and higher temperatures, respectively). χ_M_ *T* of **1­[BF**_**4**_ **]**_**2**_ follows a similar trend but shifted to lower temperatures and with broader hysteresis loops. Thus, at a scan rate of 0.5 K min^−1^, χ_M_ *T* decreases from 3.3 to 2.3 cm^3^ K mol^−1^ from 150 to 140 K in the first step, followed by a plateau in the 140−120 K temperature range and by the second abrupt decrease at lower temperatures to reach χ_M_ *T* values close to 0 cm^3^ K mol^−1^ below 110 K (see Figure 2). In the warming mode, the same two-step spin transition is maintained with hysteresis loops in each step (20 and 13 K for the steps at lower and higher temperatures, respectively). In contrast to **1­[ClO**_**4**_ **]**_**2**_, the thermal dependence of χ_M_ *T* of **1­[BF**_**4**_ **]**_**2**_ shows a strong dependence with the scan rate, which will be discussed below.

**Figure 1:**
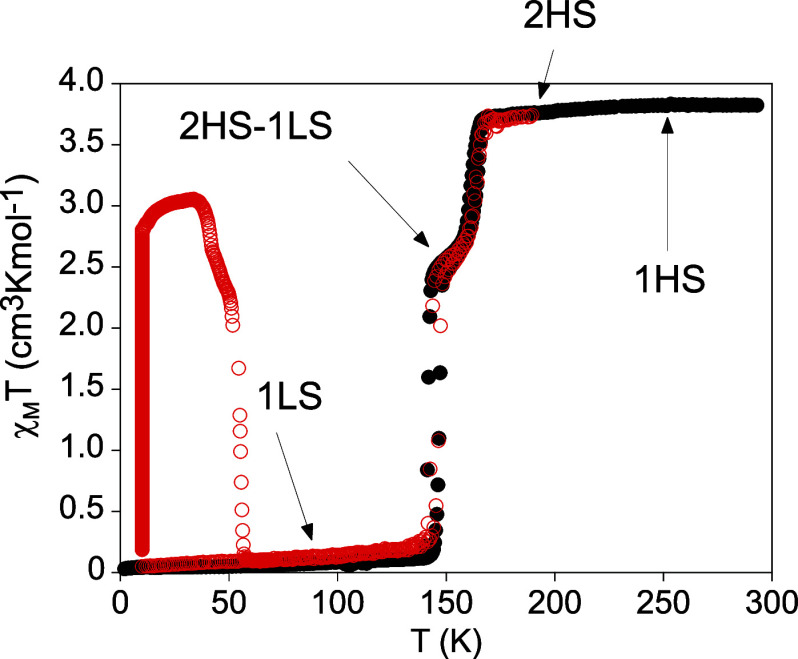
Thermal dependence of χ_M_ *T* of **1­[ClO**_**4**_ **]**_**2**_ at a scan rate of 2 K min^−1^. Full circles: data recorded without irradiation; empty red circles: data recorded after irradiation at 10 K.

**Figure 2:**
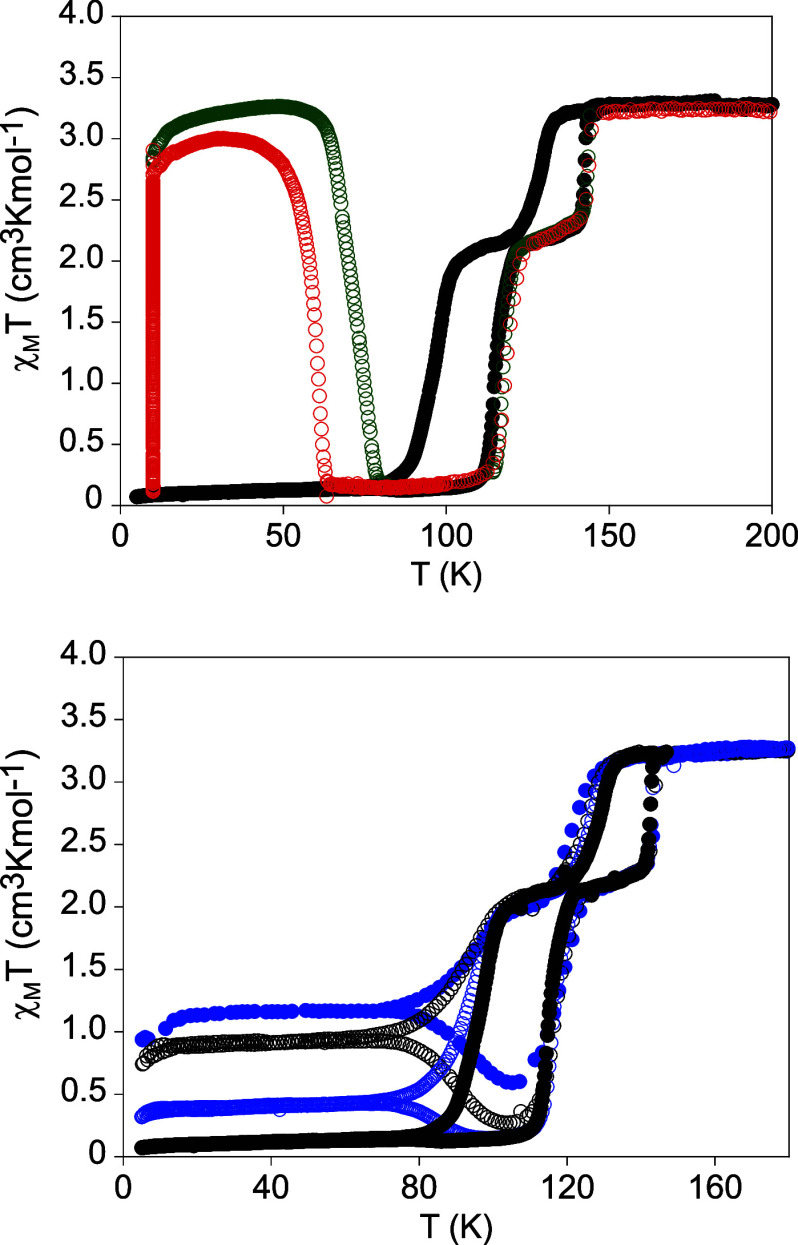
Thermal dependence of χ_M_ *T* of **1­[BF**_**4**_ **]**_**2**_ at a scan rate of 0.5 K min^−1^ (black circles) together with the TIESST curve (empty green circles) after fast cooling to 10 K and heating at a scan rate of 0.3 K min^−1^ and LIESST curve (empty red circles) after irradiation at 10 K and heating at a scan rate of 0.3 K min^−1^ (top). Thermal dependence of χ_M_ *T* of **1­[BF**_**4**_ **]**_**2**_. Scan rate of 0.5 K min^−1^ (full black circles), 1 K min^−1^ (empty blue circles), 2 K min^−1^ (empty black circles) and 4 K min^−1^ (full blue circles) (bottom).

### Structure of **1­[ClO**_**4**_ **]**_**2**_ and **1­[BF**_**4**_ **]**_**2**_

These compounds are formed by [Fe­(**L1**)_3_]^2+^ complexes and ClO_4_^−^ or BF_4_^−^ counteranions. As in the other compounds of this paper, Fe­(II) exhibits a distorted octahedral coordination to the three bidentate ligands, which binds through N of the pyridine ring and N4 of the triazole ring (Figure 3) in a *mer* configuration. The hydrogen bond formation in these structures suggests that only one tautomeric form of 1,2,4-triazole molecules with the NH group in N1 is observed (see below), in contrast to solution where ligands exist as mixture of all three possible prototropic forms.^22^

**Figure 3:**
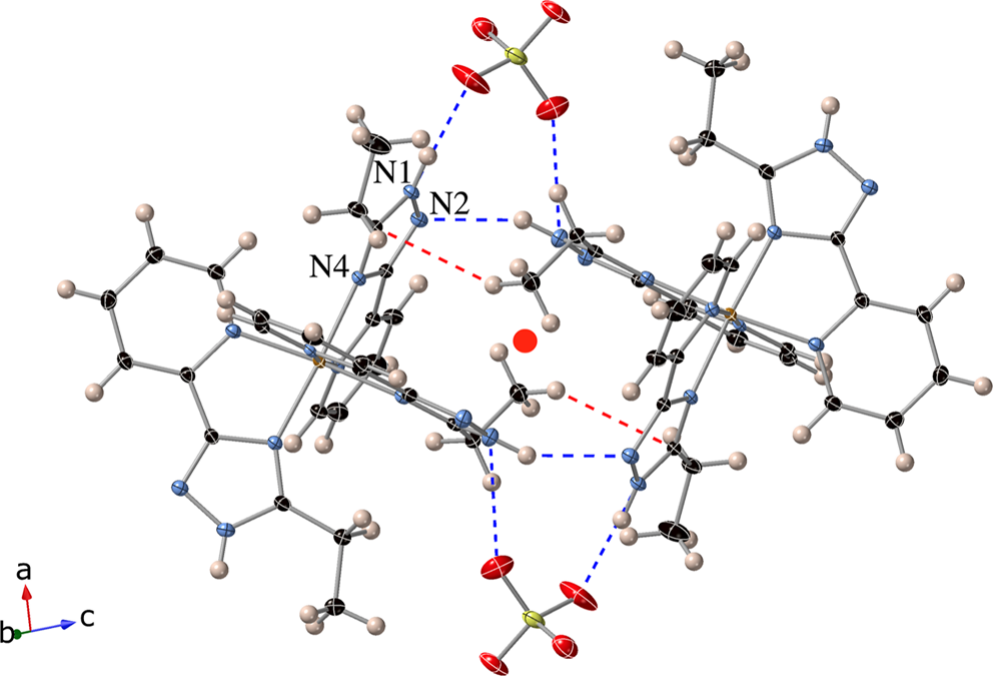
Structure of the dimers of [Fe­(**L1**)_3_]^2+^ complexes in the structure of **1­[ClO**_**4**_ **]**_**2**_ at 90 K. Hydrogen bonds (blue-dashed lines), CH···π interactions (red-dashed lines), red point (inversion center), Fe (orange), C (black), N (blue), O (red), Cl (yellow), and H (pink). Atom numbering refers to the type of N atom of the triazole ring.

Both compounds show structural phase transitions from 120 to 250 K. The structures of **1­[ClO**_**4**_ **]**_**2**_ at 90, 250, and 300 K present a similar unit cell with one crystallographically independent [Fe­(**L1**)_3_]^2+^ cation in the asymmetric unit with typical LS distances at 90 K (1LS phase) and typical HS ones above 250 K (1HS phase, see Tables 1 and S6). The two intermediate phases from 150 to 200 K retain the same triclinic *P*1̅ space but with approximately tripled (150 K) and doubled (180 and 200 K) unit cell volumes (see Table S2). These intermediate phases contain three (150 K) and two (180 and 200 K) crystallographically independent [Fe­(**L1**)_3_]^2+^ complexes in the asymmetric unit (see Figure 4). Fe–N bond lengths indicate that they correspond to a 2:1 mixture of HS and LS molecules (2HS-1S phase at 150 and 180 K) and to two HS molecules (2HS phase at 200 K) (Tables 1 and S6) in agreement with magnetic properties (see above). The crystallographic phase transition from the 2HS phase to the 1HS phase does not result in any modification of the magnetic properties, as the spin state of the complexes remains unaltered. In **1­[BF**_**4**_ **]**_**2**_, similar phases are obtained at lower temperatures. Thus, single crystal X-ray diffraction measurements in the warming mode indicate that the 1LS phase is obtained at 90 K, whereas the intermediate 2HS-1LS and 2HS phases are obtained at 120−140 and 150−200 K, respectively. Above 230 K, the low-temperature structure is recovered but with HS distances in the 1HS phase (see Figure S9). Fe–N distances of the structures confirm that the spin states of these phases are similar to those of **1­[ClO**_**4**_ **]**_**2**_ (see Tables 1 and S6). Therefore, these results confirm that the stepwise spin transition is coupled to structural phase transitions. Thus, the steps observed in the magnetic properties at around 140 and 160 K for **1­[ClO**_**4**_ **]**_**2**_ and 95 and 150 K (warming mode) for **1­[BF**_**4**_ **]**_**2**_ are associated with the following structural phase transitions: 1LS → 2HS-1LS → 2HS.

**Table 1:** Average Fe–N Distances (Å) and Σ and Θ Distortion Octahedral Parameters in the [Fe­(L^Et^)_3_]^2+^ Complexes of **1­[ClO**_**4**_ **]**_**2**_ and **1­[BF**_**4**_ **]**_**2**_ (See Table S6 for More Temperatures)

*T* (K) **1[ClO**_**4**_ **]**_**2**_	94(2)	151(2)	179.99(11)	300.6(10)
Fe1–N (Å)	1.9927(17)	2.004(4)	2.179(3)	2.191(3)
Fe2–N (Å)		2.179(4)	2.185(3)	
Fe3–N (Å)		2.180(4)		
Σ (deg) Fe1	51.4(3)	56.2(6)	82.0(3)	83.8(4)
Θ (deg) Fe1	110.3(4)	113.7(9)	199.3(6)	201.6(7)
Σ (deg) Fe2		81.2(5)	82.8(3)	
Θ (deg) Fe2		202.5(9)	199.6(6)	
Σ (deg) Fe3		84.4(5)		
Θ (deg) Fe3		198.9(9)		

*T* (K) **1[BF**_**4**_ **]**_**2**_	99.9(5)	119.98(16)	149.98(10)	299.99(10)
Fe1–N (Å)	1.994(4)	2.010(4)	2.181(4)	2.188(4)
Fe2–N (Å)		2.167(4)	2.186(4)	
Fe3–N (Å)		2.174(4)		
Σ (deg) Fe1	50.2(6)	53.2(6)	82.3(5)	82.2(5)
Θ (deg) Fe1	113.1(11)	117.4(9)	204.4(9)	200.9(10)
Σ (deg) Fe2		79.0(5)	83.2(6)	
Θ (deg) Fe2		199.7(9)	202.8(10)	
Σ (deg) Fe3		81.8(5)		
Θ (deg) Fe3		197.3(9)		

**Figure 4:**
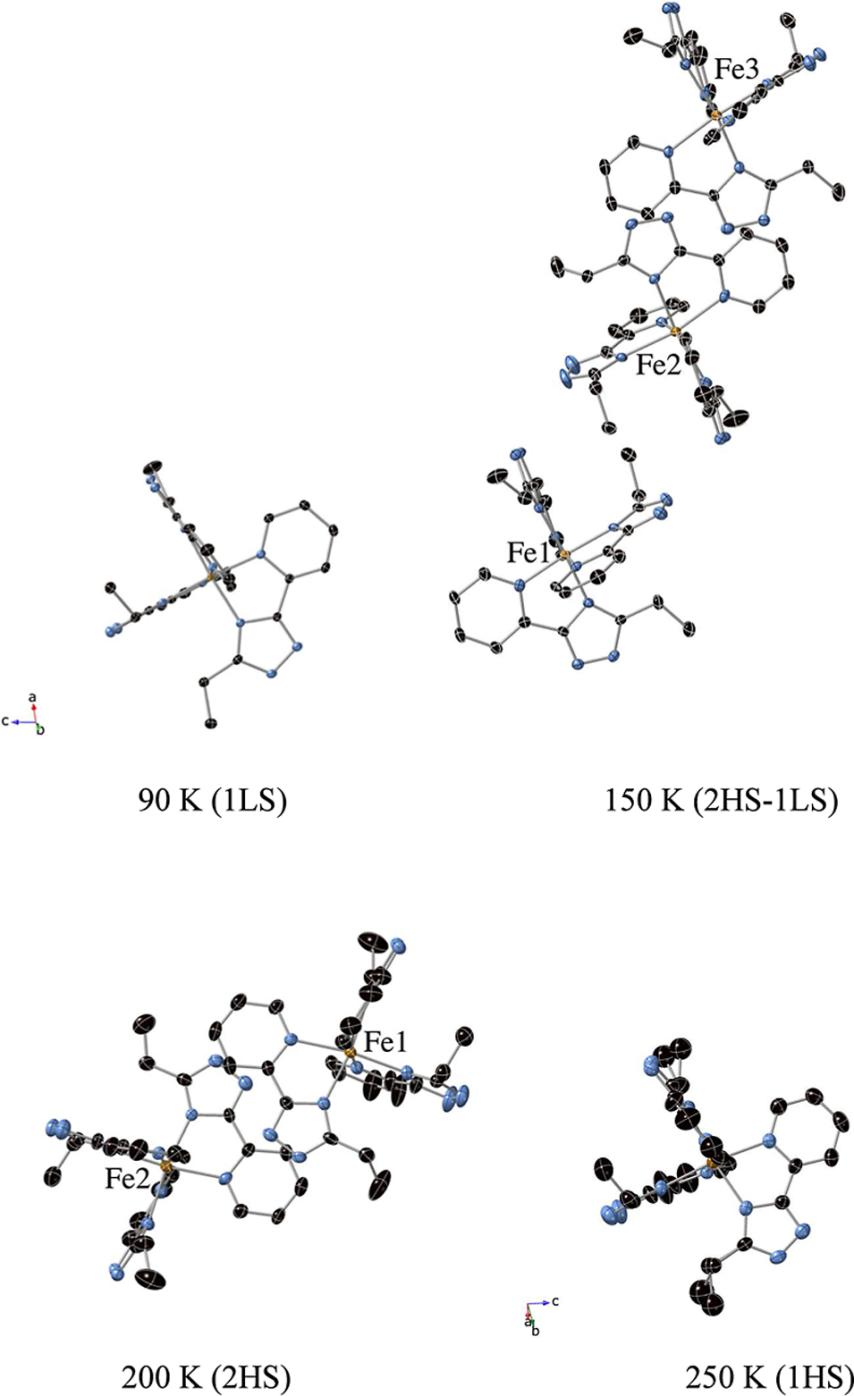
[Fe­(**L1**)_3_]^2+^ complexes in the structure of **1­[ClO**_**4**_ **]**_**2**_ at 90, 150, 200, and 250 K. Fe (orange), C (black), and N (blue).

### Structural Phase Transitions of **1­[ClO**_**4**_ **]**_**2**_ and **1­[BF**_**4**_ **]**_**2**_

The 1HS to 2HS conversion can be considered as cell-doubling symmetry breaking involving one of the three unit cell parameters. To describe the group/subgroup relationship between 1HS and 2HS phases, we have used another lattice common to both phases, as shown in Figure S12. As mentioned above, this phase transition does not affect the spin state of the compound. In contrast to this, the conversion between the 2HS and 2HS-1LS phases does not imply a group/subgroup relationship and it can be considered then as a reconstructive transition, which changes the spin state of the compound.^23^ Finally, the phase transition to the 1LS phase represents the recovery of high temperature 1HS structure and is a re-entrant phase transition.^23^ Many compounds displaying multistep spin transitions coupled to structural phase transitions have been reported in the literature^24–27^ associated with Jahn−Teller distortion, ferroelastic phase transition,^28^ and/or spin-state ordering.^29^ In most of them, intermediate crystal phases of lower symmetry containing HS and LS mixtures lead to the fully LS or HS states at lower or higher temperatures, respectively.^30^ However, the combination of re-entrant and reconstructive phase transitions found in **1­[ClO**_**4**_ **]**_**2**_ and **1­[BF**_**4**_ **]** is unusual.

To understand why such interesting and rare behavior has been obtained in these two compounds, we compared their structures and crystal packing with those of the remaining compounds of this work obtained with similar triazole ligands and shorter or bulkier R substituents. The most important structural factors that could explain the remarkable SCO properties of **1­[ClO**_**4**_ **]**_**2**_ and **1­[BF**_**4**_ **]** are the strong intermolecular interactions between the Fe­(II) complexes and the absence of solvent molecules in the structure. Thus, the gradual and incomplete SCO usually found for this type of complex has been attributed to the presence of solvent molecules in the structure with highly disordered areas, including the anions and/or unresolved solvent molecules. The poor crystallinity/disorder is related to the observed residual HS fractions at low temperatures. In addition, these highly disordered areas result in isolated SCO complexes, which explains the gradual character of the SCO of these compounds.^8^

We start with the role played by intermolecular interactions. In contrast to the other compounds of this work that only present hydrogen bonds between the NH groups of triazole and ClO_4_^−^ or BF_4_^−^ counteranions or solvent molecules, **1­[ClO**_**4**_ **]**_**2**_ and **1­[BF**_**4**_ **]** display hydrogen bonds between [Fe­(**L1**)_3_]^2+^ complexes. Thus, in the 1LS and 1HS phases, dimers of these complexes interact through two hydrogen bonds between NH and N from triazole. These interactions involve two of the three **L1** ligands from each complex and could be considered as a bifurcated hydrogen bond since the NH group interacts with two donors: the already mentioned N from the triazole and O or F from ClO_4_^−^ or BF_4_^−^ counteranion (see Figures 3, S5, and S8). The two of the three NH groups not involved in these interactions form a hydrogen bond with a ClO_4_^−^ or BF_4_^−^ counteranion. In addition, the [Fe­(**L1**)_3_]^2+^ complexes of these dimers are linked through CH···π interactions between triazole rings and CH_3_ groups at 90 and 120 K for **1­[ClO**_**4**_ **]**_**2**_ and 100 K for **1­[BF**_**4**_ **]** (see Figures 3, S5, and S8 and associated text in the Supporting Information for more information about the packing). There is an inversion center in the centers of these dimers.

Remarkable changes in the organization of these dimers appear in the intermediate 2HS-1LS and 2HS phases. Thus, in the 2HS-1LS phase obtained at 150 K for **1­[ClO**_**4**_ **]**_**2**_ and 120 K for **1­[BF**_**4**_ **]**_**2**_, two types of dimers between the three crystallographically independent [Fe­(**L1**)_3_]^2+^ complexes, called Fe1 (LS), Fe2 (HS) and Fe3 (HS), are observed to be formed by Fe1–Fe2 and Fe3–Fe3 complexes in the following order Fe1–Fe2–Fe3–Fe3–Fe2–Fe1 (see Figure 5). The intradimer interactions in LS/HS Fe1–Fe2 dimers are weakened with respect to those of HS/HS Fe3–Fe3 dimers. Thus, in Fe3–Fe3 dimers, there are two equal NH···N distances, which in Fe1–Fe2 dimers become nonequivalent and longer, especially one of them (2.165 and 2.488 Å for Fe1–Fe2 and 2.103 Å for Fe3–Fe3 in **1­[ClO**_**4**_ **]**_**2**_ and 2.188 and 2.509 Å for Fe1–Fe2 and 2.105 Å for Fe3–Fe3 in **1­[ClO**_**4**_ **]**_**2**_). Moreover, the two CH···π interactions between triazole rings and CH_3_ groups are not observed in Fe1–Fe2 dimers. Another difference is that the inversion center between the two complexes found in Fe3–Fe3 dimers and those of the 1LS and 1HS phases is lost in the Fe1–Fe2 dimers (Figure 5). These changes in intradimer interactions could be responsible of the change in elastic interactions leading to [LS−HS−HS−HS−HS−LS] ordering and long-range spin-state concentration wave.^31^ In the 2HS phase obtained at 180 and 200 K in **1­[ClO**_**4**_ **]**_**2**_ and 150 K in **1­[BF**_**4**_ **]**_**2**_, the two crystallographically independent HS [Fe­(**L1**)_3_]^2+^ complexes in the asymmetric unit (Fe1 and Fe2 complexes) form Fe1–Fe1 and Fe2–Fe2 dimers linked through two NH···N hydrogen bonds, as observed at lower temperatures. The NH···N distances are slightly different (2.265 Å for Fe1–Fe1 dimers and 2.161 Å for Fe2–Fe2 dimers in **1­[ClO**_**4**_ **]**_**2**_ and 2.345 Å for Fe1–Fe1 dimers and 2.134 Å for Fe2–Fe2 dimers in **1­[BF**_**4**_ **]**_**2**_) but not so long as those observed in the LS/HS dimers at lower temperatures. Furthermore, the dimers of hydrogen-bonded [Fe­(**L1**)_3_]^2+^ complexes in this 2HS phase are related by an inversion center. We conclude that the disruption and changes of these hydrogen bond interactions in the intermediate 2HS-1LS and 2HS phases are associated with the structural phase transitions.

**Figure 5:**
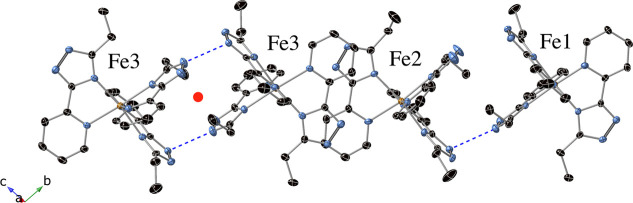
Fe3–Fe3 and Fe1–Fe2 dimers of [Fe­(**1**)_3_]^2+^ complexes in the structure of **1­[ClO**_**4**_ **]**_**2**_ at 150 K (2HS-1LS phase). Hydrogen bonds (blue-dashed lines), red point (inversion center), Fe (orange), C (black), and N (blue).

To illustrate the importance of hydrogen bond formation, the structure of the second phase obtained with the BF_4_^−^ salt (**1b­[BF**_**4**_ **]**_**2**_) is shown in the Supporting Information. The [Fe­(**L1**)_3_]^2+^ complexes of this phase remain in the HS state from 90 to 300 K. In contrast to **1­[BF**_**4**_ **]**_**2**_, the three NH groups of the triazole groups in **1b­[BF**_**4**_ **]**_**2**_ form hydrogen bonds with BF_4_^−^ counteranions (see Figure S11). Therefore, hydrogen-bonded dimers of the complexes are not observed. This confirms that the interesting SCO properties of **1­[ClO**_**4**_ **]**_**2**_ and **1­[BF**_**4**_ **]**_**2**_ are related to the presence of hydrogen-bonding between the complexes, in contrast to the isolation of the complexes observed in **1b­[BF**_**4**_ **]**_**2**_ and the other structures of this work, in which spin transition is not observed. Regarding the role of the absence of solvent molecules in the interesting SCO properties of **1­[ClO**_**4**_ **]**_**2**_ and **1­[BF**_**4**_ **]**_**2**_, we conclude that it is not the most crucial factor since the structure of **1b­[BF**_**4**_ **]**_**2**_ does not present solvent molecules in the structure, and this compound remains in the HS state in the 5−300 K temperature range. This indicates that the first factor, the presence of hydrogen-bonded complexes, is the most determinant one.

We think that the presence of different phases in these structures is related to the flexibility of the alkyl chains. Thus, their disposition changes with the temperature for **1­[ClO**_**4**_ **]**_**2**_ and **1­[BF**_**4**_ **]**_**2**_ (Figures 4 and S9). In the 1LS phases at 90 K in **1­[ClO**_**4**_ **]**_**2**_ and 100 K in **1­[BF**_**4**_ **]**_**2**_, two of the three ethyl groups lie close to the plane of the triazole ring, while in the third one, the terminal CH_3_ of the ethyl group is clearly out of this plane. In the 2HS-1LS phases (150 K in **1­[ClO**_**4**_ **]**_**2**_ and 120 K in **1­[BF**_**4**_ **]**_**2**_), Fe1 LS complexes show the same disposition (two parallel and one perpendicular), while the HS Fe2 and Fe3 complexes present two of the three ethyl groups clearly out of the plane defined by the triazole ring. In the 2HS phases at 200 K (**1­[ClO**_**4**_ **]**_**2**_ **)** and 150 K (**1­[BF**_**4**_ **]**_**2**_), the two crystallographically independent HS [Fe­(**L1**)_3_]^2+^ complexes again show only one parallel ethyl group. In the 1HS phases at 250 and 300 K (**1­[ClO**_**4**_ **]**_**2**_ **)** and 230 and 300 K (**1­[BF**_**4**_ **]**_**2**_), the crystallographically independent HS complex shows one ethyl chain lying out of the plane defined by the triazole ring (perpendicular), while the other two are disordered. Therefore, the disposition of the alkyl chains in the intermediate phases changes and could play a role in the different intermolecular interactions between neighboring complexes and the structural phase transitions. In agreement with this, the three ethyl chains of the crystallographically HS [Fe­(**L1**)_3_]^2+^ complex of **1b­[BF**_**4**_ **]**_**2**_ from 90 to 300 K show a similar disposition (out of the plane defined by the triazole ring, see Figure S11).

Finally, the shift to lower temperatures of the spin transition in the BF_4_^−^ salt can be explained by the greater withdrawal of electronic density for this counteranion compared with ClO_4_^−^. In this case strong H···F hydrogen bonds of two of the three NH groups of the complex reduce the electron density onto the lone pair of the nitrogen donor by inductive effects.^32^ As a result, the iron–nitrogen bond is weakened and the *T*_1/2_ is decreased as observed experimentally.

### TIESST and LIESST Effects of **1­[ClO**_**4**_ **]**_**2**_ and **1­[BF**_**4**_ **]**_**2**_

Having established that the two-step SCO was coupled to a structural phase transition, different scan rates and light were used to further tune the spin states of these compounds. Thermal dependence of χ_M_ *T* of **1­[BF**_**4**_ **]**_**2**_ shows important changes with the scan rate. When the scan rate is increased to 4 K min^−1^ on cooling, the abrupt two-step decrease is observed but shifted to lower temperatures and with a residual fraction of HS molecules after the second decrease below 70 K with χ_M_ *T* values of 1.2 cm^3^ K mol^−1^. In the heating mode, the same residual HS fraction is obtained below 70 K, but χ_M_ *T* decreases gradually at higher temperatures to reach a minimum value of 0.6 cm^3^ K mol^−1^ at 106 K following the abrupt two-step increase due to the spin transition. This profile is characteristic of the TIESST effect. It arises when the spin transition is kinetically slow in the time scale of the measurement and occurs typically in compounds exhibiting spin transition below 100 K as **1­[BF**_**4**_ **]**_**2**_.^33–35^ In addition, the large elastic energy of the change in lattice symmetry, associated with the reconstructive phase transition observed in this compound, can also contribute to this phenomenon. The kinetic origin of this effect is proved by the intermediate behaviors observed at scan rates of 2 and 1 K min^−1^ and the disappearance of the effect at a scan rate of 0.5 K min^−1^ (see Figure 2). To determine the *T*(TIESST) values, crystals of **1­[BF**_**4**_ **]**_**2**_ were rapidly quenched at 10 K by inserting them in less than 10 s from room temperature down to the cavity of the SQUID previously placed at 10 K. After thermal stabilization, the temperature was then increased at 0.3 K min^−1^ and the minimum of the δχ_M_ *T*/δ*T* versus *T* plots gave the *T*(TIESST) value.^35^ Remarkably, two maxima can be distinguished with values of 68 and 75 K. This could indicate the presence of phase transitions (Figure S27). Crystallographic measurements at lower temperatures are needed to clarify this.

Photomagnetic studies on **1­[ClO**_**4**_ **]**_**2**_ **and 1­[BF**_**4**_ **]**_**2**_ show that irradiation at 660 nm at 10 K causes an increase in χ_M_ *T* (Figures 1 and 2). After a very long irradiation time of ca. 18 h for **1­[ClO**_**4**_ **]**_**2**_ and 6 h for **1­[BF**_**4**_ **]**_**2**_ the χ_M_ *T* value is saturated to the maximum value. Then, the irradiation was switched off and the temperature was increased at a rate of 0.3 K min^−1^, reaching a maximum value close to 3.0 cm^3^ K mol^−1^ at 30 K, which indicates a photoconversion efficiency of ca. 80%. Interestingly, the abrupt decrease of the LIESST curve above 37 K in **1­[ClO**_**4**_ **]**_**2**_ with two steps could suggest the presence of crystallographic phase transitions after photoexcitation (see Figure 1), as observed in Fe­(II)^36,37^ and Fe­(III)^29^ complexes of tridentate ligands. Thus, δχ_M_ *T*/δ*T* vs *T* reveals the presence of two minima at 42 and 55 K (Figure S27). These two *T*(LIESST) spectra could indicate the temperature of these structural phase transitions. In **1­[BF**_**4**_ **]**_**2**_, only one *T*(LIESST) of 60 K was observed. The reverse LIESST effect could also be detected in **1­[ClO**_**4**_ **]**_**2**_ over very long times. Thus, crystals of **1­[ClO**_**4**_ **]**_**2**_ were first irradiated at 10 K with 660 nm for 20 h reaching a χ_M_ *T* value of 3.4 cm^3^ K mol^−1^. This was followed by a period of 3 h without irradiation, which showed a negligible decrease of χ_M_ *T* to 3.3 cm^3^ K mol^−1^, and then by irradiation with 808 nm, which caused a more abrupt decrease of χ_M_ *T* to reach a residual 0.4 cm^3^ K mol^−1^ value after another 22 h. The reversibility of the process was proved by subsequent irradiation with 660 nm for 5 h, which led to a final χ_M_ *T* value of 3.3 cm^3^ K mol^−1^ and the recovery of the HS state (see Figure S28).

Again, the most interesting photomagnetic behavior of these compounds is found for **1­[ClO**_**4**_ **]**_**2**_, as the steps in the LIESST curve suggest the presence of phase transitions after light irradiation. Confirmation of this would open a very promising possibility to access these intermediate photoexcited states by changing the wavelength, irradiation time, or temperature, in view of the reverse LIESST effect shown by this compound. In the case of **1­[BF**_**4**_ **]**_**2**_, new intermediate LS/HS states could also be obtained by changing the temperature on quickly cooled samples to 10 K, as a result of the TIESST effect shown by this compound. A more complete photomagnetic and photostructural characterization is planned for this study.

### Structures of **2­[ClO**_**4**_ **]**_**2**_ **·EtOH** and **2­[BF**_**4**_ **]**_**2**_ **·EtOH**

The increase of the size of the substituent from ethyl to isopropyl in [Fe­(**L2**)_3_]^2+^ complexes gives rise to compounds in which the spin transition properties are lost. **2­[ClO**_**4**_ **]**_**2**_ **·EtOH** and **2­[BF**_**4**_ **]**_**2**_ **·EtOH** crystallize in the *P*1̅ space group and contain one crystallographically independent [Fe­(**L2**)_3_]^2+^ complex in the asymmetric unit with typical HS Fe–N bond lengths (average = 2.182(5) Å for **2­[ClO**_**4**_ **]**_**2**_ **·EtOH** and 2.186(4) Å for **2­[BF**_**4**_ **]**_**2**_ **·EtOH** at 120 K), in agreement with magnetic properties (see Supporting Information), two crystallographically independent ClO_4_^−^ or BF_4_^−^ counteranions and disordered EtOH solvent molecules (see Figure 6). The three NH groups of [Fe­(**L2**)_3_]^2+^ complexes form hydrogen bonds with ClO_4_^−^ or BF_4_^−^ counteranions (see Figures S13 and S14). The packing of the complexes shares some type of intermolecular interactions with the structures of **1­[ClO**_**4**_ **]**_**2**_ and **1­[BF**_**4**_ **]**_**2**_ such as short contacts between CH groups from pyridine and N from triazole, CH···π contacts between pyridine rings or CH···π contacts between pyridine rings and CH_3_ group but, in contrast to **1­[ClO**_**4**_ **]**_**2**_ and **1­[BF**_**4**_ **]**_**2**_, hydrogen bonds interactions between the complexes are not observed. This lack of strong intermolecular interactions and the presence of disordered EtOH in the structure could explain the isolation of the complexes, the gradual and incomplete spin transition found in **1­[ClO**_**4**_ **]**_**2**_ and the HS found in **1­[BF**_**4**_ **]**_**2**_ in contact with the mother liquor (see below).

**Figure 6:**
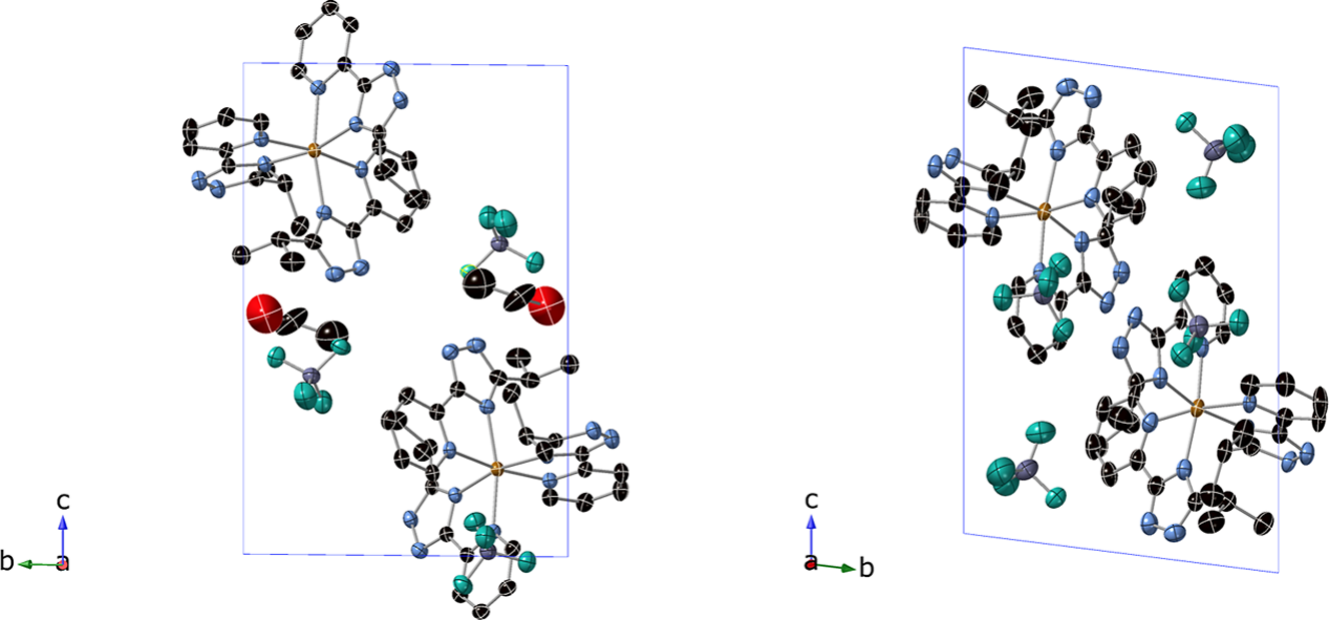
Projection of the structure in the bc plane of **2­[BF**_**4**_ **]**_**2**_ **·EtOH** at 120 K (left) and of **2­[BF**_**4**_ **]**_**2**_ at 100 K (left). For clarity, only half of the disordered EtOH molecules of **2­[BF**_**4**_ **]**_**2**_ **·EtOH** is shown. Fe (orange) C (black), N (blue), B (gray), and F (green).

The PXRD pattern of the two compounds in contact with the mother liquor shows a good agreement with the simulated one from single crystal X-ray diffraction. However, the PXRD pattern of the filtered samples shows some differences that suggests important structural changes that have drastic effects in the magnetic properties of **2­[BF**_**4**_ **]**_**2**_ **·EtOH** (see Figures S15 and S16). Due to this, we determined the structure of filtered crystals of **2­[BF**_**4**_ **]**_**2**_ **·EtOH** called **2­[BF**_**4**_ **]**_**2**_, which lose the EtOH solvent molecule and keep the crystallinity after filtering.

### Structure of **2­[BF**_**4**_ **]**_**2**_

Despite the loss of crystallinity after filtering the crystals, the structure of **2­[BF**_**4**_ **]**_**2**_ could be solved at 100 and 300 K. The unit cell presents several changes with respect to that of **2­[BF**_**4**_ **]**_**2**_ **·EtOH** measured at 120 K in a crystal directly taken from the mother liquor described in the previous paragraph. The most important one is the absence of solvent molecules (Figure 6). Fe–N bond lengths [average = 2.153(13) Å at 100 K and 2.196(7) Å at 300 K] are typical HS distances, but the lower distances at 100 K, although closer to HS values, suggest a partial spin transition at this temperature. This is reflected in the SCO curve at lower temperatures shown by magnetic measurement (see below). Single crystal X-ray diffraction measurements at lower temperatures should be performed to access to the structure of the full LS state. This shortening of the distances is not observed in solvated **2­[BF**_**4**_ **]**_**2**_ **·EtOH** as expected for the HS behavior found experimentally (see below). The PXRD of the filtered crystals shows good agreement with the simulated one from the single crystal structure of **2­[BF**_**4**_ **]**_**2**_ (see Figure S16). Elemental analysis of the filtered crystals confirms the loss of the EtOH molecules found in the structure of **2­[BF**_**4**_ **]**_**2**_ **·EtOH** and the absorption of water moisture molecules (see the Experimental Section).

### Magnetic Properties of **2­[BF**_**4**_ **]**_**2**_

The magnetic measurements of **2­[BF**_**4**_ **]**_**2**_ **·EtOH** in contact with the mother liquor show typical HS χ_M_ *T* values close to 3.8 cm^3^ K mol^−1^ above 100 K and a small decrease to 3.3 cm^3^ K mol^−1^ at 50 K, which could be attributed to partial SCO, followed by a more abrupt decrease due to zero-field-splitting of Fe­(II) (see Figure 7). In the filtered sample, **2­[BF**_**4**_ **]**_**2**_, the decrease of χ_M_ *T* below 130 K is more abrupt and complete, reaching 0.7 cm^3^ K mol^−1^ at 60 K. Therefore, **2­[BF**_**4**_ **]**_**2**_ shows an almost complete spin transition in the 60−130 K temperature range. The SCO becomes more complete after heating to 400 K, with a similar behavior but a lower residual HS fraction at low temperatures (0.4 cm^3^ K mol^−1^ below 50 K). As mentioned in the structural description, the removal of the disordered EtOH molecules in **2­[BF**_**4**_ **]**_**2**_ **·EtOH** by filtration leads to the stabilization of the LS state at low temperatures and to a low *T*_1/2_ of 85 K (*T*_1/2_ temperature of 50% LS/HS). This may be related to the increased chemical pressure associated with the loss of the solvent molecules in **2­[BF**_**4**_ **]**_**2**_, which occupy a cavity surrounded by [Fe­(**L2**)_3_]^2+^ complexes in **2­[BF**_**4**_ **]**_**2**_ **·EtOH** (see above). However, the absence of strong intermolecular interactions such as the hydrogen bonds found in the structures of **1­[ClO**_**4**_ **]**_**2**_ and **1­[BF**_**4**_ **]**_**2**_ gives rise to a less abrupt and complete SCO than that found in these compounds, confirming the important role of hydrogen bonding in the SCO properties of this family of compounds. **2­[BF**_**4**_ **]**_**2**_ shows a clear LIESST effect with a complete photoconversion after photoexcitation at 660 nm and an abrupt LIESST curve with a *T*(LIESST) of 43 K (see Figure 7).

**Figure 7:**
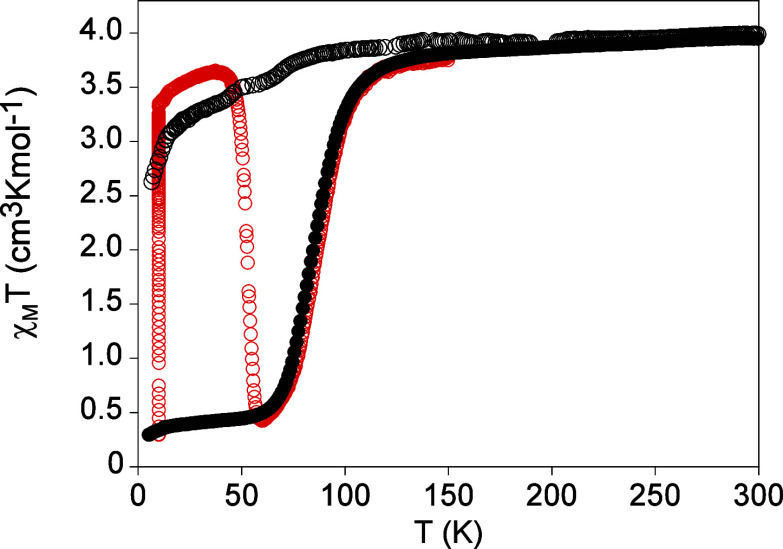
Thermal dependence of χ_M_ *T* of **2­[BF**_**4**_ **]**_**2**_ previously heated at 400 K in the squid (full black circles) and **2­[BF**_**4**_ **]**_**2**_ **·EtOH** measured in contact with the mother liquor (empty black circles). Empty red circles: data of **2­[BF**_**4**_ **]**_**2**_ recorded after irradiation at 10 K.

Finally, the photomagnetic properties of the other compounds of this article, which present gradual and incomplete SCO, are shown in the Supporting Information. In all cases, the LIESST effect is observed with relatively low *T*(LIESST) values of around 50−60 K close to that found for **1­[ClO**_**4**_ **]**_**2**_, **1­[BF**_**4**_ **]**_**2,**_ and **2­[BF**_**4**_ **]**_**2**_. This is a similar behavior to that of the other Fe­(II) complexes with bidentate ligands.^38^ With a few exceptions,^39^ the LIESST effect has not been studied for complexes of related triazole-based ligands.

### Structure of **3­[ClO**_**4**_ **]**_**2**_ **·0.5EtOH**

The decrease in size from the ethyl to methyl substituent in the **L3** ligand leads to compounds not displaying abrupt and complete SCO properties. However, in the case of the ClO_4_^−^ salt of [Fe­(**L3**)_3_]^2+^ **(3­[ClO**_**4**_ **]**_**2**_ **·0.5EtOH),** remarkable structural properties are observed. It crystallizes in the monoclinic *P*2_1_/*n* space group. The asymmetric unit displays two crystallographically independent [Fe­(**L3**)_3_]^2+^ complexes with Fe1 and Fe2, four ClO_4_^−^ counteranions with a disorder in one of them, and one disordered EtOH molecule. One of two [Fe­(**L3**)_3_]^2+^ complexes with Fe1 presents a disorder in one of the three **L3** ligands, which was solved with two possible configurations with occupancies close to 50%. Interestingly, these two configurations correspond to a *fac* isomer and a *mer* isomer (see Figure 8). Therefore, around 25% of the complexes of this structure are in a *fac* configuration. This is unusual as the most common binding mode for this type of complexes and in general for Fe­(II) complexes of asymmetric heterocyclic chelates^14^ is the *mer* configuration since it minimizes interligand repulsions resulting from the substituent groups.^13^ The presence of a small fraction of different isomers has been suggested in the literature by Mössbauer data of unsubstituted 3-(pyridin-2-yl)-1,2,4-triazole but, to our knowledge, there are not previous structural evidence.^10,12^ A possible explanation is that in this compound, interligand repulsions are less important than in the other compounds in this article due to the relatively small size of the methyl substituent. Furthermore, one of the two configurations of the disordered ClO_4_^−^, which forms a hydrogen bond with the *fac* isomer, could contribute to the stabilization of this isomer. The Fe–N bond lengths of the complexes of this structure at 120 K are indicative of the HS state [average Fe–N distances for Fe1 and Fe2 of 2.195(9) and 2.189(5) Å, respectively] in agreement with magnetic properties (see Supporting Information). As the other compounds of this paper did not show abrupt and complete SCO, the NH groups of **L3** ligands form hydrogen bonds with ClO_4_^−^ counteranions (Figure S17). On the other hand, neighboring [Fe­(**L3**)_3_]^2+^ complexes are quite isolated due to the presence of disordered EtOH solvent molecules. Thus, they interact through weak interactions between CH and CH_3_ groups and triazole or pyridine rings. The PXRD pattern of a filtered sample of this compound is consistent with the single crystal structure (Figure S19). The structure of the BF_4_^−^ salt of the same complex of formula [Fe­(**L3**)_3_]­[BF_4_]_2_·EtOH (**3­[BF**_**4**_ **]**_**2**_ **·0.5EtOH**) changes drastically, as described in the Supporting Information. In this case, there is only one crystallographically independent [Fe­(**L3**)_3_]^2+^ complex with typical HS distances As in **3­[ClO**_**4**_ **]**_**2**_ **·0.5EtOH**, there is a disorder of one of the three **L3** ligands, which was solved with two possible configurations with occupancies of 0.88 and 0.12. In this case, both of them correspond to a *mer* configuration.

**Figure 8:**
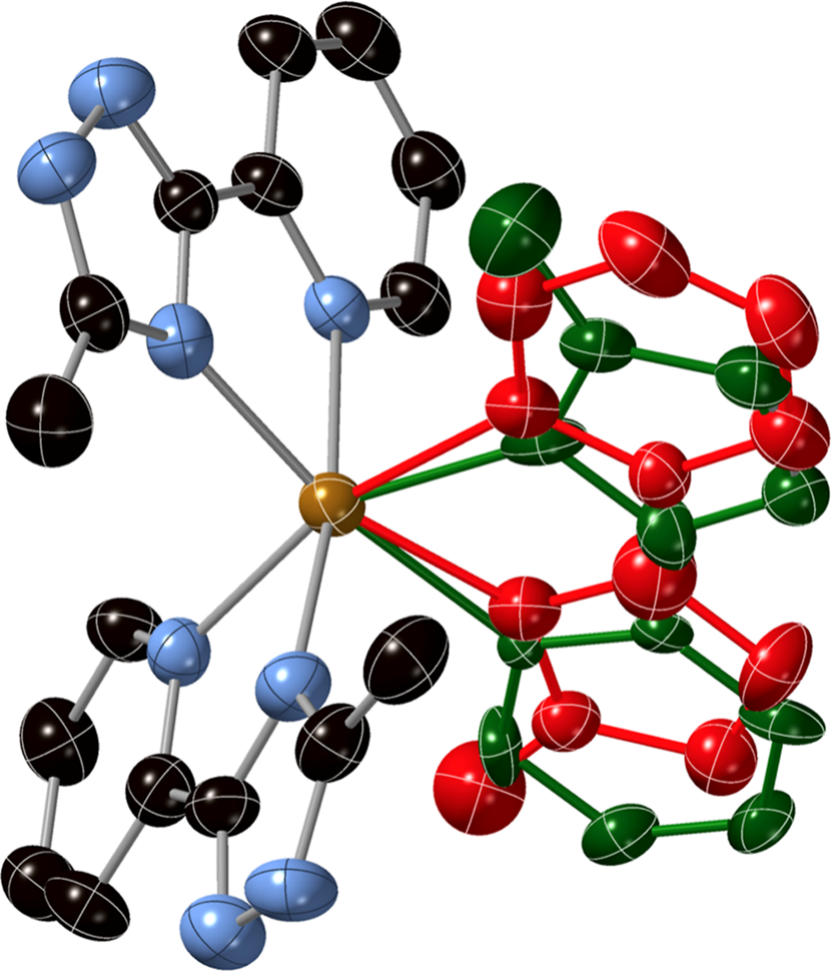
Structure of the [Fe­(**L3**)_3_]^2+^ complex with Fe1 in the structure of **3­[ClO**_**4**_ **]**_**2**_ **·0.5EtOH** at 120 K showing the two possible configurations (*fac* in green and *mer* in red). Fe (orange), C (black), and N (blue).

## Conclusions

The preparation of Fe­(II) complexes of 2-pyridyl-1,2,4-triazole ligands has given rise to a new family of SCO compounds. Among them, **1­[ClO**_**4**_ **]**_**2**_ and **1­[BF**_**4**_ **]**_**2**_ present the most interesting SCO properties such as abrupt and complete thermal spin transitions associated with an unusual combination of structural phase transitions. Further characterization is needed to prove that analogous phase transitions could be provoked by light irradiation at different wavelengths, as suggested by the steps in the LIESST curve and the reverse LIESST effects shown by **1­[ClO**_**4**_ **]**_**2**_, or by fast cooling in view of the stepwise TIESST curve of **1­[BF**_**4**_ **]**_**2**_. These properties contrast with the incomplete and gradual SCO found in the other compounds of this work and the few related compounds reported in the literature. Several structural factors could explain these differences. The most important is the strong intermolecular interactions due to hydrogen bonds between NH and N groups, which are not observed in the other related compounds. The second factor is the absence of solvent molecules, which is not the most decisive structural factor, as only one compound displays a complete SCO associated with the loss of solvent molecules (**2­[BF**_**4**_ **]**_**2**_), but it is not as abrupt and complete as that of **1­[ClO**_**4**_ **]**_**2**_ and **1­[BF**_**4**_ **]**_**2**_. Finally, the third factor is the flexibility of the ethyl substituents that could be related to the structural changes associated with the spin transition. Thus, this substituent presents different configurations in the intermediate phases that lead to the loss of an inversion center and pairs of HS/LS complexes.

In view of these results, we could envision strategies to design compounds based on these types of ligands with optimal SCO properties in a rational way. Some possibilities are the use of other substituents similar to ethyl that favors their compact packing and the absence of solvent molecules; the use of substituents favoring intermolecular interactions with the N or NH groups; and the use of solvents and/or counteranions that cannot form hydrogen bonds with the triazole ligands. This last strategy has been already tested by us with a tetraphenylborate counteranion. Preliminary results show that the presence of this counteranion favors the formation of hydrogen bonds between the complexes and a complete SCO. Furthermore, the use of chiral counteranions could lead to the stabilization of polar phases affording interesting magnetoelectric effects.^30^ Bidentate 1,2,4-triazole-based ligands, which can be a source of chiral complexes, are optimal candidates then for this type of properties.

## Supplementary Material



## Notes

Experimental details of the syntheses and characterization of all the compounds, additional details of the structural and magnetic properties of **1­[ClO**_**4**_ **]**_**2**_, **1­[BF**_**4**_ **]**_**2**_, **2­[BF**_**4**_ **]**_**2**_ **·EtOH**, **2­[BF**_**4**_ **]**_**2,**_ and **3­[ClO**_**4**_ **]**_**2**_ **·0.5EtOH**, and structural and magnetic properties of **1b­[BF**_**4**_ **]**_**2**_, **2­[ClO**_**4**_ **]**_**2**_ **·EtOH**, **3­[BF**_**4**_ **]**_**2**_ **·EtOH**, **4­[ClO**_**4**_ **]**_**2**_ **·Me**_**2**_ **CO·H**_**2**_ **O**, **5­[ClO**_**4**_ **]**_**2**_ **·EtOH**, **5­[BF**_**4**_ **]**_**2**_ **·EtOH**, **6­[ClO**_**4**_ **]**_**2**_ **·H**_**2**_ **O,** and **6­[BF**_**4**_ **]**_**2**_ **·H**_**2**_ **O** (PDF)
