# Supplementary material for: Stepwise Spin Transitions of Spin-Crossover Complexes Based in 3-(2-Pyridyl)-1,2,4-triazole Ligands Associated with Symmetry Change in Hydrogen Bonding Interactions
Source: Inorg Chem. 2025 Mar 25;64(13):6442–51. doi: 10.1021/acs.inorgchem.4c04952 (PMC12128029; doi:10.1021/acs.inorgchem.4c04952)
Supplement: Supplementary file 1 [file ic4c04952_si_001.pdf]

Stepwise spin transitions of spin-crossover complexes based in 3-(2-pyridyl)-1,2,4- triazole ligands associated with symmetry change in Hydrogen bonding interactions

Yuliia P. Petrenko,[a] José Troya,[a] Víctor García-López,[a] Dmytro M. Khomenko,[b] Roman O. Doroshchuk,[b] Rostyslav D. Lampeka,[b] Miguel Clemente-León*[a] and Eugenio Coronado[a]

[a.] *Instituto de Ciencia Molecular (ICMol), Universidad de Valencia, Catedrático José Beltrán 2, 46980 Paterna, Spain. E-mail: miguel.clemente@uv.es.*

[b.] *Department of Chemistry, Taras Shevchenko National University of Kyiv, 12, Hetman Pavlo Skoropadsky st., 01033 Kyiv, Ukraine. E-mail address: dkhomenko@ukr.net.*

Supporting Information

1. **Synthesis and characterization of the ligands**
2. **Structural characterization of the coordination compounds**
3. **Magnetic characterization of the coordination compounds**

1. Synthesis and characterization of the ligands

All the starting materials were obtained from Enamine Ltd. and UORSY. Ligands [**L1-6**] were obtained as described previously, where **L** = 3-(2-pyridyl)-5R-1,2,4-triazole, R = Et (**L1**), i-Pr (**L2**), Me (**L3**), Bz (**L4**), c-Pr (**L5**) and C_5H_9O (**L6**).[1,2] Respective hydrazides used in synthesis were prepared by standard procedures.[3]

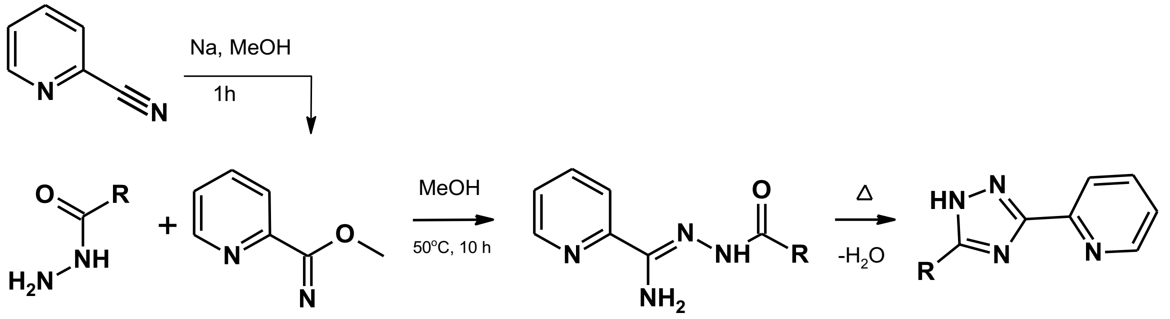

Scheme S1. Synthesis of 3-(2-pyridyl)-5R-1,2,4-triazoles (**L1-6**) where R = Et (**L1**); R = i-Pr (**L2**); R = Me (**L3**); R = Bz (**L4**); R = cPr (**L5**); R = C_5H_9O (**L6**).

Synthesis of 5-substituted 3-(2-pyridyl)-1,2,4-triazoles (*General method*). 2.5 ml of sodium methoxide methanol solution (35 wt%) was added to solution of 2-cyanopyridine (3.12 g, 0.03 mol) in 20 ml of methanol and stirred at room temperature for 1 hour. Then respective hydrazides (0.025 mol) were added to the iminoester solution and refluxed for 10 hours. The reaction mixture obtained was cooled to room temperature and solvent removed in *vacuo*. The resulting suspension was diluted with water and acidified with 1 ml of acetic acid to produce a white solid. The crystals were separated *via* filtration, dried and recrystallized from toluene.

3-(2-pyridyl)-5-benzyl-1,2,4-triazole (L4). Yield: 5.05 g, (86%), white crystals. m. p.:163-165 ˚C. IR (KBr, cm^{-1}): 3432, 3141, 3011, 2915, 2792, 2689, 2630, 1887, 1596, 1536, 1453, 1401, 1324, 1059, 1001, 801, 703. LC/MS: m/z 237 $[L+H]^+$. Elemental analysis: Anal. Calcd. For $C_{14}H_{12}N_4$ (236.27): C, 71.17%; H, 5.12%; N, 23.71%. Found: C: 71.46% H: 5.27% N: 23.28%.

3-(2-pyridyl)-5-(tetrahydro-2H-pyran-4-yl)-1,2,4-triazole (L6). Yield: 4.71 g, (82%), white crystals. m. p.:145-147 ˚C. IR (KBr, cm^{-1}): 3443, 3182, 2972, 2847, 1595, 1476, 1313, 1247, 1123, 1012, 808, 742, 565. LC/MS: m/z 231 $[L+H]^+$. Elemental analysis: Anal. Calcd. For $C_{12}H_{14}N_4O$ (230.27): C, 62.59%; H, 6.13%; N, 24.33%. Found: C: 62.42% H: 6.29% N: 24.58%.

Structural Characterization

Single crystals of all the compounds were mounted on a glass fiber using a viscous hydrocarbon oil to coat the crystal and then transferred directly to the cold nitrogen stream for data collection. X-ray data were collected at 90, 150, 180, 200, 250 and 300 K for **1[ClO$_4$]$_2$**, 100, 150, 230 and 300 K for **1[BF$_4$]$_2$**, 90, 120 and 300 K for **1b[BF$_4$]$_2$**, 100 and 300 K for **2[BF$_4$]$_2$** and 120 K for the other compounds on Rigaku Oxford diffraction Supernova diffractometer equipped with a graphite-monochromated Enhance (Mo) X-ray Source (λ = 0.71073 Å) and XtaLAB Synergy-DW diffractometer equipped with a HyPix detector and a Cu X-ray source (λ = 1.54184 Å). The program CrysAlisPro, Rigaku Oxford Diffraction Ltd., was used for unit cell determinations and data reduction. Empirical absorption correction was performed using spherical harmonics, implemented in the SCALE3 ABSPACK scaling algorithm. The structures were solved with the ShelXT structure solution program[4] and refined with the SHELXL-2013 program,[5] using Olex2.[6] Non-hydrogen atoms were refined anisotropically, and hydrogen atoms were placed in calculated positions refined using idealized geometries (riding model) and assigned fixed isotropic displacement parameters. Crystallographic data are summarized in **Tables S1-S5.** CCDC-2370949-2370974 contain the supplementary crystallographic data for this paper. For powder X-ray diffraction (PXRD) patterns, a 0.5 mm glass capillary was filled with polycrystalline samples of the complexes and mounted and aligned on an Empyrean PANalytical powder diffractometer, using Cu Kα radiation (λ = 1.54056 Å). A total of three scans were collected for each compound in the 2θ range of 2−40°.

Physical characterization

Elemental analyses (C, H, and N) were performed with a CE Instruments EA 1110 CHNS elemental analyzer. Magnetic measurements were performed with a Quantum Design MPMS-XL-5 SQUID magnetometer in the 5−400 K temperature range with an applied magnetic field of 0.1 T. Photomagnetic measurements were performed irradiating with a 30993 cylindrical Helium-Neon Laser system from Research Electro-Optics (red light, λ = 633 nm, optical power 12 $mWcm^{-2}$) or MDL-H-808-5W system from Microbeam (λ = 808 nm, optical power 8.5 $mWcm^{-2}$) coupled via an optical fiber to the cavity of the SQUID magnetometer. Significant change in magnetic response due to heating of the sample was not observed. The photomagnetic samples consisted of a thin layer of compound whose weight was corrected by comparison of a thermal spin-crossover curve with that of a more accurately weighted sample of the same compound. The samples measured in contact with the mother liquor were protected with a layer of Santovac® 5 Cryo Oil from Hampton Research.

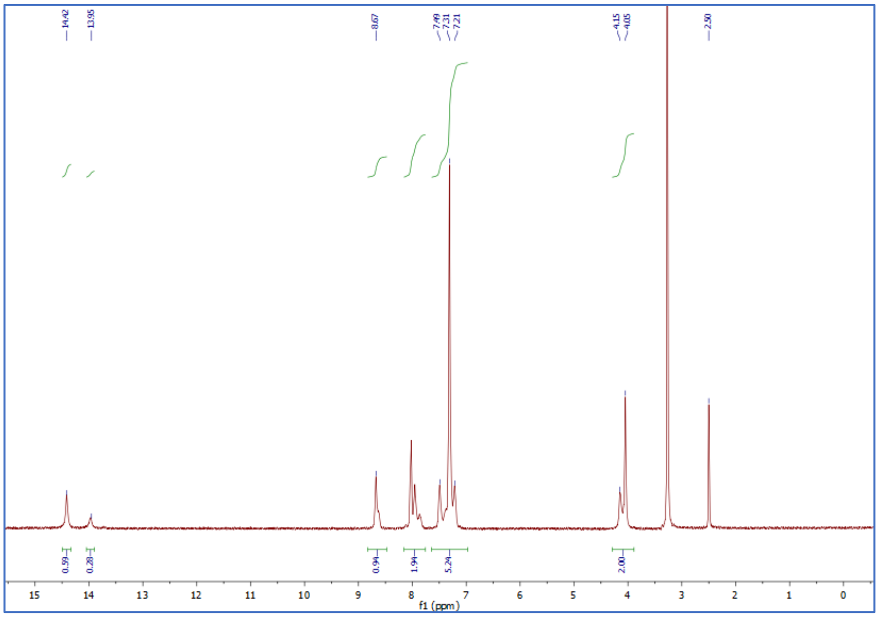

Figure S1 ^{1}H NMR spectrum of **L4** (400 MHz, DMSO-d_6) δ [ppm]: 14.42 (bs, 0.6H, $N^{1}H$) and 13.95 (bs, 0.3H, $N^{2}H$); 8.75-8.55 (m, 1H, Py-H^{6}); 8.10-7.80 (m, 2H, Py-H^{3} and Py-H^{4}); 7.55-7.10 (m, 6H, Py-H^{5} and Ph); 4.20-4.00 (m, 2H, CH_2).

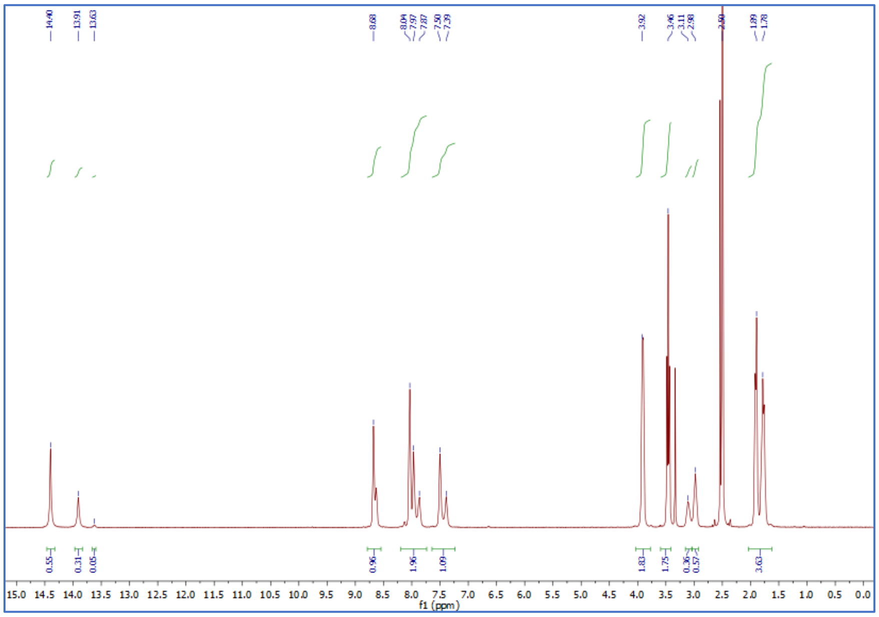

Figure S2 ^{1}H NMR spectrum of **L6** (400 MHz, DMSO-d_6) δ [ppm]: 14.40 (bs, 0.55H, $N^{1}H$), 13.91 (bs, 0.3H, $N^{2}H$) and 13.63 (bs, 0.05H, $N^{4}H$); 8.75-8.55 (m, 1H, Py-H^{6}); 8.10-7.80 (m, 2H, Py-H^{3} and Py-H^{4}); 7.55-7.30 (m, 1H, Py-H^{5}); 4.00-3.80 (m, 2H, H^{THP}), 3.50-3.30 (m, 2H, H^{THP}); 3.11 (m, 0.35H, (H^{THP})) and 2.98 (m, 0.55H, (H^{THP})); 1.95-1.70 (m, 2H, H^{THP}).

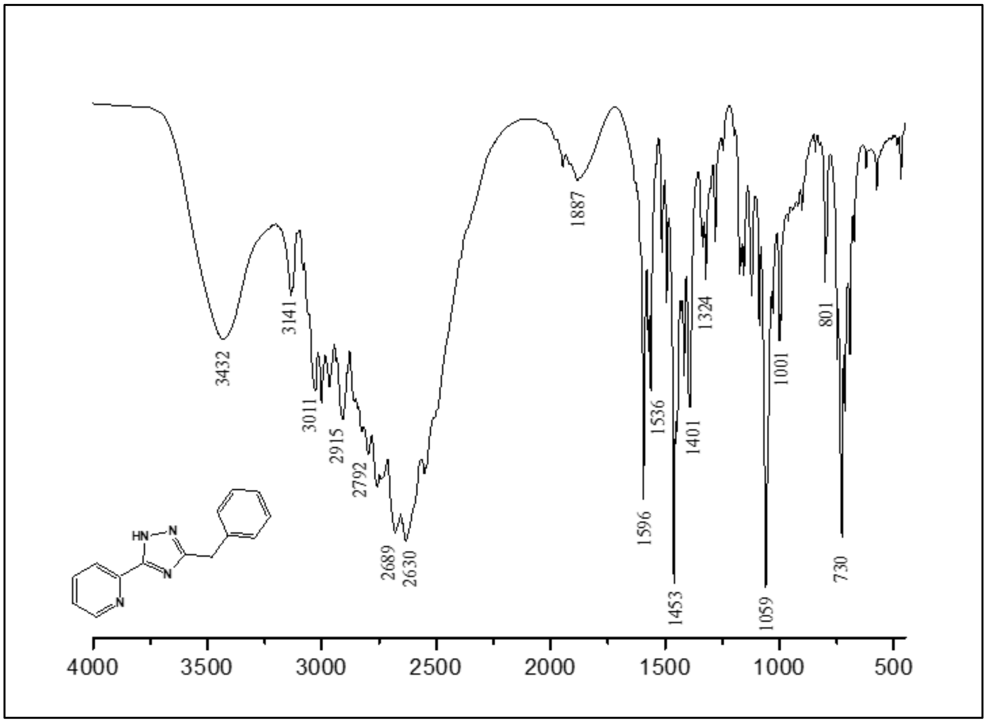

Figure S3 IR spectrum of of **L4**

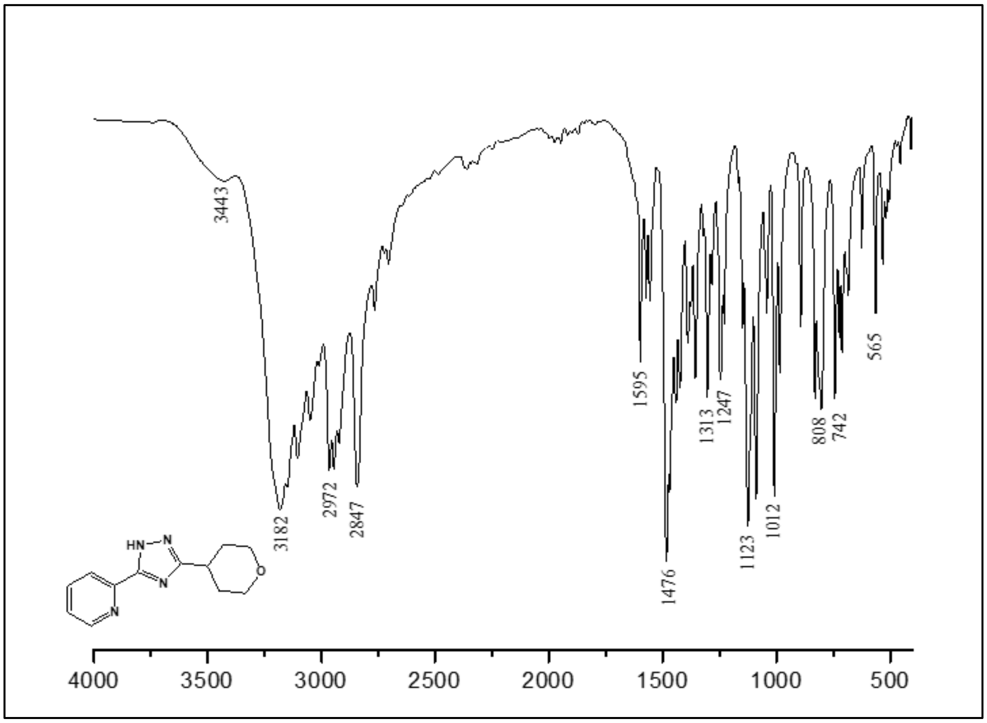

Figure S4 IR of **L6.**

2. Structural characterization of the coordination compounds

Table S1 Crystal data and structure refinement for **3[ClO$_4$]$_2$·0.5EtOH**, **3[BF$_4$]$_2$·EtOH**, **2[ClO$_4$]$_2$·EtOH**, **2[BF$_4$]$_2$·EtOH** and **2[BF$_4$]$_2$**.

Compound	**3[ClO$_4$]$_2$·0.5EtOH**	**3[BF$_4$]$_2$·EtOH**	**2[ClO$_4$]$_2$·EtOH**	**2[BF$_4$]$_2$·EtOH**	**2[BF$_4$]$_2$**	**2[BF$_4$]$_2$**
Empirical formula	$C_{50}H_{48}Cl_4Fe_2N_{24}O_{17}$	$C_{26}H_{30}B_2F_8FeN_{12}O$	$C_{32}H_{36}Cl_2FeN_{12}O_9$	$C_{32}H_{36}B_2F_8FeN_{12}O$	$C_{30}H_{36}B_2F_8FeN_{12}$	$C_{30}H_{36}B_2F_8FeN_{12}$
Formula weight	1510.62	756.09	859.34	834.20	794.18	794.18
Temperature/K	120.00(10)	120.00(10)	120.00(10)	120.00(10)	102(4)	299.99(11)
Crystal system	monoclinic	monoclinic	triclinic	triclinic	triclinic	triclinic
Space group	$P2_1/n$	$P2_1/n$	P-1	P-1	P-1	P-1
a/Å	20.6735(8)	13.0275(2)	11.0759(2)	11.0986(5)	11.0783(4)	10.9103(3)
b/Å	13.1615(4)	17.3379(3)	12.0807(3)	12.0013(6)	11.7988(4)	12.2620(5)
c/Å	24.8875(8)	14.3319(2)	16.7071(4)	16.6115(8)	16.3602(9)	16.6560(7)
α/°	90	90	86.974(2)	85.979(4)	93.337(4)	92.250(3)
β/°	113.066(4)	93.7030(10)	82.929(2)	81.571(4)	96.796(4)	94.723(3)
γ/°	90	90	64.639(2)	64.761(5)	115.961(4)	115.863(4)
Volume/Å^3	6230.3(4)	3230.38(9)	2004.68(8)	1979.70(18)	1894.61(15)	1991.25(14)
Z	4	4	2	2	2	2
ρ_{calc}g/cm^3	1.610	1.555	1.424	1.399	1.392	1.325
μ/mm^{-1}	0.727	4.557	4.808	3.773	3.891	3.703
F(000)	3088.0	1544.0	888.0	856.0	816.0	816.0
Crystal size/mm^3	0.16 × 0.14 × 0.14	0.28 × 0.17 × 0.1	0.23 × 0.19 × 0.09	0.2 × 0.14 × 0.05	0.17 × 0.13 × 0.03	0.17 × 0.13 × 0.03
Radiation	Mo Kα (λ = 0.71073)	CuKα (λ = 1.54184)	Cu Kα (λ = 1.54184)	Cu Kα (λ = 1.54184)	CuKα (λ = 1.54184)	Cu Kα (λ = 1.54184)
2Θ range for data collection/°	5.728 to 55.714	8.014 to 153.43	5.33 to 153.026	5.378 to 153.906	5.482 to 154.81	5.342 to 157.024
Index ranges	$-24 \le h \le 26, -17 \le k \le 17, -32 \le l \le 32$	$-15 \le h \le 16, -21 \le k \le 21, -17 \le l \le 18$	$-13 \le h \le 13, -15 \le k \le 15, -20 \le l \le 20$	$-13 \le h \le 14, -15 \le k \le 15, -20 \le l \le 19$	$-10 \le h \le 13, -14 \le k \le 14, -19 \le l \le 20$	$-10 \le h \le 13, -15 \le k \le 15, -19 \le l \le 20$
Reflections collected	139349	48506	63412	24845	32501	36237
Independent reflections	13954 [R_{int} = 0.1530, R_{sigma} = 0.1352]	6636 [R_{int} = 0.0557, R_{sigma} = 0.0266]	7970 [R_{int} = 0.0526, R_{sigma} = 0.0244]	8001 [R_{int} = 0.0462, R_{sigma} = 0.0461]	7620 [R_{int} = 0.0831, R_{sigma} = 0.0573]	8037 [R_{int} = 0.0634, R_{sigma} = 0.0399]
Data/restraints/parameters	13954/90/1006	6636/56/609	7970/20/572	8001/0/521	7620/0/484	8037/14/503
Goodness-of-fit on F^2	1.040	1.051	1.076	1.102	1.097	2.192
Final R indexes [I>=2σ (I)]	R_1 = 0.0901, wR_2 = 0.1796	R_1 = 0.0501, wR_2 = 0.1149	R_1 = 0.0849, wR_2 = 0.2452	R_1 = 0.0851, wR_2 = 0.2889	R_1 = 0.1818, wR_2 = 0.5089	R_1 = 0.1580, wR_2 = 0.4844
Final R indexes [all data]	R_1 = 0.1945, wR_2 = 0.2192	R_1 = 0.0535, wR_2 = 0.1168	R_1 = 0.0860, wR_2 = 0.2456	R_1 = 0.0945, wR_2 = 0.2963	R_1 = 0.1942, wR_2 = 0.5144	R_1 = 0.1761, wR_2 = 0.5018
Largest diff. peak/hole / e Å^{-3}	0.83/-0.55	0.58/-0.66	1.26/-0.75	1.77/-0.68	1.92/-0.95	1.67/-1.10

Table S2 Crystal data and structure refinement for **1[ClO₄]₂** in range 90-300K.

Compound	**1[ClO$_4$]$_2$**	**1[ClO$_4$]$_2$**	**1[ClO$_4$]$_2$**	**1[ClO$_4$]$_2$**	**1[ClO$_4$]$_2$**	**1[ClO$_4$]$_2$**
Empirical formula	$C_{27}H_{30}Cl_2FeN_{12}O_8$	$C_{81}H_{90}Cl_6Fe_3N_{36}O_{24}$	$C_{27}H_{30}Cl_2FeN_{12}O_8$	$C_{54}H_{60}Cl_4Fe_2N_{24}O_{16}$	$C_{27}H_{30}Cl_2FeN_{12}O_8$	$C_{27}H_{30}Cl_2FeN_{12}O_8$
Formula weight	777.38	2332.46	777.38	1554.76	777.38	777.38
Temperature/K	94(2)	151(2)	179.99(11)	201(2)	251(1)	300.6(10)
Crystal system	triclinic	triclinic	triclinic	triclinic	triclinic	triclinic
Space group	P-1	P-1	P-1	P-1	P-1	P-1
a/Å	10.7037(6)	12.2483(5)	12.34240(10)	12.3533(7)	10.871(4)	10.8877(9)
b/Å	12.1165(5)	14.8974(6)	14.9763(2)	14.9953(8)	12.178(5)	12.2182(12)
c/Å	14.3035(6)	26.9305(12)	19.4917(2)	19.4911(11)	14.561(4)	14.6530(8)
α/°	79.060(3)	97.482(3)	110.8990(10)	110.956(5)	83.89(3)	84.364(6)
β/°	70.688(4)	90.187(4)	97.6590(10)	97.537(4)	70.99(3)	70.811(6)
γ/°	63.863(5)	98.524(3)	98.4370(10)	98.515(4)	64.45(4)	64.765(9)
Volume/Å^3	1569.52(14)	4817.0(4)	3261.60(7)	3267.6(3)	1643.2(11)	1663.3(3)
Z	2	2	4	2	2	2
ϱ_{calc}g/cm^3	1.645	1.608	1.583	1.580	1.571	1.552
μ/mm^{-1}	0.723	0.706	5.817	0.694	0.690	0.682
F(000)	800.0	2400.0	1600.0	1600.0	800.0	800.0
Crystal size/mm^3	0.25 × 0.12 × 0.08	0.25 × 0.12 × 0.08	0.12 × 0.1 × 0.05	0.25 × 0.12 × 0.08	0.25 × 0.12 × 0.08	0.25 × 0.12 × 0.08
Radiation	Mo Kα (λ = 0.71073)	Mo Kα (λ = 0.71073)	Cu Kα (λ = 1.54184)	Mo Kα (λ = 0.71073)	MoKα (λ = 0.71073)	Mo Kα (λ = 0.71073)
2Θ range for data collection/°	6.01 to 61.132	5.586 to 59.996	4.954 to 158.562	5.726 to 59.98	5.924 to 49.414	5.914 to 59.85
Index ranges	-15 ≤ h ≤ 14, -16 ≤ k ≤ 16, -20 ≤ l ≤ 20	-15 ≤ h ≤ 16, -20 ≤ k ≤ 14, -30 ≤ l ≤ 37	-14 ≤ h ≤ 15, -19 ≤ k ≤ 18, -24 ≤ l ≤ 24	-16 ≤ h ≤ 16, -14 ≤ k ≤ 21, -26 ≤ l ≤ 27	-11 ≤ h ≤ 11, -14 ≤ k ≤ 14, -15 ≤ l ≤ 13	-15 ≤ h ≤ 14, -16 ≤ k ≤ 16, -19 ≤ l ≤ 13
Reflections collected	27295	41230	153402	27958	7564	14181
Independent reflections	8732 [R_{int} = 0.0408, R_{sigma} = 0.0520]	24205 [R_{int} = 0.0505, R_{sigma} = 0.1296]	13653 [R_{int} = 0.0797, R_{sigma} = 0.0284]	16420 [R_{int} = 0.0451, R_{sigma} = 0.1186]	4163 [R_{int} = 0.0540, R_{sigma} = 0.1282]	8352 [R_{int} = 0.0370, R_{sigma} = 0.0890]
Data/restraints/parameters	8732/0/454	24205/0/1360	13653/48/989	16420/372/1025	4163/22/487	8352/179/511
Goodness-of-fit on F^2	1.029	1.032	1.112	1.023	1.034	1.026
Final R indexes [I>=2σ (I)]	R_1 = 0.0431, wR_2 = 0.0913	R_1 = 0.0745, wR_2 = 0.1456	R_1 = 0.0584, wR_2 = 0.1589	R_1 = 0.0694, wR_2 = 0.1271	R_1 = 0.0697, wR_2 = 0.1141	R_1 = 0.0647, wR_2 = 0.1291
Final R indexes [all data]	R_1 = 0.0629, wR_2 = 0.1030	R_1 = 0.1654, wR_2 = 0.2103	R_1 = 0.0808, wR_2 = 0.1799	R_1 = 0.1647, wR_2 = 0.1867	R_1 = 0.1291, wR_2 = 0.1539	R_1 = 0.1256, wR_2 = 0.1722
Largest diff. peak/hole / e Å^{-3}	0.63/-0.70	1.34/-0.94	0.59/-1.14	0.73/-0.51	0.47/-0.44	0.40/-0.40

Table S3 Crystal data and structure refinement for **1[BF$_4$]$_2$** in range 90-300K.

Compound	**1[BF$_4$]$_2$**	**1[BF$_4$]$_2$**	**1[BF$_4$]$_2$**	**1[BF$_4$]$_2$**	**1[BF$_4$]$_2$**
Empirical formula	$C_{27}H_{30}B_2F_8FeN_{12}$	$C_{81}H_{90}B_6F_{24}Fe_3N_{36}$	$C_{54}H_{60}B_4F_{16}Fe_2N_{24}$	$C_{27}H_{30}B_2F_8FeN_{12}$	$C_{27}H_{29}B_2F_8FeN_{12}$
Formula weight	752.10	2256.29	1504.20	752.10	751.09
Temperature/K	99.9(5)	119.98(16)	149.98(10)	230.00(10)	299.99(10)
Crystal system	triclinic	triclinic	triclinic	triclinic	triclinic
Space group	P-1	P-1	P-1	P-1	P-1
a/Å	10.6973(3)	12.25870(10)	12.3713(2)	10.8326(3)	10.8520(10)
b/Å	12.0809(3)	14.8476(2)	14.9548(3)	12.1459(2)	12.1979(11)
c/Å	14.2280(4)	26.6681(3)	19.3344(3)	14.4220(4)	14.5314(13)
α/°	83.853(2)	97.1510(10)	111.561(2)	84.399(2)	84.739(7)
β/°	70.608(2)	90.3650(10)	96.8800(10)	71.437(2)	71.401(8)
γ/°	63.896(2)	99.3670(10)	99.7070(10)	65.194(2)	65.384(9)
Volume/Å^3	1555.85(8)	4750.19(9)	3214.04(11)	1631.41(8)	1655.5(3)
Z	2	2	2	2	2
ϱ_{calc}g/cm^3	1.605	1.577	1.554	1.531	1.507
μ/mm^{-1}	4.704	4.622	4.554	4.486	4.420
F(000)	768.0	2304.0	1536.0	768.0	766.0
Crystal size/mm^3	0.18 × 0.15 × 0.08	0.18 × 0.15 × 0.08	0.18 × 0.15 × 0.08	0.18 × 0.15 × 0.08	0.18 × 0.15 × 0.08
Radiation	CuKα (λ = 1.54184)	CuKα (λ = 1.54184)	CuKα (λ = 1.54184)	Cu Kα (λ = 1.54184)	Cu Kα (λ = 1.54184)
2Θ range for data collection/°	6.594 to 155.668	6.082 to 155.542	5.014 to 155.404	6.47 to 155.14	6.426 to 154.794
Index ranges	-13 ≤ h ≤ 13, -15 ≤ k ≤ 15, -17 ≤ l ≤ 17	-11 ≤ h ≤ 15, -18 ≤ k ≤ 18, -33 ≤ l ≤ 33	-15 ≤ h ≤ 11, -18 ≤ k ≤ 18, -23 ≤ l ≤ 24	-13 ≤ h ≤ 13, -15 ≤ k ≤ 14, -18 ≤ l ≤ 18	-13 ≤ h ≤ 13, -15 ≤ k ≤ 15, -18 ≤ l ≤ 18
Reflections collected	29554	90183	61162	31079	31621
Independent reflections	6304 [R_{int} = 0.0436, R_{sigma} = 0.0310]	19410 [R_{int} = 0.0408, R_{sigma} = 0.0321]	13042 [R_{int} = 0.0386, R_{sigma} = 0.0311]	6631 [R_{int} = 0.0459, R_{sigma} = 0.0332]	6736 [R_{int} = 0.0402, R_{sigma} = 0.0301]
Data/restraints/parameters	6304/0/454	19410/290/1487	13042/102/935	6631/210/583	6736/211/610
Goodness-of-fit on F^2	1.118	1.070	1.114	1.073	1.062
Final R indexes [I>=2σ (I)]	R_1 = 0.0698, wR_2 = 0.2128	R_1 = 0.0724, wR_2 = 0.2465	R_1 = 0.0751, wR_2 = 0.2449	R_1 = 0.0676, wR_2 = 0.1916	R_1 = 0.0700, wR_2 = 0.2255
Final R indexes [all data]	R_1 = 0.0770, wR_2 = 0.2228	R_1 = 0.0853, wR_2 = 0.2594	R_1 = 0.0871, wR_2 = 0.2600	R_1 = 0.0752, wR_2 = 0.1981	R_1 = 0.0811, wR_2 = 0.2401
Largest diff. peak/hole / e Å^{-3}	0.93/-0.68	1.25/-0.78	1.06/-0.64	0.76/-0.37	0.68/-0.40

Table S4 Crystal data and structure refinement for **1b[BF$_4$]$_2$** in range 90-300K.

Compound	**1b[BF$_4$]$_2$**	**1b[BF$_4$]$_2$**	**1b[BF$_4$]$_2$**
Empirical formula	$C_{27}H_{30}B_2F_8FeN_{12}$	$C_{27}H_{30}B_2F_8FeN_{12}$	$C_{27}H_{30}B_2F_8FeN_{12}$
Formula weight	752.10	752.10	752.10
Temperature/K	96(1)	120.0(3)	301.2(3)
Crystal system	monoclinic	monoclinic	monoclinic
Space group	$P2_1/c$	$P2_1/c$	$P2_1/c$
a/Å	10.1174(6)	10.1202(7)	10.3256(3)
b/Å	18.5458(11)	18.5166(15)	18.5919(4)
c/Å	17.6395(8)	17.6763(10)	17.8520(4)
α/°	90	90	90
β/°	99.865(5)	100.035(6)	101.533(2)
γ/°	90	90	90
Volume/Å^3	3260.9(3)	3261.7(4)	3357.90(14)
Z	4	4	4
ϱ_{calc}g/cm^3	1.532	1.532	1.488
μ/mm^{-1}	4.488	4.487	4.359
F(000)	1536.0	1536.0	1536.0
Crystal size/mm^3	0.098 × 0.064 × 0.052	0.098 × 0.064 × 0.052	0.098 × 0.064 × 0.052
Radiation	Cu Kα (λ = 1.54184)	CuKα (λ = 1.54184)	CuKα (λ = 1.54184)
2Θ range for data collection/°	6.97 to 127.612	6.97 to 157.752	6.938 to 153.86
Index ranges	$-11 \le h \le 11, -21 \le k \le 20, -20 \le l \le 19$	$-12 \le h \le 12, -23 \le k \le 23, -22 \le l \le 22$	$-12 \le h \le 12, -23 \le k \le 22, -22 \le l \le 19$
Reflections collected	20838	32018	42243
Independent reflections	5183 [R_{int} = 0.0936, R_{sigma} = 0.0851]	6854 [R_{int} = 0.1568, R_{sigma} = 0.1008]	6892 [R_{int} = 0.0690, R_{sigma} = 0.0350]
Data/restraints/parameters	5183/0/467	6854/15/482	6892/30/527
Goodness-of-fit on F^2	0.974	1.016	1.085
Final R indexes [I>=2σ (I)]	R_1 = 0.0559, wR_2 = 0.1259	R_1 = 0.0654, wR_2 = 0.1407	R_1 = 0.0582, wR_2 = 0.1487
Final R indexes [all data]	R_1 = 0.1001, wR_2 = 0.1420	R_1 = 0.1273, wR_2 = 0.1689	R_1 = 0.0845, wR_2 = 0.1694
Largest diff. peak/hole / e Å^{-3}	0.41/-0.27	0.40/-0.66	0.79/-0.42

Table S5 Crystal data and structure refinement for **4[ClO$_4$]$_2$·Me$_2$CO·H$_2$O**, **5[ClO$_4$]$_2$·EtOH**, **5[BF$_4$]$_2$·EtOH**, **6[ClO$_4$]$_2$·H$_2$O**, **6[BF$_4$]$_2$·H$_2$O**

Compound	**4[ClO$_4$]$_2$·Me$_2$CO·H$_2$O**	**5[ClO$_4$]$_2$·EtOH**	**5[BF$_4$]$_2$·EtOH**	**6[ClO$_4$]$_2$·H$_2$O**	**6[BF$_4$]$_2$·H$_2$O**
Empirical formula	$C_{45}H_{44}Cl_2FeN_{12}O_{10}$	$C_{32}H_{30}Cl_2FeN_{12}O_9$	$C_{32}H_{30}B_2F_8FeN_{12}O$	$C_{72}H_{84}Cl_4Fe_2N_{24}O_{22}$	$C_{77}H_{84}B_4F_{16}Fe_2N_{24}O_8$
Formula weight	1039.67	853.37	828.09	1891.13	1932.62
Temperature/K	120.01(10)	120.00(10)	121(1)	119.99(10)	119.98(10)
Crystal system	triclinic	triclinic	triclinic	triclinic	triclinic
Space group	P-1	P-1	P-1	P-1	P-1
a/Å	11.4878(4)	11.19280(10)	11.1745(3)	11.98920(10)	11.7640(2)
b/Å	14.0267(4)	11.74810(10)	11.6467(4)	18.7668(2)	19.1028(4)
c/Å	16.3588(6)	16.70330(10)	16.4945(3)	20.6878(2)	20.7730(5)
α/°	68.903(3)	96.5640(10)	95.656(2)	70.4180(10)	72.013(2)
β/°	85.120(3)	94.2180(10)	94.951(2)	83.8070(10)	84.270(2)
γ/°	72.670(3)	117.2240(10)	116.744(3)	89.0130(10)	89.7570(10)
Volume/Å^3	2346.94(15)	1920.49(3)	1886.99(10)	4359.03(8)	4416.14(17)
Z	2	2	2	2	2
ρ_{calc}g/cm^3	1.471	1.476	1.457	1.441	1.453
μ/mm^{-1}	4.233	5.018	3.958	4.511	3.519
F(000)	1076.0	876.0	844.0	1960.0	1988.0
Crystal size/mm^3	0.33 × 0.26 × 0.1	0.18 × 0.1 × 0.03	0.1 × 0.04 × 0.03	0.34 × 0.19 × 0.09	0.19 × 0.17 × 0.06
Radiation	Cu Kα (λ = 1.54184)	Cu Kα (λ = 1.54184)	CuKα (λ = 1.54184)	Cu Kα (λ = 1.54184)	Cu Kα (λ = 1.54184)
2Θ range for data collection/°	5.792 to 153.71	8.584 to 154.174	5.444 to 127.256	7.418 to 153.932	7.556 to 157.612
Index ranges	-13 ≤ h ≤ 14, -17 ≤ k ≤ 17, -18 ≤ l ≤ 20	-14 ≤ h ≤ 13, -14 ≤ k ≤ 14, -21 ≤ l ≤ 20	-12 ≤ h ≤ 12, -13 ≤ k ≤ 13, -19 ≤ l ≤ 18	-15 ≤ h ≤ 15, -23 ≤ k ≤ 20, -25 ≤ l ≤ 25	-13 ≤ h ≤ 14, -24 ≤ k ≤ 24, -25 ≤ l ≤ 25
Reflections collected	31362	56374	28485	160505	89817
Independent reflections	9530 [R_{int} = 0.0318, R_{sigma} = 0.0308]	7705 [R_{int} = 0.0318, R_{sigma} = 0.0183]	6080 [R_{int} = 0.0546, R_{sigma} = 0.0485]	17460 [R_{int} = 0.0747, R_{sigma} = 0.0300]	18517 [R_{int} = 0.0928, R_{sigma} = 0.0586]
Data/restraints/parameters	9530/0/636	7705/171/594	6080/93/557	17460/36/1235	18517/15/1145
Goodness-of-fit on F^2	1.051	1.050	1.045	1.027	1.929
Final R indexes [I>=2σ (I)]	R_1 = 0.0348, wR_2 = 0.0904	R_1 = 0.0496, wR_2 = 0.1534	R_1 = 0.0561, wR_2 = 0.1528	R_1 = 0.0753, wR_2 = 0.2043	R_1 = 0.1642, wR_2 = 0.4452
Final R indexes [all data]	R_1 = 0.0366, wR_2 = 0.0916	R_1 = 0.0509, wR_2 = 0.1547	R_1 = 0.0779, wR_2 = 0.1650	R_1 = 0.0860, wR_2 = 0.2123	R_1 = 0.1790, wR_2 = 0.4564
Largest diff. peak/hole / e Å^{-3}	0.38/-0.48	1.33/-0.56	0.78/-0.50	0.99/-0.90	3.65/-0.97

Packing of the $[Fe(L1)_3]^{2+}$ complexes in the structure of ($1[ClO_4]_2$).

The packing of the $[Fe(L1)_3]^{2+}$ complexes at 90 K for (**$1[ClO_4]_2$**) is formed by layer of complexes in the *bc* plane separated by a bilayer of ClO_4^- anions. In these layers, dimers of $[Fe(L1)_3]^{2+}$ complexes interact through two hydrogen bonds between NH and N from triazole. These interactions involve two of the three L1 ligands from each complex. The two NH groups not involved in these interactions form a hydrogen bond with a perchlorate counteranion. In addition, the $[Fe(L1)_3]^{2+}$ complexes of these dimers are linked through two CH···π interactions between pyrazole rings and CH_3 groups. Two neighboring complexes from different dimers present short contacts between CH groups from pyridine and N from triazole and CH···π interactions between between pyridine rings involving two of the three L1 ligands of each complex. This leads to chains of dimers running along the [0-11] direction (**Figure S5**). The complexes of these chains are connected to two complexes of different chains through two CH···π interactions of the pyridine rings with one of them and one short contact between CH groups from pyridine to the other one. This gives rise to a layer of complexes in the *bc* plane (**Figure S5**). The complexes of different layers form hydrogen bonds with ClO_4^- anions with the NH groups.

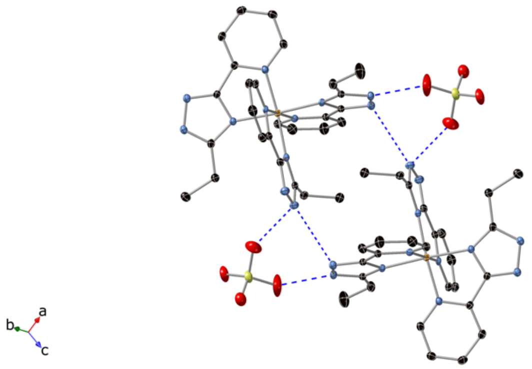

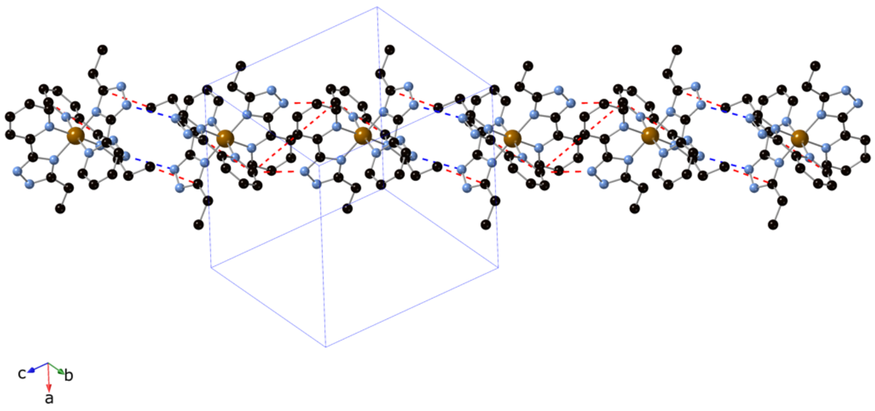

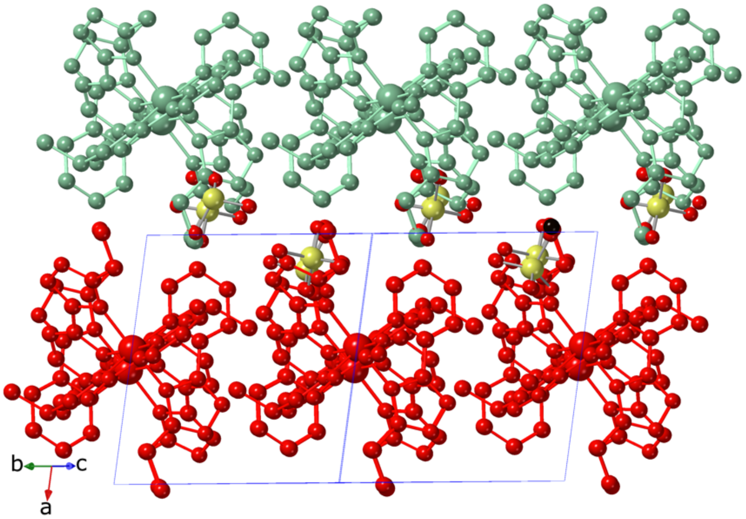

Figure S5 Structure of the hydrogen-bonded dimers including counteranions (top), chains (middle) and layers colored in red and green (bottom) of $[Fe(L1)_3]^{2+}$ complexes in the structure of (**$1[ClO_4]_2$**) at 90 K. Hydrogen bonds (blue-dashed lines), CH···π interactions and other short contacts(red-dashed lines), Fe (orange), C (black), N (blue), O(red) and Cl(yellow).

At 150 K, there is a drastic change in the unit cell (**Figure 10**). The three crystallographically independent $[Fe(L1)_3]^{2+}$ complexes called Fe1 (LS), Fe2 (HS) and Fe3 (HS) form chains linked in a similar way as that observed at 90 K but with some changes. The chains run now along the [*2-21*] direction and follow the order Fe1-Fe2-Fe3-Fe3-Fe2-Fe1 formed by three dimers linked through hydrogen bonds as those observed at 90 K. These dimers are connected through similar CH···π and N···CH contacts between pyrazole and pyridine ring as those observed at 90 K. Therefore, there are two types of dimers formed by Fe1-Fe2 and Fe3-Fe3 complexes. The intradimer interactions in Fe1-Fe2 dimers are weakened with respect to Fe3-Fe3 dimers and dimers observed at 90 K. Thus, NH···N distances of the two hydrogen bonds are increased (2.165 and 2.488 Å for Fe1-Fe2 and 2.103 Å for Fe3-Fe3). Furthermore, the two CH···π interactions between pyrazole rings and CH_3 groups are not observed in Fe1-Fe2 dimers. On the other hand, interchain interactions of Fe3 are weakened with respect to those found at 90 K with only two CH···π contacts between pyridine rings with two $[Fe(L1)_3]^{2+}$ complexes from different chains. On the contrary, Fe2 present CH···π contacts with four $[Fe(L1)_3]^{2+}$ complexes from different chains through two of the three pyridine rings. Finally, Fe1 complexes display similar interchain interactions as those observed at 90 K. These changes in intradimer and interdimer interactions could be responsible of the change in elastic interactions leading to [LS-HS-HS-HS-HS-LS] ordering and long-range spin-state concentration wave.

At 200 K, the unit cell is approximately doubled with respect to that at 90 K. There are two crystallographically independent $[Fe(L1)_3]^{2+}$ complexes in the asymmetric unit (Fe1 and Fe2 complexes) and four ClO_4^- anions (one of them with a disorder solved with two possible configuration with occupancies of 50 %). These two $[Fe(L1)_3]^{2+}$ complexes display typical HS Fe-N distances in agreement with magnetic properties (see below **Table S6**). Fe1 and Fe2 complexes form dimers linked through two NH···N hydrogen bonds and two CH···π

interactions between pyrazole rings and CH_3 groups as observed at lower temperatures. The NH···N distances are slightly different (2.265 Å for Fe1-Fe1 dimers and 2.161 Å for Fe2-Fe2 dimers). These dimers are connected through similar CH···π and N···CH contacts between pyrazole and pyridine ring as those observed at 90 K. These leads to chains formed by alternated Fe1-Fe1 and Fe2-Fe2 dimers running along the [*1-2-1*] direction. These chains are linked to neighboring ones through similar intermolecular interactions as those observed at lower temperatures plus a contact involving CH_3 and CH from pyridine.

At 250 K, there is an increase in symmetry as observed at 90 and 120 K with one crystallographically independent $[Fe(L1)_3]^{2+}$ complex and two ClO_4^- anions in the asymmetric unit. $[Fe(L1)_3]^{2+}$ complex display typical HS Fe-N bond lengths and a similar packing to that observed at 90 K. Two of the three ethyl chains and one of the two ClO_4^- anions present a disorder, which was solved with two configurations with occupancies of 50 %.

Table S6 Fe-N distances (Å), *Σ* and *Θ* distortion octahedral parameters in the $[Fe(L1)_3]^{2+}$ complexes of **($1[ClO_4]_2$)** and **$1[BF_4]_2$** at different temperatures.

T (K) **$1[ClO_4]_2$**	94(2)	120.00(10)	151(2)	179.99(11)	201(2)	251(1)	300.6(10)
Fe1-N (Å)	1.9927(17)		2.004(4)	2.179(3)	2.183(4)	2.182(6)	2.191(3)
Fe2-N (Å)			2.179(4)	2.185(3)	2.190(4)		
Fe3-N (Å)			2.180(4)				
Σ (°) Fe1	51.4(3)		56.2(6)	82.0(3)	81.8(4)	82.8(8)	83.8(4)
Θ (°) Fe1	110.3(4)		113.7(9)	199.3(6)	198.6(8)	202.4(14)	201.6(7)
Σ (°) Fe2			81.2(5)	82.8(3)	84.2(5)		
Θ (°) Fe2			202.5(9)	199.6(6)	202.2(8)		
Σ (°) Fe3			84.4(5)				
Θ (°) Fe3			198.9(9)				
T (K) **$1[BF_4]_2$**	99.9(5)	119.98(16)	149.98(10)	230.00(10)	299.99(10)		
Fe1-N (Å)	1.994(4)	2.010(4)	2.181(4)	2.186(4)	2.188(4)		
Fe2-N (Å)		2.167(4)	2.186(4)				
Fe3-N (Å)		2.174(4)					
Σ (°) Fe1	50.2(6)	53.2(6)	82.3(5)	82.3(5)	82.2(5)		
Θ (°) Fe1	113.1(11)	117.4(9)	204.4(9)	202.4(9)	200.9(10)		
Σ (°) Fe2		79.0(5)	83.2(6)				
Θ (°) Fe2		199.7(9)	202.8(10)				
Σ (°) Fe3		81.8(5)					
Θ (°) Fe3		197.3(9)					

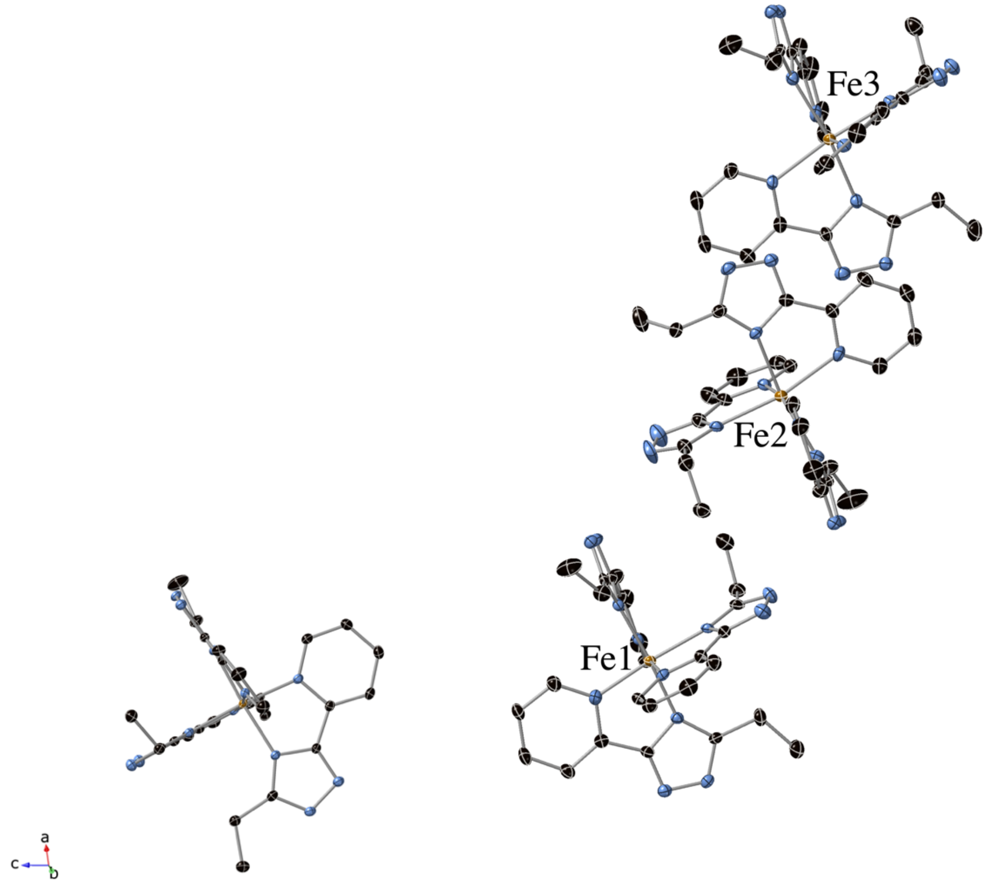

90 K

150 K

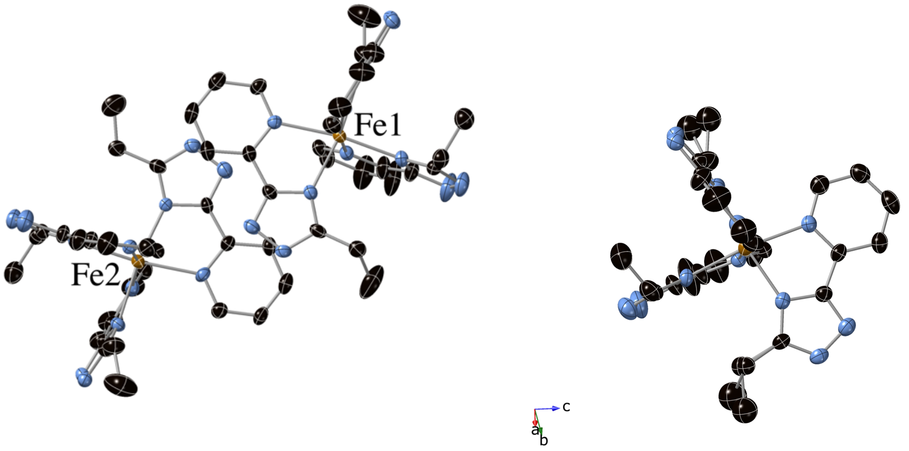

200 K

250 K

Figure S6 Structure of the $[Fe(L1)_3]^{2+}$ complexes in the structure of **$1[ClO_4]_2$** at 90, 150, 200 and 250 K. Fe (orange), C (black), N (blue), O(red) and Cl(yellow).

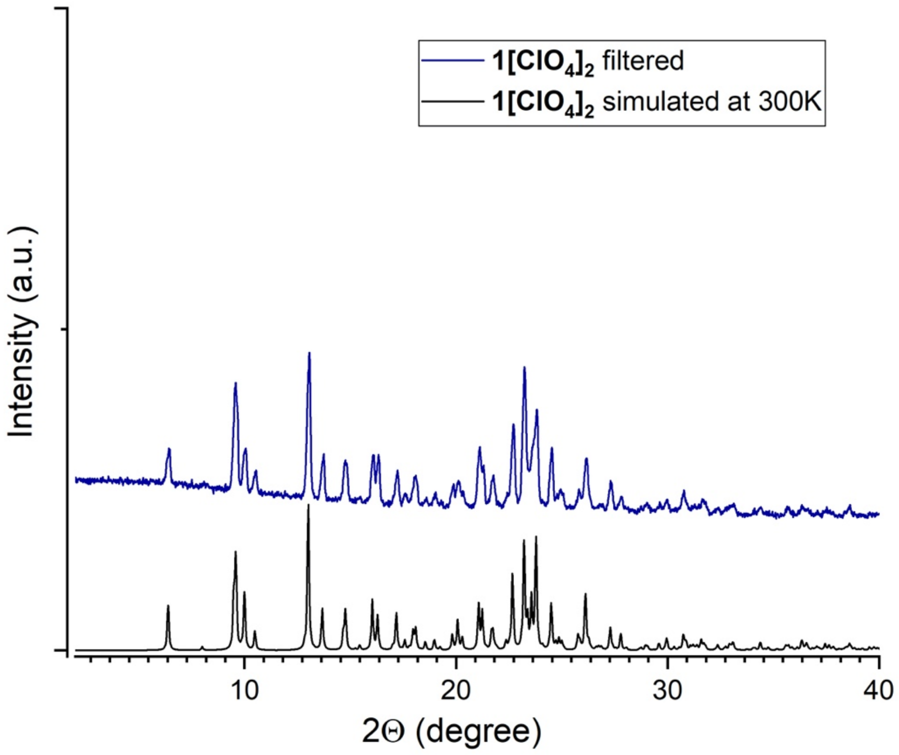

Figure S7 PXRD pattern of a filtered sample of **1[ClO$_4$]$_2$** (blue line) and simulated one from the structure solved by single crystal X-ray diffraction at 300 K (black line).

Packing of the [Fe(L1)$_3$]$^{2+}$ complexes in the structure of 1[BF$_4$]$_2$.

The packing of the [Fe(L1)$_3$]$^{2+}$ complexes at 100 K for **1[BF$_4$]$_2$** is formed by layer of complexes in the *[00-1]* plane separated by a bilayer of BF_4^- anions. As in **1[ClO$_4$]$_2$**, these layers are formed by dimers of [Fe(L1)$_3$]$^{2+}$ complexes interacting through two hydrogen bonds between NH and N from triazole. These interactions involve two of the three L1 ligands from each complex. The two NH groups not involved in these interactions form a hydrogen bond with a BF_4^- counteranion. In addition, the [Fe(L1)$_3$]$^{2+}$ complexes of these dimers are linked through two CH···π interactions between pyrazole rings and CH_3 groups. Two neighboring complexes from different dimers present short contacts between CH groups from pyridine and N from triazole and CH···π interactions between between pyridine rings involving two of the three L1 ligands of each complex. This leads to chains of dimers running along the [-111] direction (**Figure S8**). The complexes of these chains are connected to two complexes of different chains through two CH···π interactions of the pyridine rings with one of them and one short contact between CH groups from pyridine to the other one. This gives rise to the layers of (**Figure S8**). The complexes of different layers form hydrogen bonds with BF_4^- anions with the NH groups.

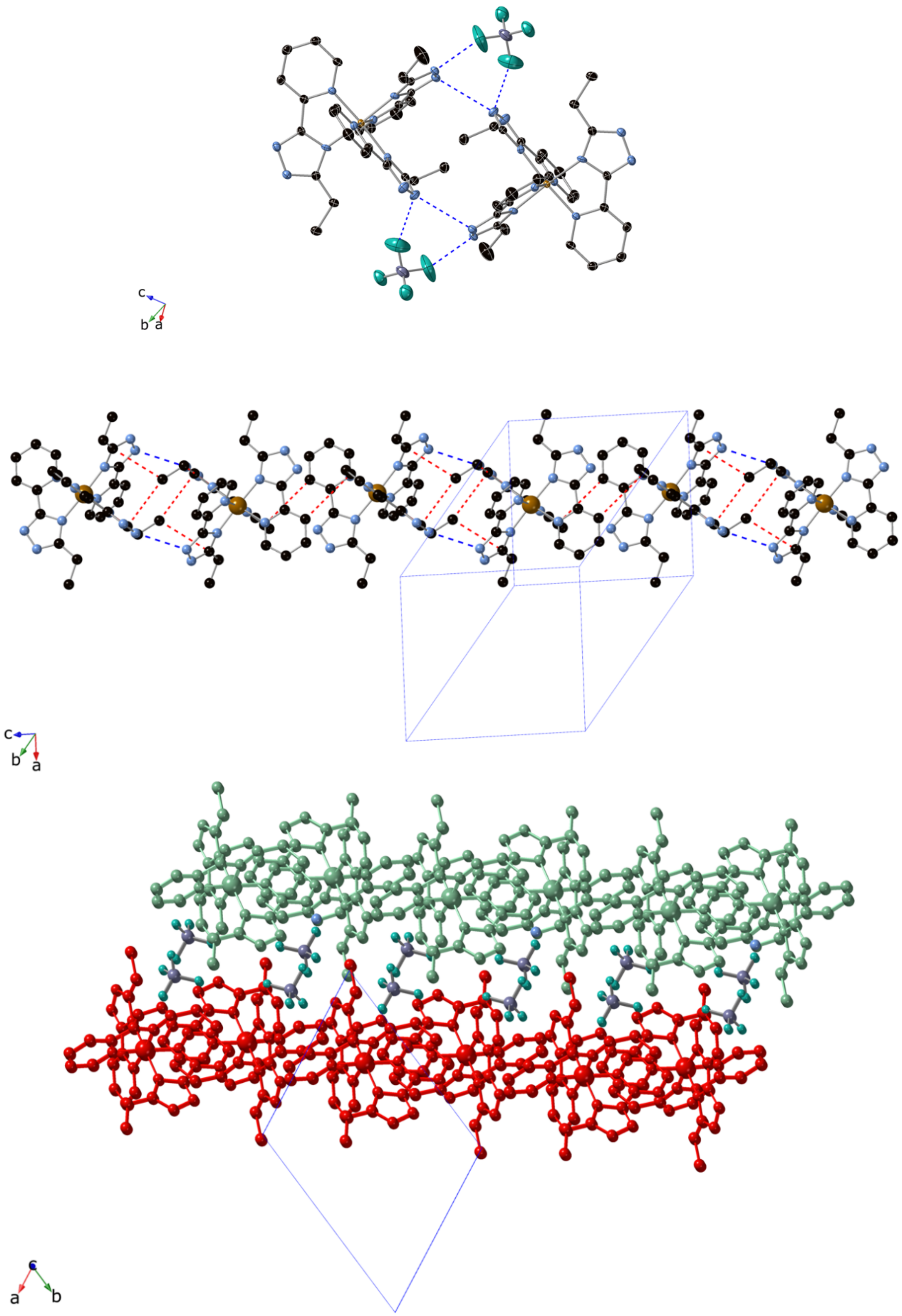

Figure S8. Structure of the hydrogen-bonded dimers including counteranions (top), chains (middle) and layers colored in red and green (bottom) of $[Fe(L1)_3]^{2+}$ complexes in the structure of **$1[BF_4]_2$** at 100 K. Hydrogen bonds (blue-dashed lines), CH···π interactions and other short contacts(red-dashed lines), Fe (orange), C (black), N (blue), O(red) and Cl(yellow).

At 120 K, there is a drastic change in the unit cell. The three crystallographically independent $[Fe(L1)_3]^{2+}$ complexes called Fe1 (LS), Fe2 (HS) and Fe3 (HS) form chains linked in a similar way as that observed at 100 K but with some changes. The chains follow the order Fe1-Fe2-Fe3-Fe3-Fe2-Fe1 formed by three dimers linked through hydrogen bonds as those observed at 150 K in **1[ClO$_4$]$_2$**. These dimers are connected through similar CH···π and N···CH contacts between pyrazole and pyridine ring as those observed at 100 K. Therefore, there are two types of dimers formed by Fe1-Fe2 and Fe3-Fe3 complexes. The intradimer interactions in Fe1-Fe2 dimers are weakened with respect to Fe3-Fe3 dimers and dimers observed at 100 K. Thus, NH···N distances of the two hydrogen bonds are increased (2.188 and 2.509 Å for Fe1-Fe2 and 2.105 Å for Fe3-Fe3). Furthermore, the two CH···π interactions between pyrazole rings and CH_3 groups are not observed in Fe1-Fe2 dimers. On the other hand, interchain interactions of Fe3 are weakened with respect to those found at 100 K with only two CH···π contacts between pyridine rings with two $[Fe(L1)_3]^{2+}$ complexes from different chains. On the contrary, Fe2 present CH···π contacts with four $[Fe(L1)_3]^{2+}$ complexes from different chains through two of the three pyridine rings. Finally, Fe1 complexes display similar interchain interactions as those observed at 100 K. These changes in intradimer and interdimer interactions could be responsible of the change in elastic interactions leading to [LS-HS-HS-HS-HS-LS] ordering and long-range spin-state concentration wave.

At 150 K, the unit cell is doubled with respect to that at 100 K. There are two crystallographically independent $[Fe(L1)_3]^{2+}$ complexes in the asymmetric unit (Fe1 and Fe2 complexes) and four BF_4^- anions (one of them with a disorder solved with two possible configurations with occupancies of 50 %). These two $[Fe(L1)_3]^{2+}$ complexes display typical HS Fe-N distances in agreement with magnetic properties (see below). Fe1 and Fe2 complexes form dimers linked through two NH···N hydrogen bonds and two CH···π interactions between pyrazole rings and CH_3 groups as observed at lower temperatures. The NH···N distances are slightly different (2.345 Å for Fe1-Fe1 dimers and 2.134 Å for Fe2-Fe2 dimers). These dimers are connected through similar CH···π and N···CH contacts between pyrazole and pyridine ring as those observed at 100 K. These leads to chains formed by alternated Fe1-Fe1 and Fe2-Fe2 dimers. These chains are linked to neighboring ones through similar intermolecular interactions as those observed at lower temperatures plus a contact involving CH_3 and CH from pyridine.

At 230 and 300 K, there is an increase in symmetry as observed at 100 K with one crystallographically independent $[Fe(L1)_3]^{2+}$ complex and two BF_4^- anions in the asymmetric unit. $[Fe(L1)_3]^{2+}$ complex displays typical HS Fe-N bond lengths and a similar packing to that observed at 100 K.

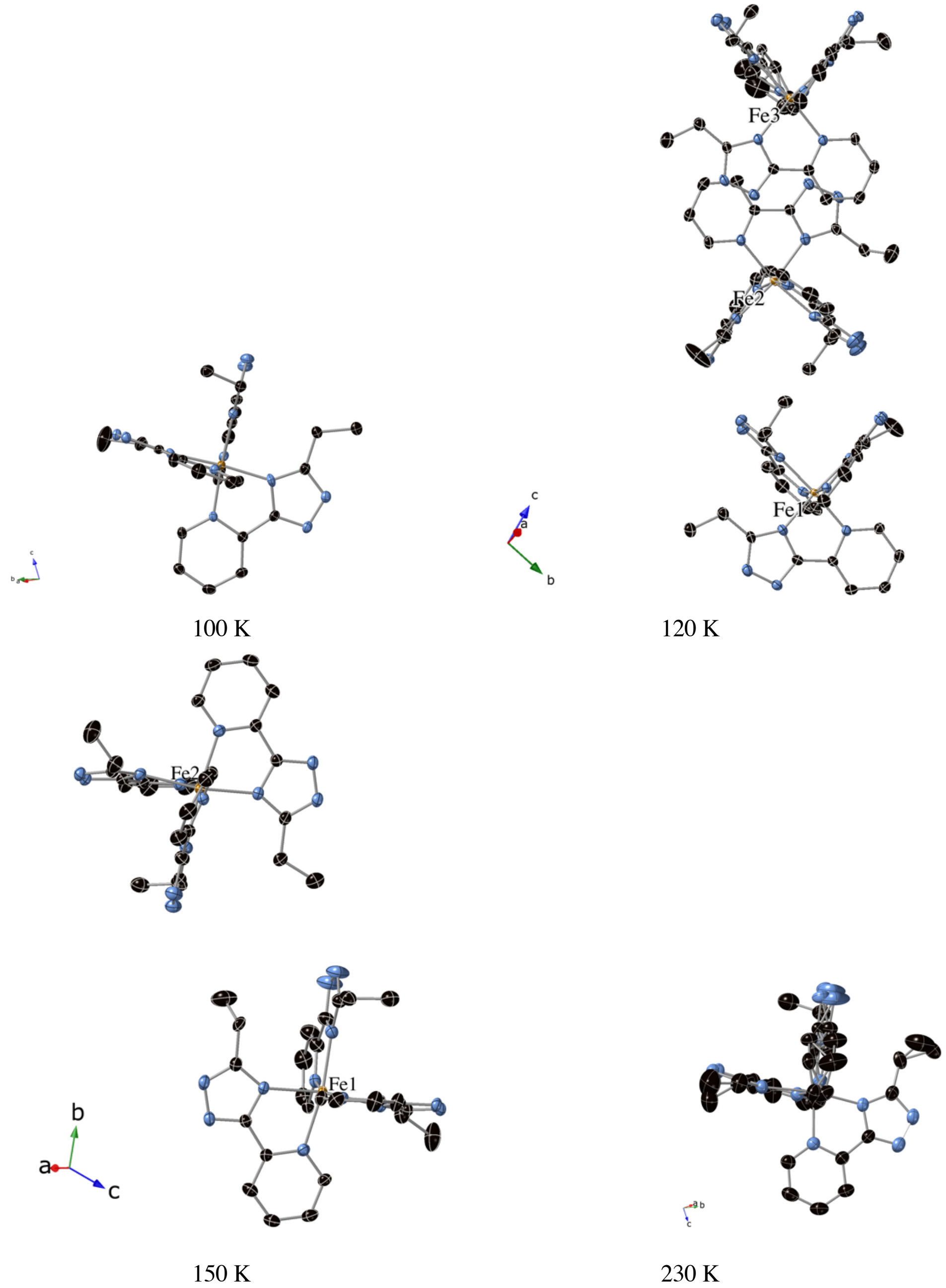

Figure S9 Structure of the $[Fe(L1)_3]^{2+}$ complexes in the structure of **1[BF$_4$]$_2$** at 100 K (1LS phase), 120 K (2HS-1LS phase), 150 K (2HS phase) and 230 K (1HS phase). Fe (orange), C (black), N (blue), O(red) and Cl(yellow).

Structure of $[Fe(L1)_3](BF_4)_2$ (1b$[BF_4]_2$). Compound **1b$[BF_4]_2$** crystallizes in monoclinic $P2_1/c$ space group. It contains one crystallographically independent $[Fe(L1)_3]^{2+}$ complex in the asymmetric unit with typical HS Fe-N bond lengths (average = 2.182(4) Å at 96 K. 2.191(4) Å at 120 K and 2.203(3) Å at 300 K) and two crystallographically independent BF_4^- anions, which present a disorder at 300 K solved with two configurations. At 96 and 120 K, only one of the two crystallographically independent BF_4^- anions present this type of disorder. Fe-N distances agree with magnetic properties. $[Fe(L1)_3]^{2+}$ complexes form hydrogen bonds with BF_4^- anions through the three NH groups (see **Figure S11**). In this structure the terminal CH_3 of three ethyl groups are clearly out of the plane defined by the triazole ring. Neighboring $[Fe(L1)_3]^{2+}$ complexes present short contacts between CH_3 groups from ethyl, between CH groups of pyridine and N from triazole, CH···π contacts between pyridine rings and ethyl groups and short contacts between CH_3 groups from ethyl and CH groups from pyridine. They do not present N···HN hydrogen bonds as those observed in the structure of **1$[BF_4]_2$** or **1$[ClO_4]_2$**, which could be related to the lack of an abrupt spin transition.

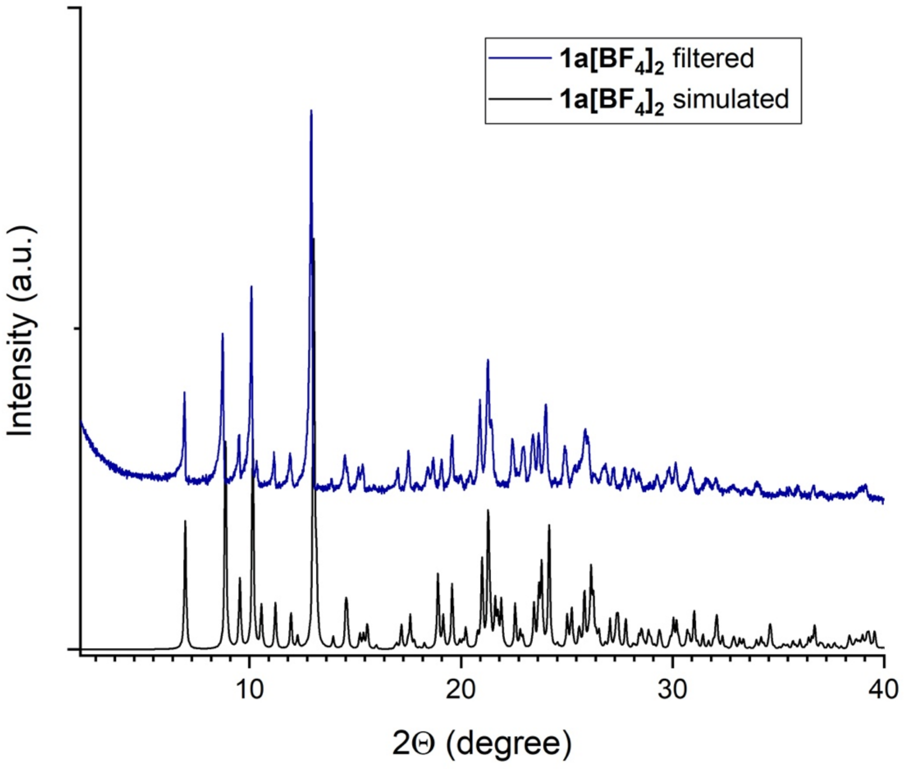

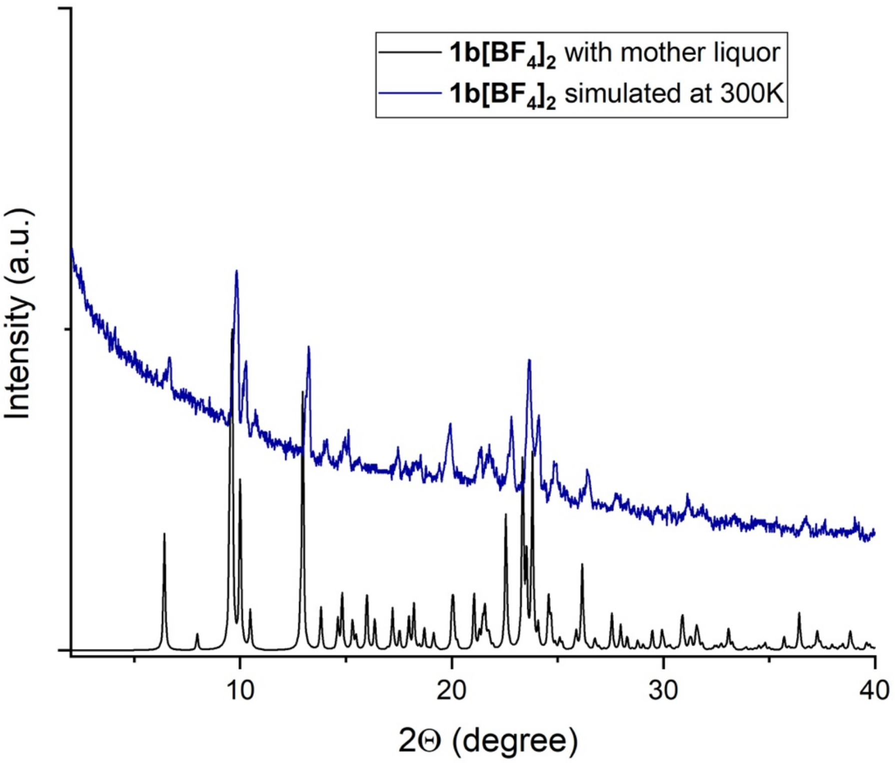

Figure S10 PXRD pattern of a filtered sample of **1a[BF$_4$]$_2$** (blue line) and simulated one from the structure solved by single crystal X-ray diffraction at 300 K (black line) (top). PXRD pattern of **1b[BF$_4$]$_2$** (blue line) in contact with mother liquor and simulated one from the structure solved by single crystal X-ray diffraction at 300 K (black line) (bottom).

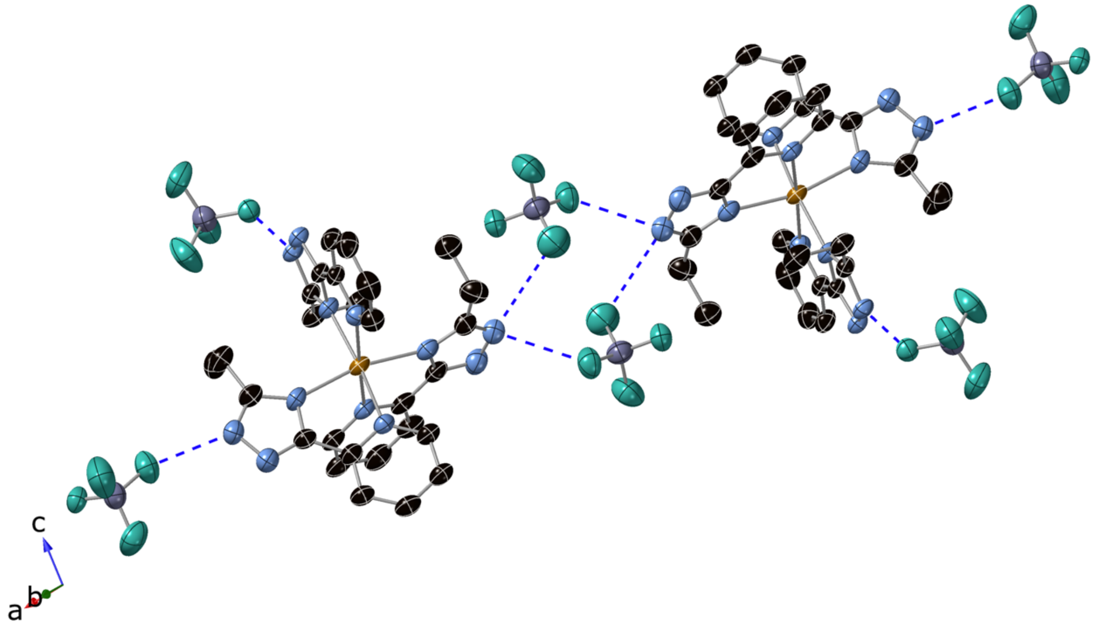

Figure S11. Hydrogen bonding between $[Fe(L1)_3]^{2+}$ complexes and BF_4^- counteranions in the structure of **1b[BF$_4$]$_2$** at 100 K. For clarity only one of two configurations of disordered counteranions is shown. Hydrogen bonds (blue-dashed lines), Fe (orange), C (black), N (blue), O(red), F(green) and B(gray).

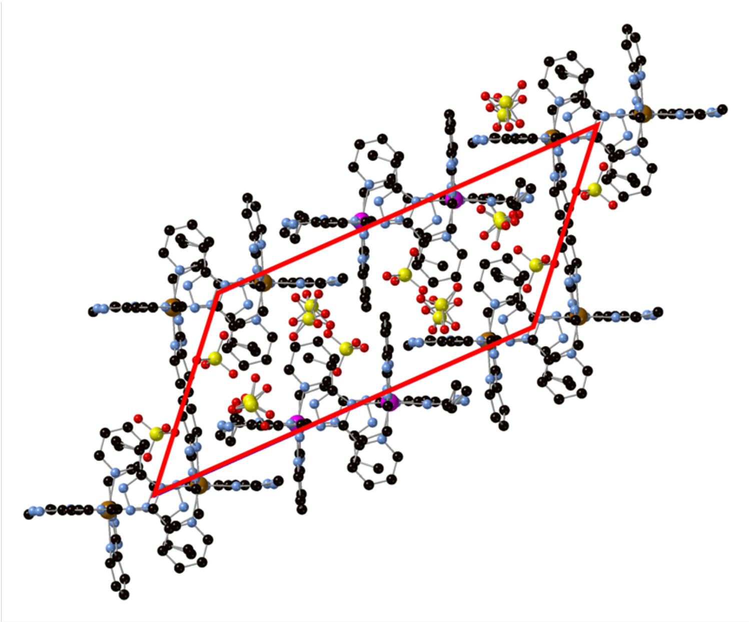

2HS phase (200 K)

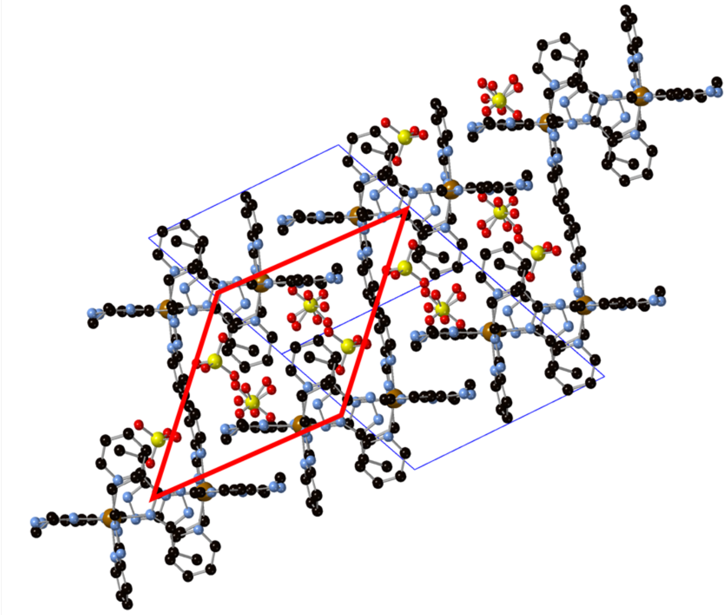

1HS phase (250 K)

Figure S12. Group-subgroup relationship in the 2HS and 1HS phases showing the a', b', 2c' lattice in the structure of **1[ClO$_4$]$_2$** at 200 K (2HS phase, top) and the a', b', c' lattice in the structure of **1[ClO$_4$]$_2$** at 250 K (1HS phase, bottom). The new a', b', 2c' and a', b', c' unit cells are depicted in red. Fe (orange), C (black), N (blue), O(red) and Cl(yellow). To differentiate the two non-equivalent Fe sites of the 2HS phase, they are depicted in purple and orange.

To describe the group-subgroup relationship between 1HS and 2HS phases, we have used another lattice common to both phases, as shown in Figure S12. Based on this, the 1HS to 2HS phase transition can be clearly seen as a cell doubling along the new c' axis.

Structures of $2[ClO_4]_2·EtOH$, $2[BF_4]_2·EtOH$ and $2[BF_4]_2$.

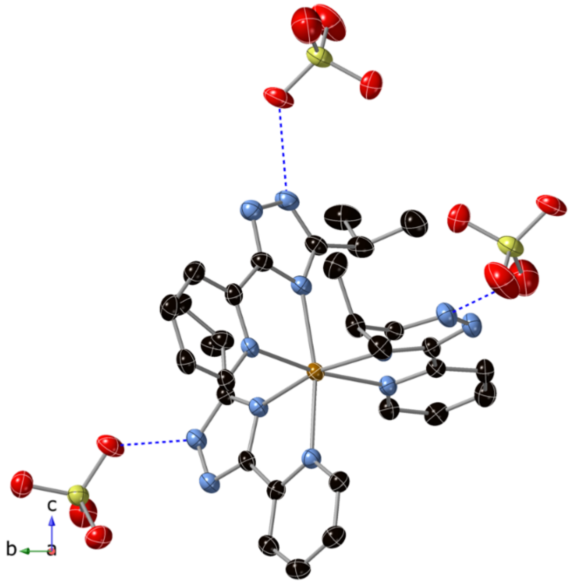

Figure S13 Projection of the structure in the *bc* plane of **$2[ClO_4]_2·EtOH$** at 120 K. For clarity, only half the disordered ClO_4^- and disordered ^{i}Pr groups of **$2[ClO_4]_2·EtOH$** is shown. Fe (orange) C (black), N (blue), Cl (yellow) and O (red). Hydrogen bonds are shown as blue-dashed lines.

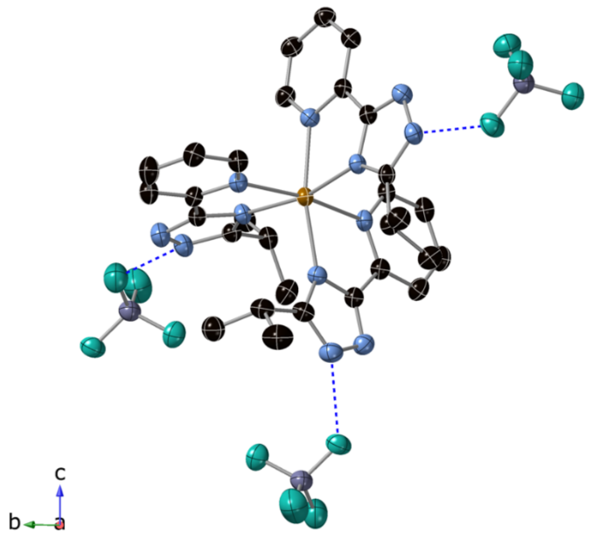

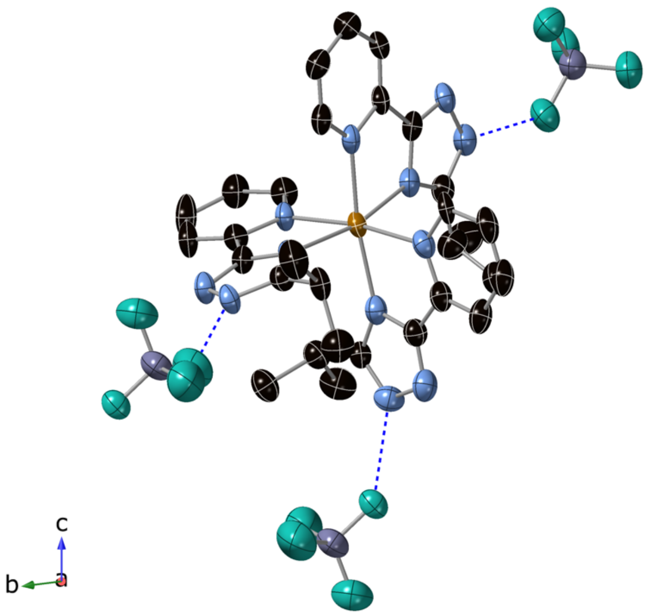

Figure S14 Projection of the structure in the *bc* plane of **2[BF$_4$]$_2$·EtOH** at 120 K (top) and **2[BF$_4$]$_2$** at 100 K (bottom). Fe (orange) C (black), N (blue), B(gray) and F (green). Hydrogen bonds are shown as blue-dashed lines.

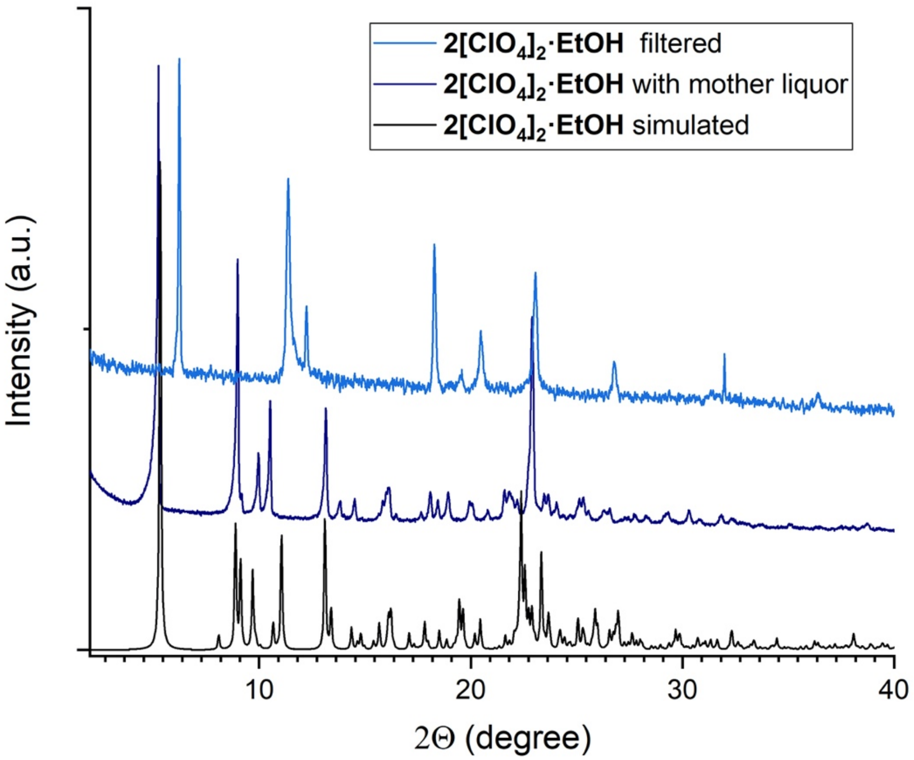

Figure S15 PXRD pattern of a filtered sample of **2[ClO$_4$]$_2$·EtOH** (blue line), **2[ClO$_4$]$_2$·EtOH** in contact with the mother liquor (dark blue line) and simulated one from the structure solved by single crystal X-ray diffraction at 120 K (black line).

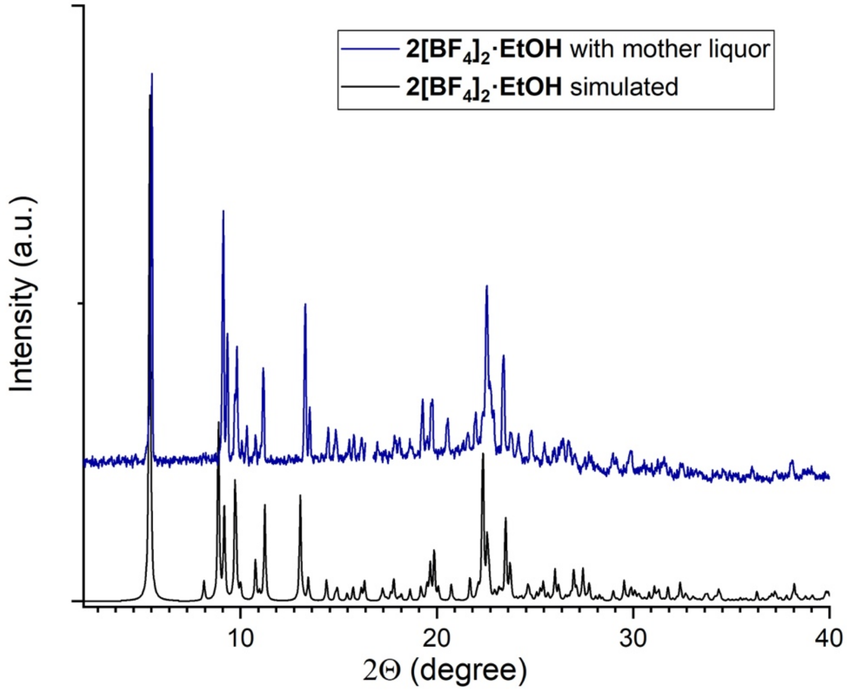

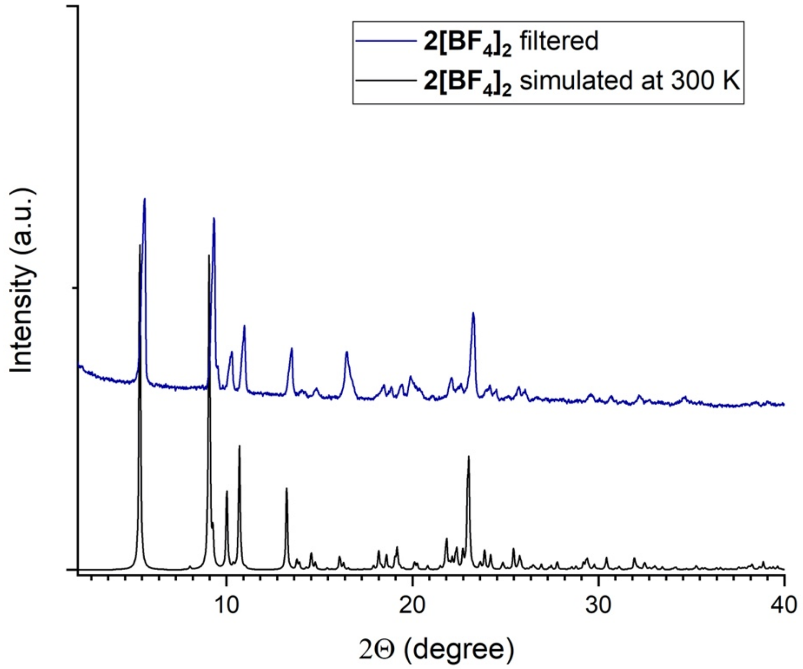

Figure S16 PXRD pattern of **$2[BF_4]_2$·EtOH** in contact with the mother liquor (blue line) and simulated one from the structure solved by single crystal X-ray diffraction at 120 K (black line) (top). Filtered sample of **$2[BF_4]_2$** (blue line) and simulated one from the structure solved by single crystal X-ray diffraction at 300 K (black line) (bottom).

Structure of $[Fe(L3)_3](BF_4)_2$·EtOH ($3[BF_4]_2$·EtOH)

The structure of **($3[BF_4]_2$·EtOH)** of formula $[Fe(L3)_3](BF_4)_2$·EtOH changes drastically with respect to that of **($3[ClO_4]_2$·0.5EtOH)**. In this case, there is only one crystallographically independent $[Fe(L3)_3]^{2+}$ complex with typical HS distances (average Fe-N distances of 2.178(13) Å at 120 K) consistent with magnetic measurements (see **Figure S18**). As in **($3[ClO_4]_2$·0.5EtOH)**, there is a disorder of one of the three **L3** ligands, which was solved with two possible configurations with occupancies of 0.88 and 0.12. In this case, both of them correspond to a *mer* configuration. Besides this, there are two BF_4^- counteranions and one EtOH molecule. Two of the three NH groups of **L3** ligand form hydrogen bonds with BF_4^- counteranions, while the third one is involved in a hydrogen bond with EtOH solvent molecule (see **Figure S18**). Neighboring $[Fe(L3)_3]^{2+}$ complexes interact through CH···π interactions between pyridine rings. PXRD pattern measured in contact with the mother liquor is consistent with the structure solved by single crystal X-ray diffraction. However, PXRD pattern of a filtered sample shows some new peaks which could be due to the coexistence of different solvates (see **Figure S20**). Indeed, elemental analysis suggests the replacement of the EtOH solvent molecule found in the structure by water molecules in the filtered sample (see experimental section).

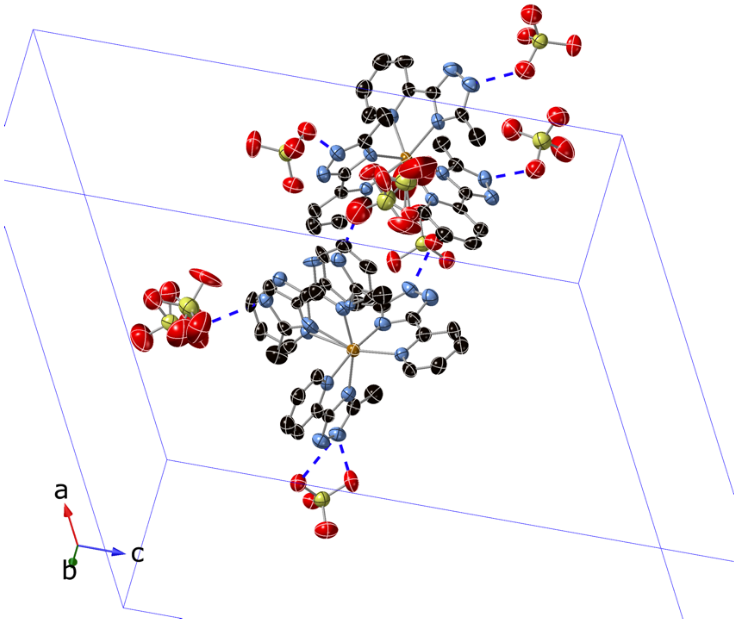

Figure S17 Structure of **($3[ClO_4]_2$·0.5EtOH)** at 120 K. Fe (orange) C (black), N (blue), Cl (yellow) and O (red). Hydrogen bonds are shown as blue-dashed lines.

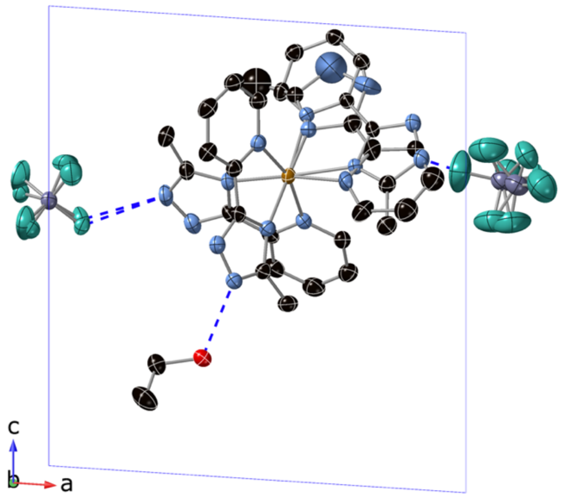

Figure S18 Projection of the structure in the *ac* plane of **(3[BF$_4$]$_2$·EtOH)** at 120. Fe (orange) C (black), N (blue), B(gray) and F (green). Hydrogen bonds are shown as blue-dashed lines.

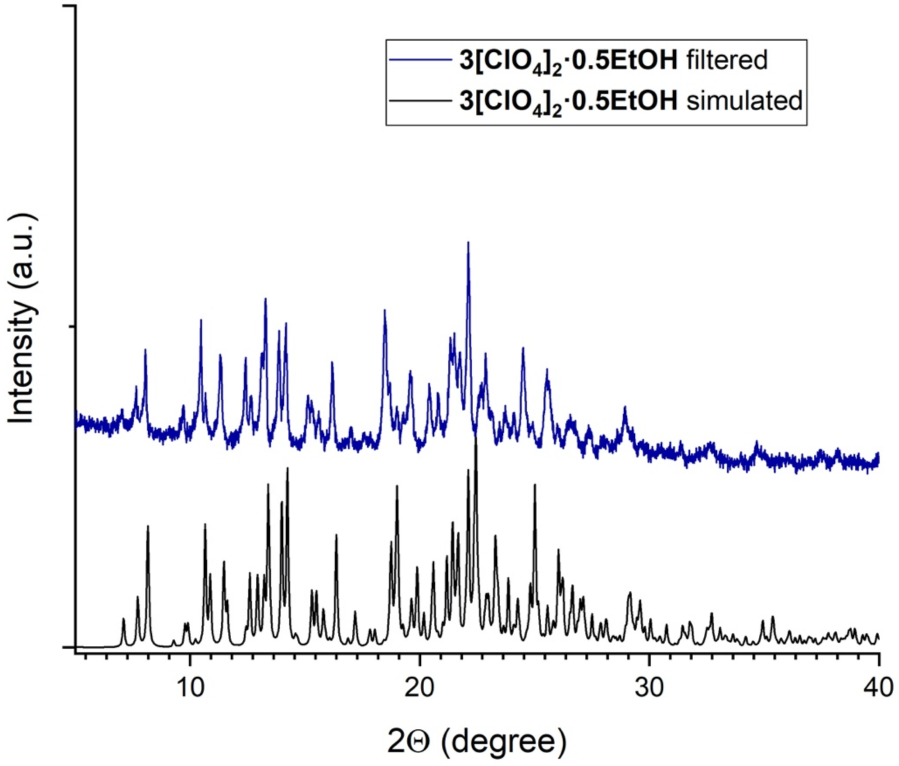

Figure S19 PXRD pattern of a filtered sample of **(3[ClO$_4$]$_2$·0.5EtOH)** (blue line) and simulated one from the structure solved by single crystal X-ray diffraction at 120 K (black line).

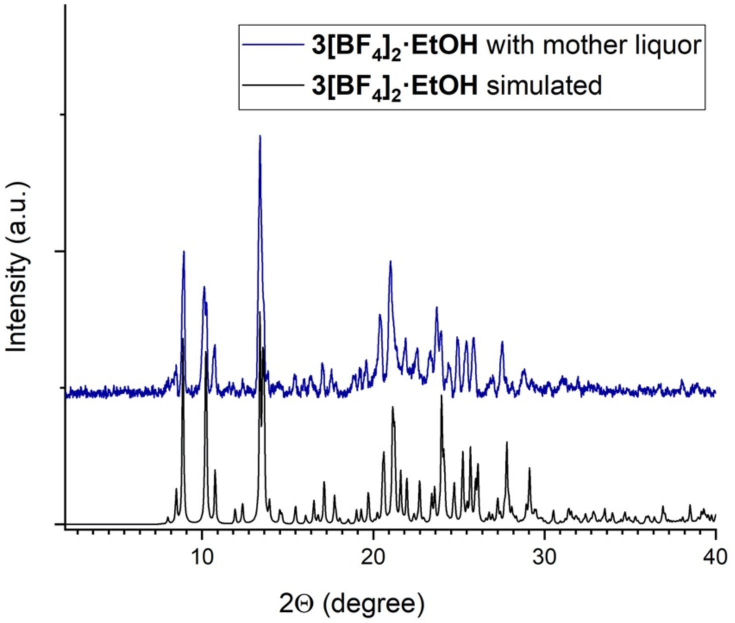

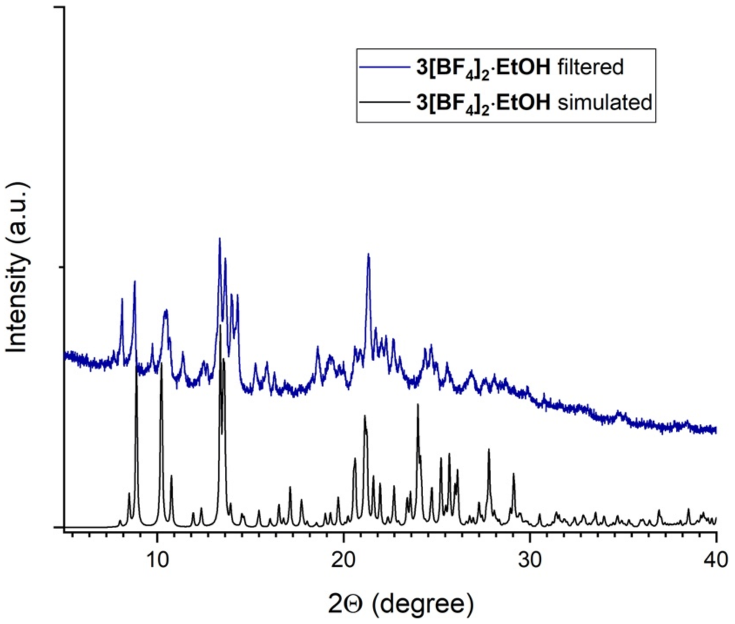

Figure S20 PXRD pattern of (**3[BF$_4$]$_2$·EtOH**) in contact with the mother liquor (blue line) (top) or in a filtered sample (blue line) (bottom). In the two figures the simulated pattern from the structure solved by single crystal X-ray diffraction at 120 K is shows as a black line.

Structure of $[Fe(L4)_3](ClO_4)_2\cdot Me_2CO\cdot H_2O$ ($4[ClO_4]_2\cdot Me_2CO\cdot H_2O$). **$4[ClO_4]_2\cdot Me_2CO\cdot H_2O$** crystallizes in the *P*-1 space group. It contains one crystallographically independent $[Fe(L4)_3]^{2+}$ complex in the asymmetric unit with typical HS Fe-N bond lengths (average = 2.1905(14) Å at 120 K in agreement with magnetic properties), two ClO_4^- counteranions and one water and acetone solvent molecules. In this case the three NH groups from triazole form hydrogen bonds with two water molecules and ClO_4^- counteranion (see **Figure S21**). Neighboring $[Fe(L4)_3]^{2+}$ complexes present short contacts between CH groups from pyridine and N from triazole, CH···π contacts between pyridine rings and short contacts between CH_2 groups from CH_2Ph substituents. The parallel disposition of the triazole and phenyl rings from the same ligand could suggest some type of interactions, although C-C and C-N distances (>3.5 Å) are higher than those observed in typical π-π stacking interactions. PXRD pattern of crystals in contact with the mother liquor is consistent with the structure solved from single crystal X-ray diffraction. On the contrary, PXRD of filtered crystals shows some differences with respect to the simulated one (see **Figure S22**). This suggests that structural changes are taking place after removing the crystals from the mother liquor.

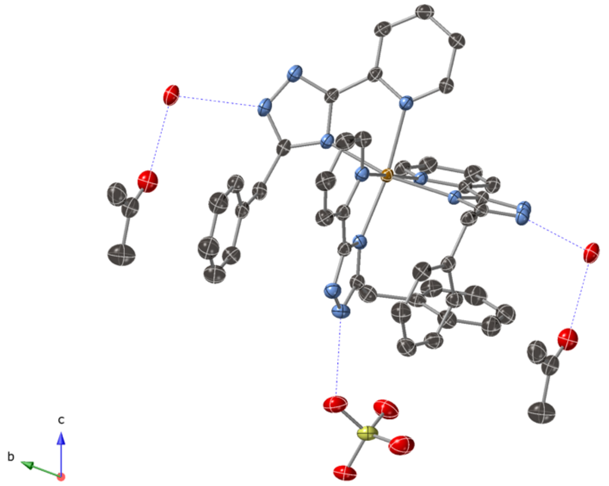

Figure S21 Projection of the structure in the *bc* plane of **$4[ClO_4]_2\cdot Me_2CO\cdot H_2O$** at 120 K. Fe (orange) C (black), N (blue), Cl (yellow) and O (red). Hydrogen bonds are shown as blue-dashed lines.

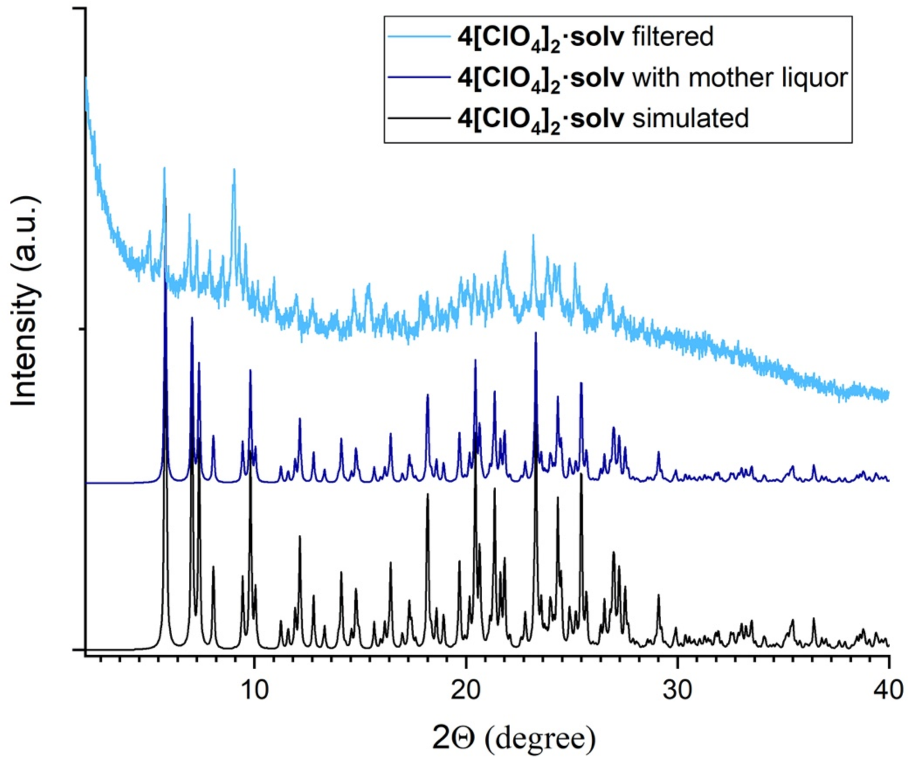

Figure S22 PXRD pattern of a filtered sample of **4[ClO$_4$]$_2$·Me$_2$CO·H$_2$O** (blue line), **4[ClO$_4$]$_2$·Me$_2$CO·H$_2$O** in contact with the mother liquor (dark blue line) and simulated one from the structure solved by single crystal X-ray diffraction at 120 K (black line).

Structure of [Fe(L5)$_3$](ClO$_4$)$_2$·EtOH (**5[ClO$_4$]$_2$·EtOH**) and **[Fe(L5)$_3$](BF$_4$)$_2$·EtOH (5[BF$_4$]$_2$·EtOH)**. Compounds **5[ClO$_4$]$_2$·EtOH** and **5[BF$_4$]$_2$·EtOH** crystallize in *P*-1 space group and contain one crystallographically independent [Fe(L5)$_3$]$^{2+}$ complex in the asymmetric unit and typical HS Fe-N bond lengths (average = 2.184(3) Å for (**5[ClO$_4$]$_2$·EtOH**) and 2.183(3) Å for (**5[BF$_4$]$_2$·EtOH**)) at 120 K. This agrees with magnetic properties. [Fe(L5)$_3$]$^{2+}$ complexes form hydrogen bonds with three ClO_4^- or BF_4^- anions through the NH groups (see **Figure S23**). They present short contacts between CH groups from pyridine and N from triazole and CH···π contacts between pyridine rings, as those observed in the previous compounds. Furthermore, they show short contacts between CH$_2$ groups from *c*-Pr and NH groups. EtOH solvent molecules were found to be disordered over several positions. PXRD patterns in contact with the mother liquor show a good agreement with the simulated one from single crystal X-ray diffraction. PXRD patterns of a freshly filtered sample of (**5[ClO$_4$]$_2$·EtOH**) show a good agreement with the simulated one, while in **(5[BF$_4$]$_2$·EtOH)** it was necessary to achieve this to perform the measurement in contact with the mother liquor (see **Figure S24**).

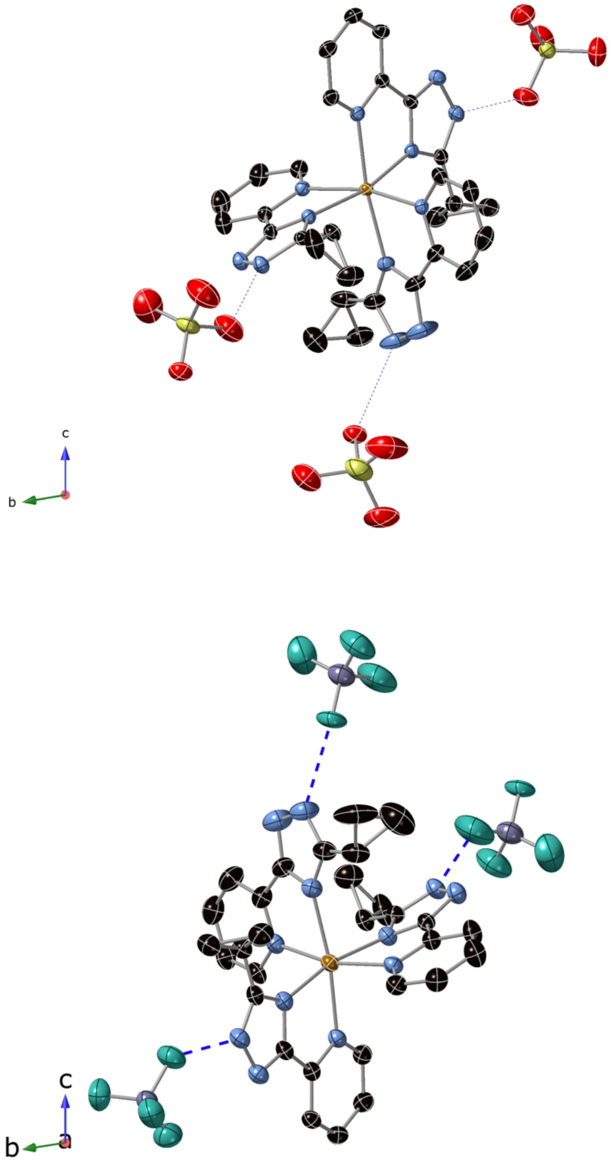

Figure S23 Projection of the structure in the bc plane of **($5[ClO_4]_2$·EtOH)** (top) and **($5[ClO_4]_2$·EtOH)** (bottom) at 120 K. Fe (orange) C (black), N (blue), B (gray), F (green), Cl (yellow) and O (red). Half disordered configuration of *c*-Pr substituents and ClO_4^- have been omitted for clarity. Hydrogen bonds are shown as blue-dashed lines.

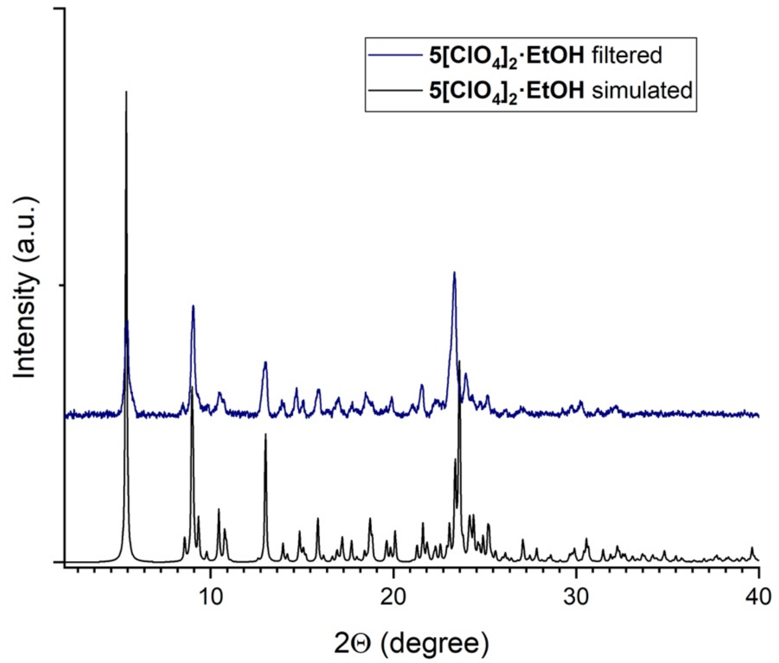

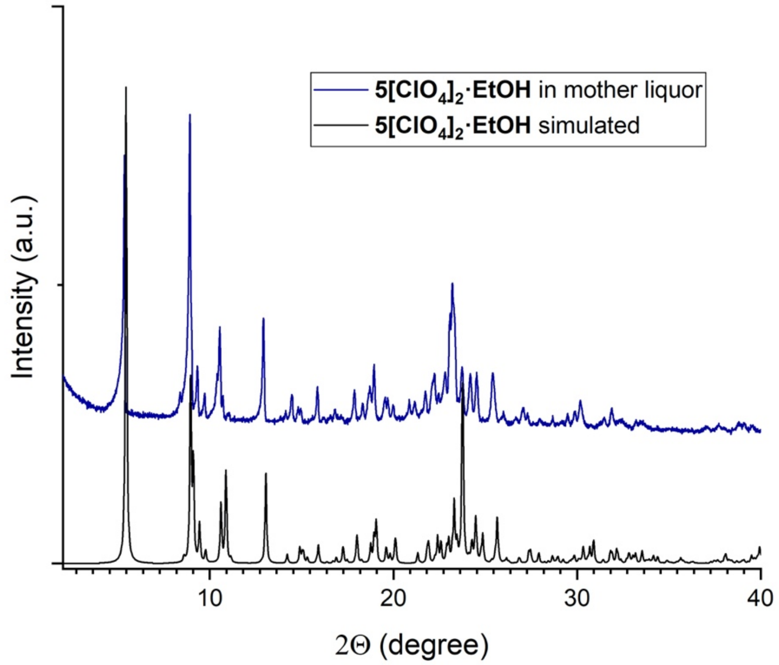

Figure S24PXRD pattern of a filtered sample of **($5[ClO_4]_2{\cdot}EtOH$)** (blue line) and simulated one from the structure solved by single crystal X-ray diffraction at 120 K (black line) (top). PXRD pattern of **($5[ClO_4]_2{\cdot}EtOH$)** (blue line) in contact with mother liquor and simulated one from the structure solved by single crystal X-ray diffraction at 120 K (black line) (bottom).

Structure of [Fe(L6)(ClO_4)$_2$·H_2O (6[ClO_4]$_2$·H_2O) and [Fe(L6)$_3$](BF_4)$_2$·H_2O (6[BF_4]$_2$·H_2O). Compound (**6[ClO_4]$_2$·H_2O**) crystallizes in the *P*-1 space group and contain two crystallographically independent [Fe(L6)$_3$]$^{2+}$ complexes in the asymmetric unit (Fe1 and Fe2 complexes) with typical HS Fe-N bond lengths (average = 2.137(4) Å for Fe1 and 2.188(4) Å for Fe2 and four ClO_4^- (two of them with a disorder solved with two possible configurations) at 120 K. The shorter Fe-N distances of Fe1 at 120 K suggest a small fraction of molecules in the LS state in agreement with the partial SCO observed in the magnetic properties (see below). Solvent molecules were found to be disordered over several positions and could not be modelled satisfactorily. They were removed from the electron density map using the OLEX solvent mask command.[7] The three NH from triazole groups of Fe1 form hydrogen bonds with ClO_4^- anions. In Fe2, only two of them present this type of hydrogen bonds, while the third one presents a hydrogen bond with C_5H_9O substituent from Fe1 (see **Figure S25**). Interactions between neighboring complexes involve short contacts between CH groups from pyridine and N from triazole and short contacts between C_5H_9O substituents and pyrazole, pyridine and other C_5H_9O substituents. PXRD patterns of the two compounds in contact with the mother liquor is consistent with the structure solved by single crystal X-ray diffractions in (**6[ClO_4]$_2$·H_2O)** proving that they are isostructural, while those of the filtered samples show small differences (see **Figure S26**), which could be due to the replacement of the solvent molecules found in the structure by water molecules after filtering as shown by elemental analysis (see experimental section).

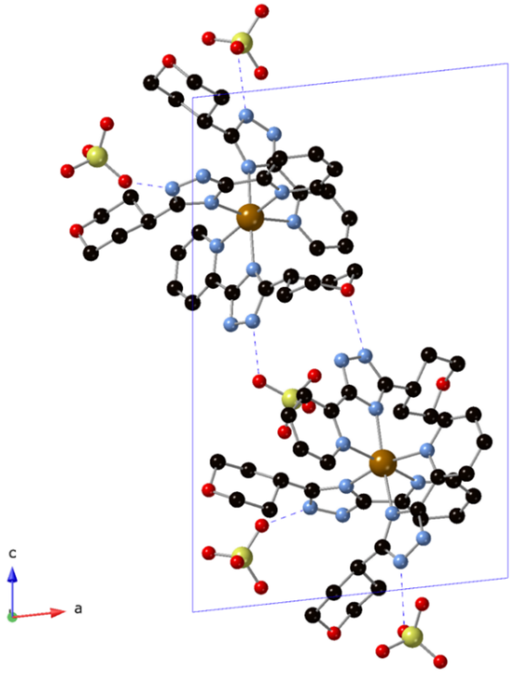

Figure S25 Projection of the structure in the *bc* plane of (**6[ClO_4]$_2$·H_2O** at 120 K. Fe (orange) C (black), N (blue), Cl (yellow) and O (red). Half disordered configuration of ClO_4^- has been omitted for clarity. Hydrogen bonds are shown as blue-dashed lines.

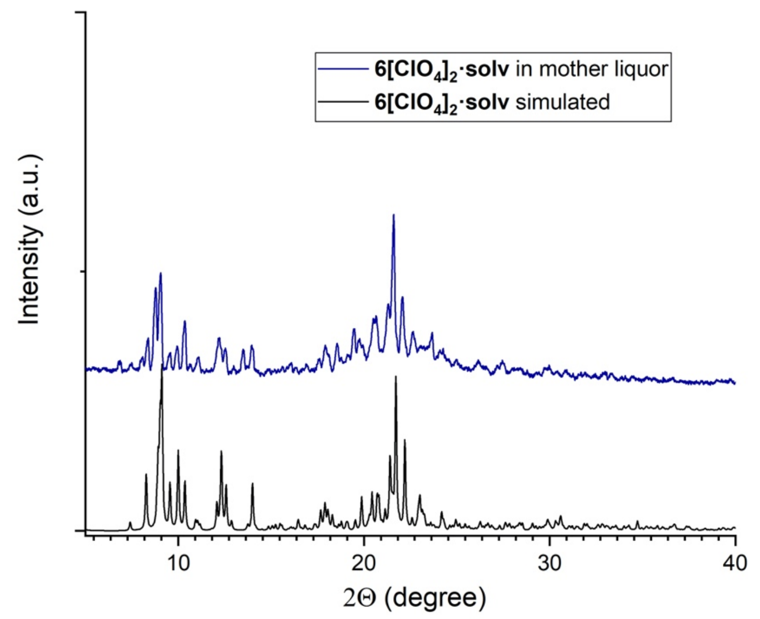

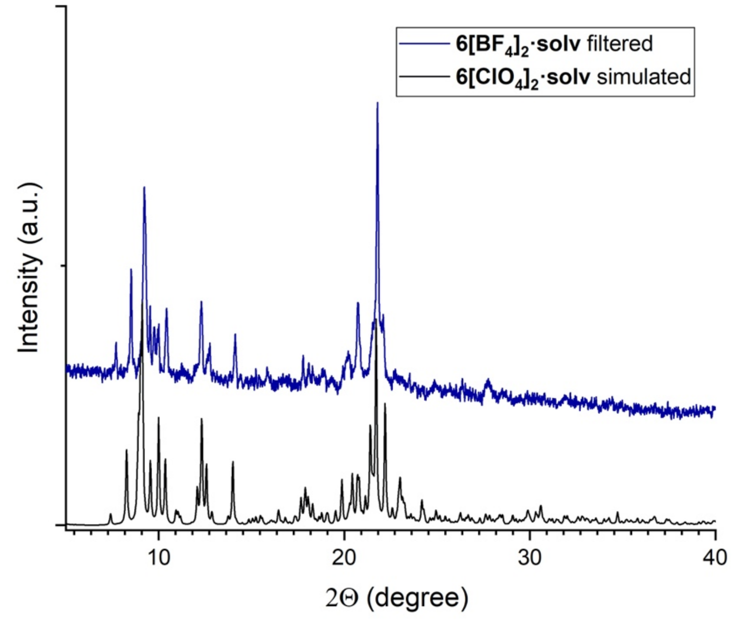

Figure S26 PXRD pattern of a **($6[ClO_4]_2{\cdot}H_2O$)** measured in contact with the mother liquor (blue line) and simulated one from the structure solved by single crystal X-ray diffraction at 120 K (black line) (top). PXRD pattern of a freshly filtered sample of **$6[BF_4]_2{\cdot}H_2O$** (blue line) and simulated one from the structure of **($6[ClO_4]_2{\cdot}H_2O$)** solved by single crystal X-ray diffraction at 120 K (black line) (bottom).

3. Magnetic characterization of the coordination compounds

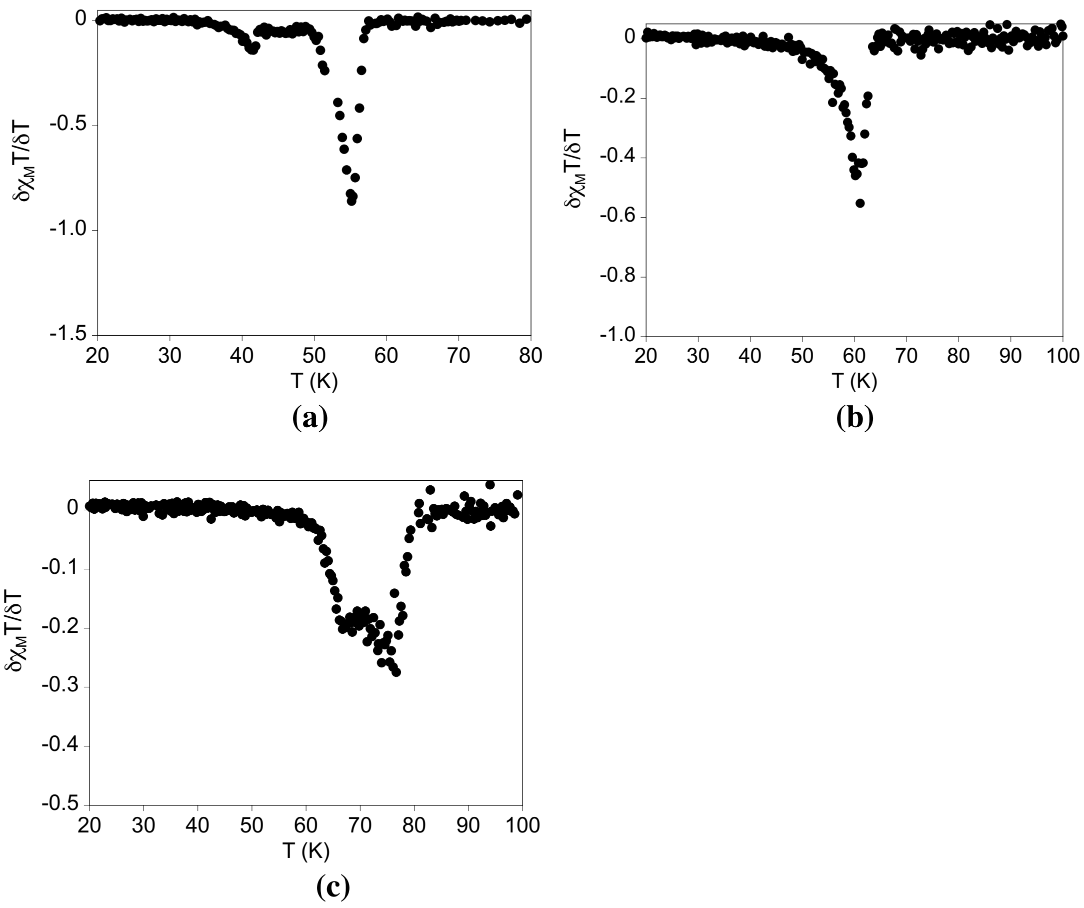

Figure S27 Derivative of χ_MT vs. T after light excitation of **1[ClO$_4$]$_2$** (a) and **1[BF$_4$]$_2$** (b) and quick cooling to 10 K of **1[BF$_4$]$_2$** (c).

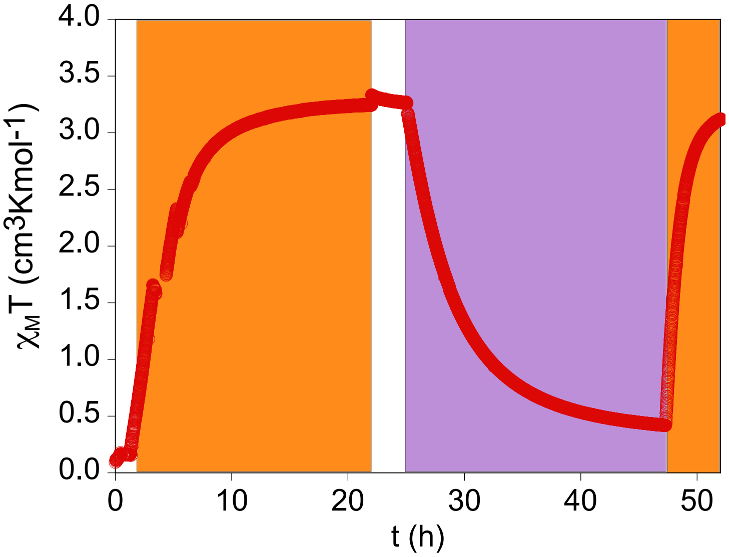

Figure S28 Reverse LIESST experiment. χ_MT vs. time in hours after light excitation with 660 nm (red background) and 808 nm (purple background) of **1[ClO$_4$]$_2$** at 10 K. White background corresponds to a period with no irradiation of **1[ClO$_4$]$_2$** at 10 K.

Magnetic properties of ($3[ClO_4]_2·0.5EtOH$), ($3[BF_4]_2·EtOH$), ($2[ClO_4]_2·EtOH$), ($4[ClO_4]_2·Me_2CO·H_2O$), ($5[ClO_4]_2·EtOH$), ($5[BF_4]_2·EtOH$), $6[ClO_4]_2·H_2O$ and $6[BF_4]_2·H_2O$

Magnetic properties of filtered samples of **$3[ClO_4]_2·0.5EtOH$**, **$2[ClO_4]_2·EtOH$**, **$4[ClO_4]_2·Me_2CO·H_2O$**, **$5[ClO_4]_2·EtOH$** and **$5[BF_4]_2·EtOH$** suggest a predominant HS state as they show $\chi_M T$ values close to 3.5 cm^3Kmol^{-1} in the 50-400 K temperature range (see **Figure S29**). In **$2[ClO_4]_2·EtOH$** and **$5[BF_4]_2·EtOH$**, gradual and incomplete spin transitions are observed when measured in contact with the mother liquor (see **Figures S30** and **S31**). Irradiation of **$2[ClO_4]_2·EtOH$,** protected with a grease to avoid desolvation, at 10 K with 660 nm leads to a LIESST effect with an almost complete photoconversion of the 30 % of LS centers at this temperature with a T(LIESST) of 54 K (see **Figure S31**). In **$4[ClO_4]_2·Me_2CO·H_2O$**, $\chi_M T$ values close to 3.5 cm^3Kmol^{-1} are observed from 400 to 300 K after heating to 400 K. They decrease gradually at lower temperatures to reach 2.2 cm^3Kmol^{-1} at 50 K corresponding to an incomplete spin transition of around 40 % of the HS Fe(II) (see **Figure S32**).

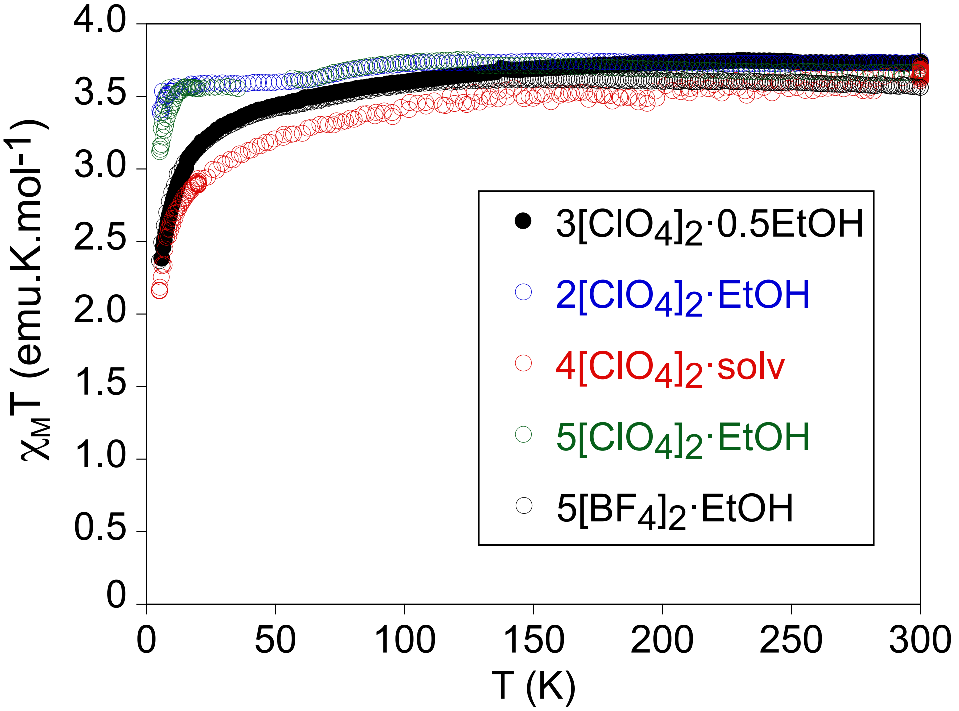

Figure S29 Thermal dependence of χ_MT of filtered samples of **$3[ClO_4]_2·0.5EtOH$,** (full black circles), **$2[ClO_4]_2·EtOH$** (empty blue circles), **$4[ClO_4]_2·Me_2CO·H_2O$** (empty red circles), **$5[ClO_4]_2·EtOH$** (empty green circles) and **$5[BF_4]_2·EtOH$** (empty black circles).

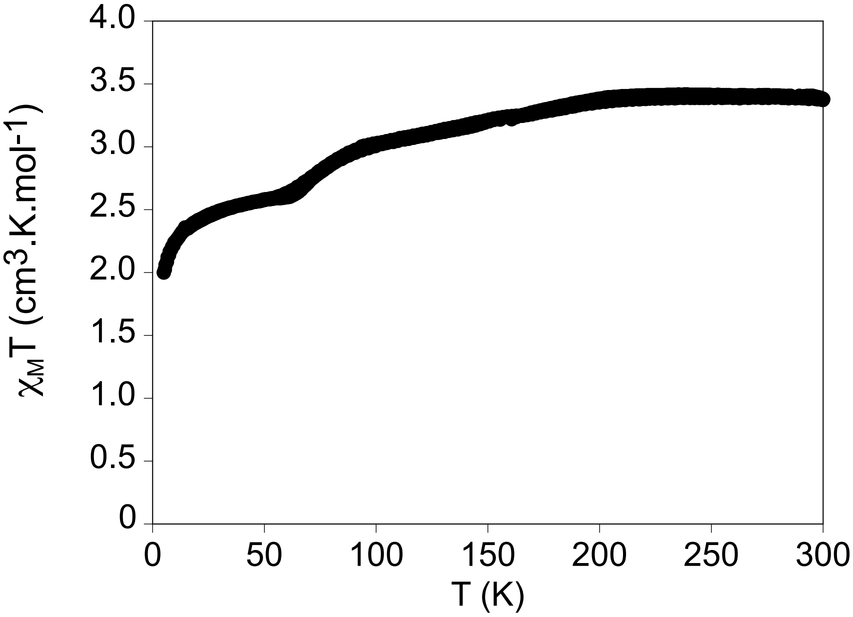

Figure S30 Thermal dependence of χ_MT of **(5[BF$_4$]$_2$·EtOH)** measured in contact with the mother liquor.

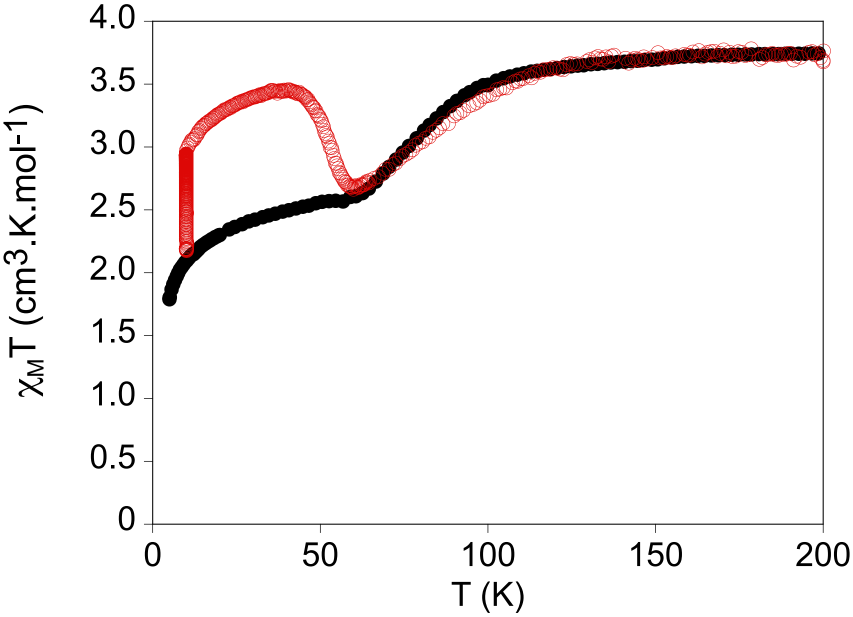

Figure S31 Thermal dependence of χ_MT of **(2[ClO$_4$]$_2$·EtOH),** protected with a grease to avoid desolvation. Full circles: data recorded without irradiation; empty red circles: data recorded after irradiation at 10 K.

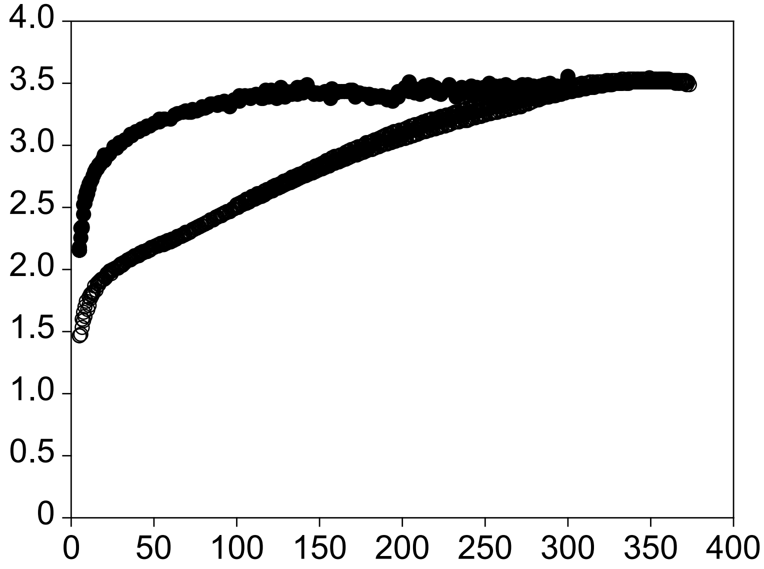

Figure S32 Thermal dependence of $\chi_M T$ of filtered sample of (**4[ClO$_4$]$_2$·Me$_2$CO·H$_2$O**) before (full black circles) and after (empty black circles) being heated to 400 K.

Magnetic properties of **3[BF$_4$]$_2$·EtOH**, **6[ClO$_4$]$_2$·H$_2$O** and **6[BF$_4$]$_2$·H$_2$O** measured in a filtered sample or in contact with the mother liquor show gradual and incomplete spin transitions with the temperature (see **Figures S33** and **S34**). For **3[BF$_4$]$_2$·EtOH**, the most complete SCO was achieved in the desolvated sample heated previously at 400 K, with a gradual decrease of $\chi_M T$ from 3.8 to 1.5 cm^{3}Kmol^{-1} in the 300-50 K temperature range suggesting a spin transition of around 60 % of the HS centers at 300 K. This sample show a LIESST effect with a complete photoconversion and a T(LIESST) of 55 K (see **Figure S33**). The gradual and incomplete spin transition of **3[BF$_4$]$_2$·EtOH** is in agreement with the behavior found in the literature for other salts of Fe(L3)$_3$]$^{2+}$ complexes such as [Fe(L3)$_3$](PF$_6$)$_2$·H$_2$O compound and contrast with the complete spin transition found in [Fe(L3)$_3$](ClO$_4$)$_2$·H$_2$O.[8] The structure of these two compounds was not reported.

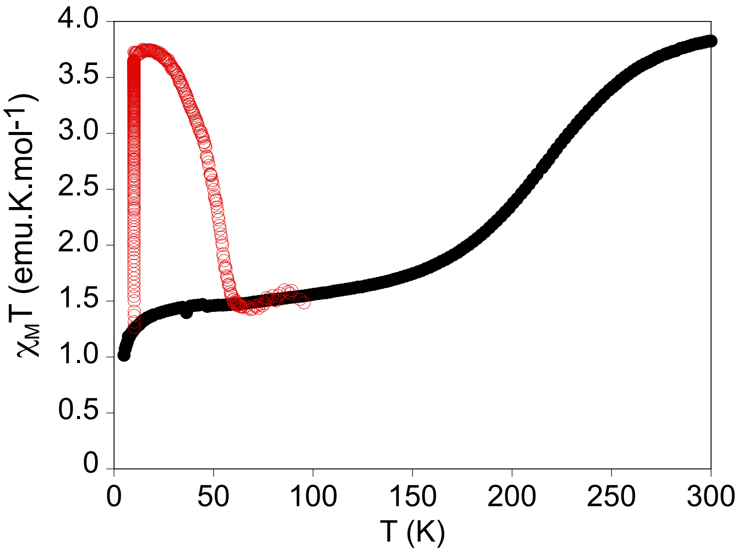

Figure S33 Thermal dependence of $\chi_M T$ of a filtered sample of **3[BF$_4$]$_2$·EtOH** previously heated to 400 K. Full circles: data recorded without irradiation; empty red circles: data recorded after irradiation at 10 K.

In **6[ClO$_4$]$_2$·H$_2$O** and **6[BF$_4$]$_2$·H$_2$O**, partial and incomplete spin transitions are obtained for filtered and solvated samples. In **6[ClO$_4$]$_2$·H$_2$O**, both samples display a very similar magnetic behavior with a gradual decrease of $\chi_M T$ from almost constant values close to 7.2 cm^{3}Kmol^{-1} above 200 K, consistent with the two crystallographically independent HS [Fe(L6)$_3$]$^{2+}$ molecules found in the structure, to 4.8 cm^{3}Kmol^{-1} at 50 K (see **Figures S34** and **S35**). This suggests a partial SCO of around 1/3 of the HS molecules. In **6[BF$_4$]$_2$·H$_2$O**, a more complete SCO is obtained for the filtered sample previously heated to 400 K (see **Figure S34**). Thus, the gradual decrease of $\chi_M T$ from 7.4 to 3.4 cm^{3}Kmol^{-1} in the 300-50 K temperature range suggests a more complete SCO of around 50 % of the HS molecules. Irradiation at 10 K at 660 nm of filtered **6[ClO$_4$]$_2$·H$_2$O** and **6[BF$_4$]$_2$·H$_2$O** induces an almost complete LS to HS photoconversion with T(LIESST) close to 50 K (see **Figure S34**).

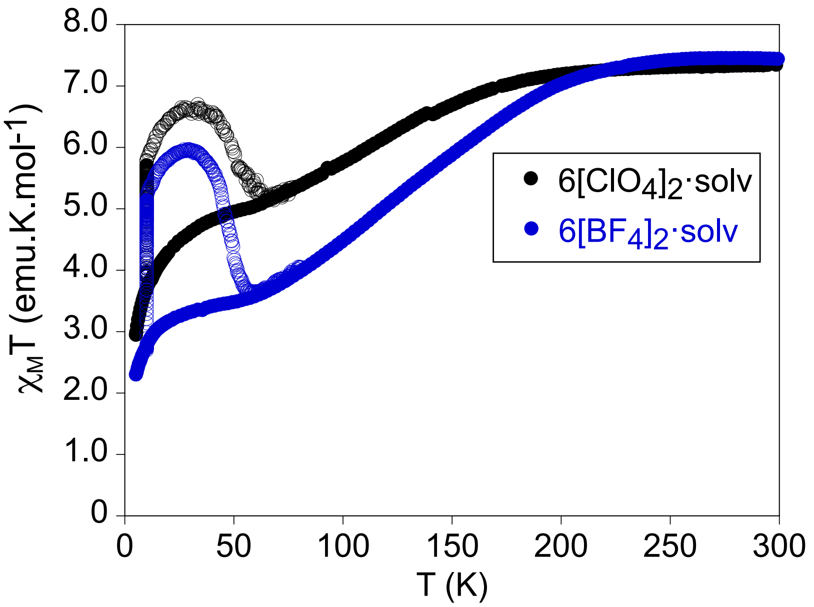

Figure S34 Thermal dependence of $\chi_M T$ of filtered samples of **6[ClO$_4$]$_2$·H$_2$O** (black circles) and **6[BF$_4$]$_2$·H$_2$O** previously heated to 400 K (blue circles). Full circles: data recorded without irradiation; empty circles: data recorded after irradiation at 10 K.

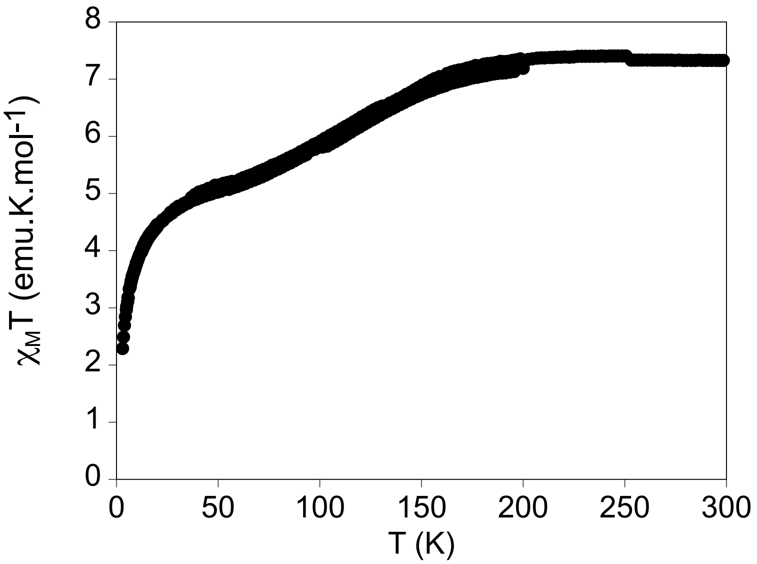

Figure S35 Thermal dependence of $\chi_M T$ of **6[ClO$_4$]$_2$·H$_2$O** measured in contact with the mother liquor.

1. Zakharchenko, B. V.; Khomenko, D. M.; Doroshchuk, R. O.; Severynovska, O. V.; Raspertova, I. V.; Starova, V. S.; Lampeka, R. D. Influence of the Nature of the Substituent in 3-(2-Pyridyl)-1,2,4-Triazole for Complexation with Pd2+. *Chem. Pap.* **2017**, *71* (10), 2003–2009. https://doi.org/10.1007/S11696-017-0194-8.
2. García-López, J.; Khomenko, D. M.; Zakharchenko, B. V.; Doroshchuk, R. O.; Starova, V. S.; Iglesias, M. J.; Lampeka, R. D.; López-Ortiz, F. Solvent- and Functional-Group-Assisted Tautomerism of 3-Alkyl Substituted 5-(2-Pyridyl)-1,2,4-Triazoles in DMSO–Water. *Org. Biomol. Chem.* **2023**, *21* (47), 9443–9458. https://doi.org/10.1039/D3OB01651J.

3. Khomenko, D. M.; Doroshchuk, R. O.; Vashchenko, O. V.; Lampeka, R. D. Synthesis and Study of Novel 1,2,4-Triazolylacetic Acid Derivatives. *Chem. Heterocycl. Compd.* **2016**, *52* (6), 402–408. https://doi.org/10.1007/s10593-016-1901-z.
4. Sheldrick, G. M. SHELXT - Integrated Space-Group and Crystal-Structure Determination. *Acta Crystallogr. Sect. A Found. Crystallogr.* **2015**, *71* (1), 3–8. https://doi.org/10.1107/S2053273314026370.
5. Sheldrick, G. M. Crystal Structure Refinement with SHELXL. *Acta Crystallogr. Sect. C* **2015**, *71* (1), 3–8. https://doi.org/10.1107/S2053229614024218.
6. Dolomanov, O. V.; Bourhis, L. J.; Gildea, R. J.; Howard, J. A. K.; Puschmann, H. OLEX2: A Complete Structure Solution, Refinement and Analysis Program. *J. Appl. Crystallogr.* **2009**, *42* (2), 339–341. https://doi.org/10.1107/S0021889808042726.
7. Dolomanov, O. V.; Bourhis, L. J.; Gildea, R. J.; Howard, J. A. K.; Puschmann, H. OLEX2: A Complete Structure Solution, Refinement and Analysis Program. *J. Appl. Crystallogr.* **2009**, *42* (2), 339–341. https://doi.org/10.1107/S0021889808042726.
8. Stupik, P.; Hage, R.; Jacobs, J.; Haasnoot, J. G.; Reedijk, J. Structural and magnetic (spin-crossover) phase transitions in a series of methyl substituted 5-(pyridine-2-YL)-1,2,4-triazole complexes of iron(II). *Hyperfine Interact.* **1988**, *40*, 343–346. https://doi.org/10.1007/BF02049113
